# Supplementary material for: Penisimplicins A and B: Novel Polyketide–Peptide Hybrid Alkaloids from the Fungus Penicillium simplicissimum JXCC5
Source: Molecules. 2024 Jan 27;29(3):613. doi: 10.3390/molecules29030613 (PMC10856265; doi:10.3390/molecules29030613)
Supplement: Supplementary file 1 [file molecules-29-00613-s001.zip › molecules-2724376-supplementary.pdf]

Supporting Information for "**Penisimplicins A and B: Novel Polyketide–Peptide Hybrid Alkaloids from the Fungus *Penicillium simplicissimum* JXCC5**"

Qing-Yuan Wang [1], Yang Gao [2], Jian-Neng Yao [3], Li Zhou [2,*], He-Ping Chen [2,*] and Ji-Kai Liu [2,*]

[1] School of Chemistry and Materials Science, South-Central Minzu University, Wuhan 430074, China
[2] School of Pharmaceutical Sciences, South-Central Minzu University, Wuhan 430074, China
[3] Yunnan Key Laboratory of Pharmacology for Natural Products & School of Pharmaceutical Science, Kunming Medical University, Kunming 650500, China; yaojianneng@kmmu.edu.cn
***** Correspondence: zhou2018@scuec.edu.cn (L.Z.); chenhp@mail.scuec.edu.cn (H.-P.C.); liujikai@mail.scuec.edu.cn (J.-K.L.)

Contents

1. Supplementary Figures 2
Figure S1. ^{1}H NMR spectrum of 1 (600 MHz, DMSO-d_6). 2
Figure S2. ^{13}C and DEPT NMR spectra of 1 (150 MHz, DMSO-d_6). 3
Figure S3. HSQC spectrum of 1. 4
Figure S4. ^{1}H-^{1}H COSY spectrum of 1. 5
Figure S5. HMBC spectrum of 1. 6
Figure S6. ROESY spectrum of 1. 7
Figure S7. Entire and partly (+)-HRESIMS spectrum of 1. 8
Figure S8. CD and UV spectra of compound 1 9
Figure S9. ^{1}H NMR spectrum of 2 (600 MHz, DMSO-d_6). 10
Figure S10. ^{13}C NMR spectrum of 2 (150 MHz, DMSO-d_6). 11
Figure S11. HSQC spectrum of 2 12
Figure S12. ^{1}H-^{1}H COSY spectrum of 2. 13
Figure S13. HMBC spectrum of 2. 14
Figure S14. ROESY spectrum of 2. 15
Figure S15. Enlarged ROESY spectrum of 2. 16
Figure S16. Entire and partly (+)-HRESIMS spectrum of 2. 17
Figure S17. CD and UV spectra of compound 2 18
Figure S18. Acetylcholinesterase Inhibitory activity of 1. 19
Figure S19. LC-MS analysis of crude extract (TIC). 19
2. Computational Details 20
2.1 ECD calculation details of compound 1 20
2.2 NMR calculation details of compound 1 21
2.2 NMR calculation details of compound 2 25
2.3 Standard orientations 29

S1. Supplementary Figures

Figure S1. ^{1}H NMR spectrum of **1** (600 MHz, DMSO-d_6).

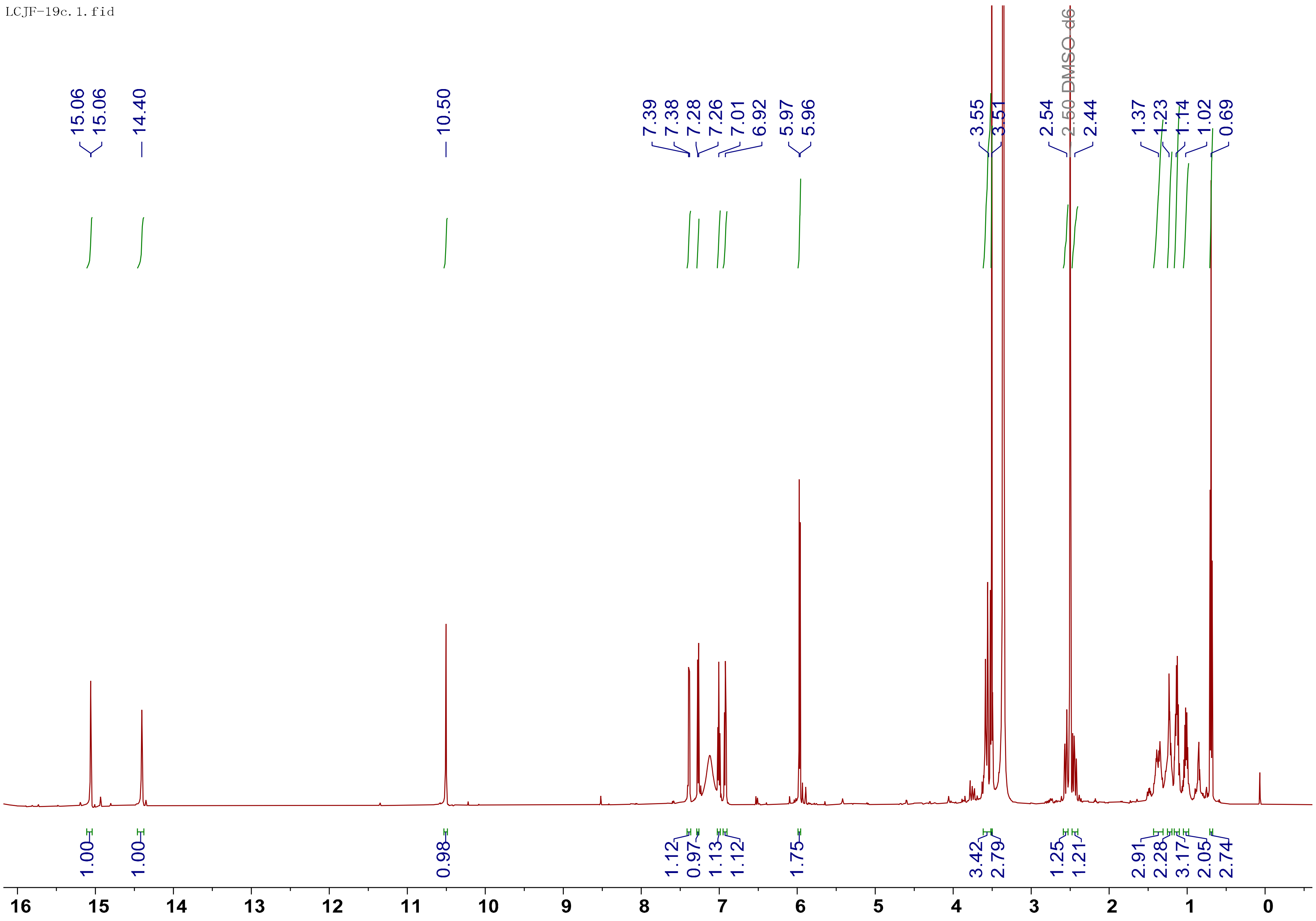

Figure S2. ^{13}C and DEPT NMR spectra of **1** (150 MHz, DMSO-d_6).

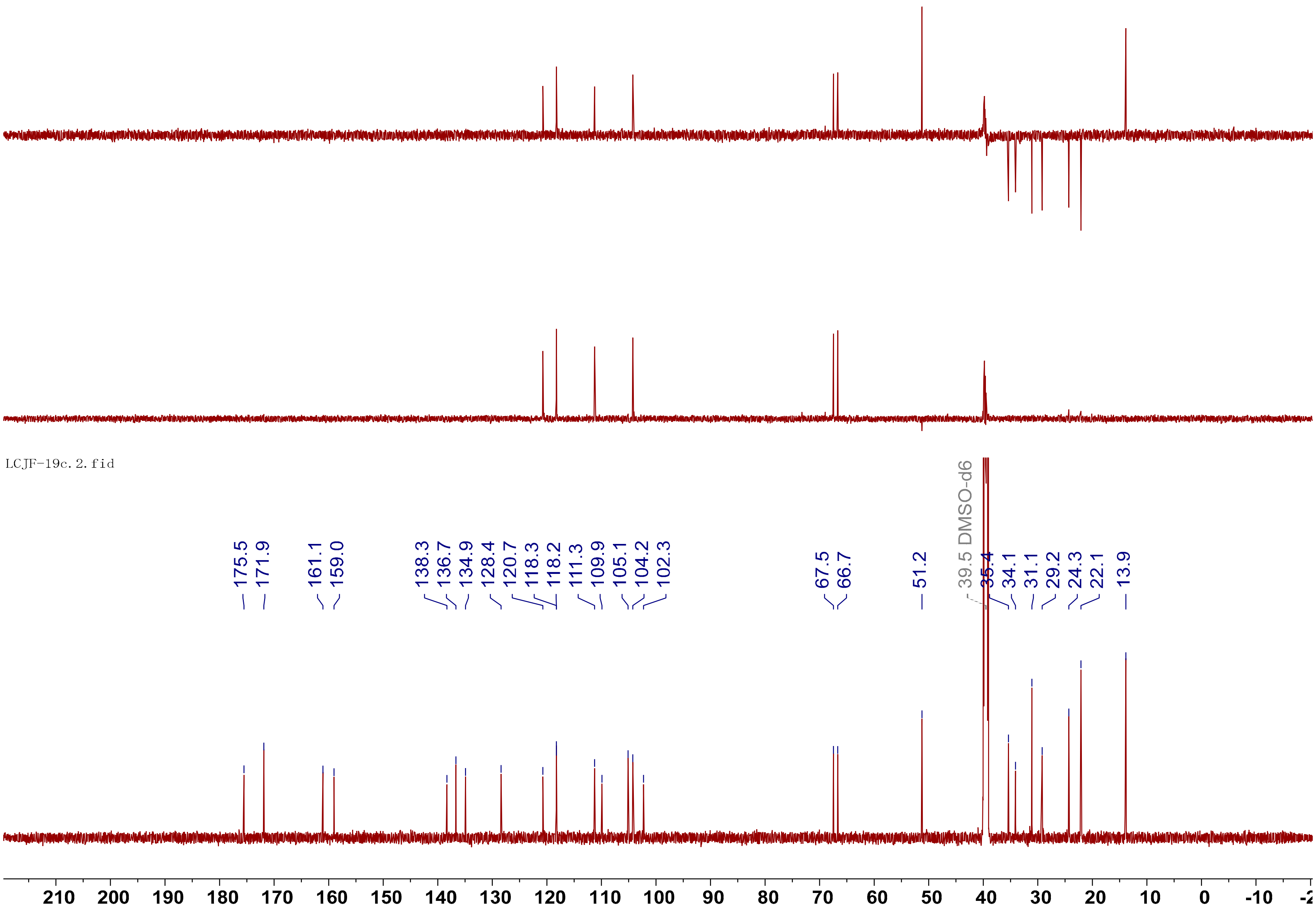

Figure S3. HSQC spectrum of **1**.

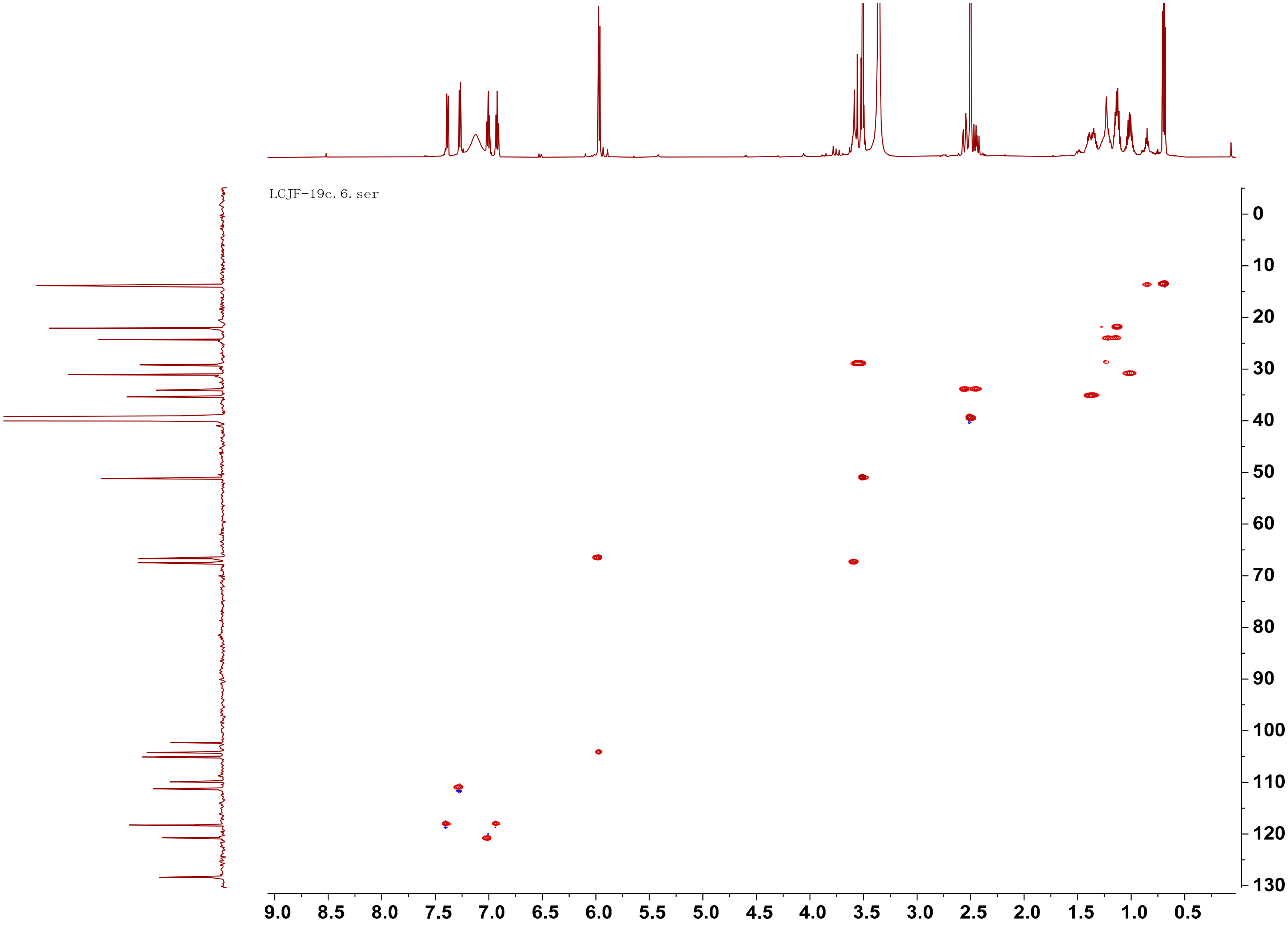

Figure S4. ^{1}H-^{1}H COSY spectrum of **1**.

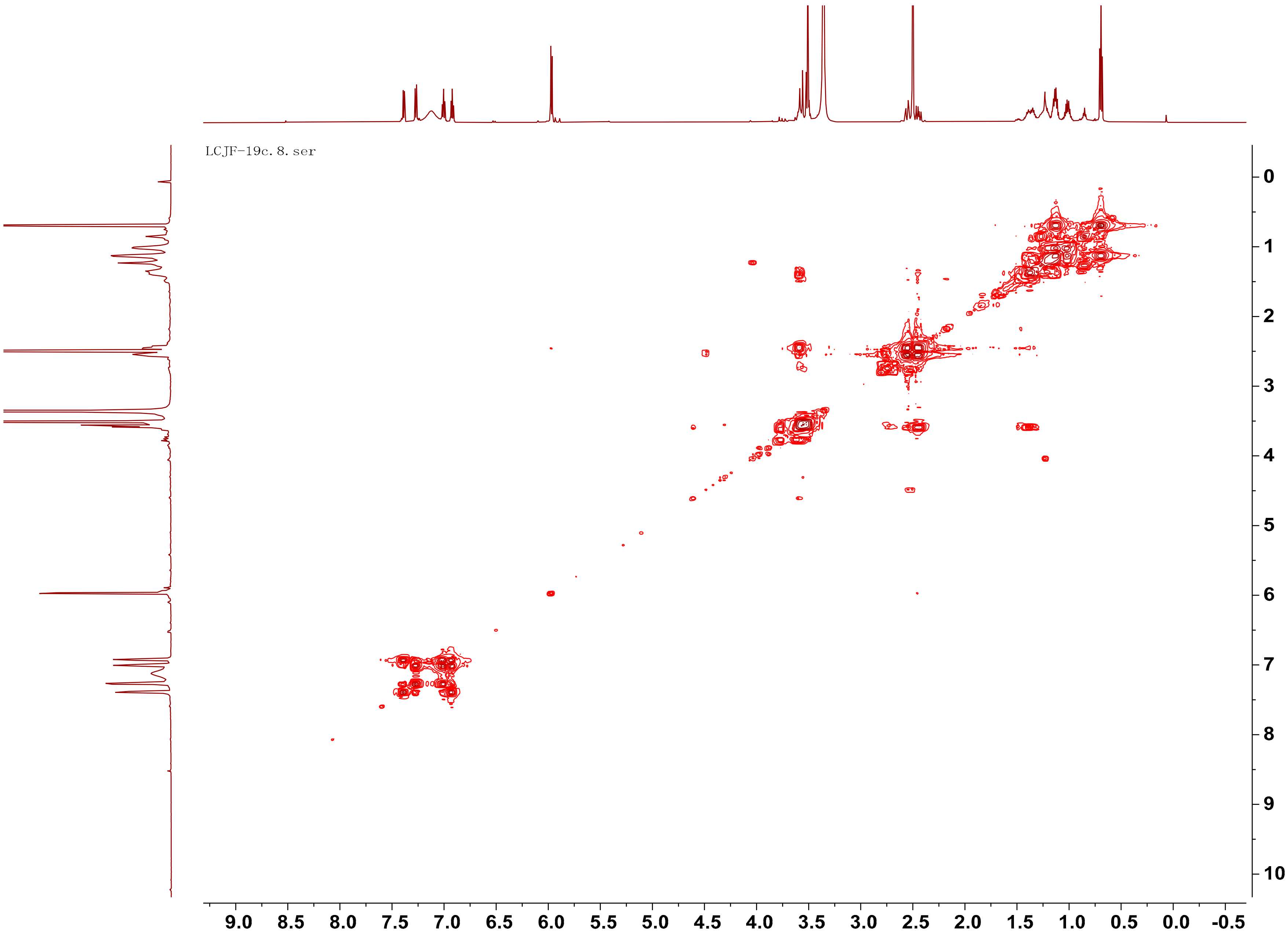

Figure S5. HMBC spectrum of **1**.

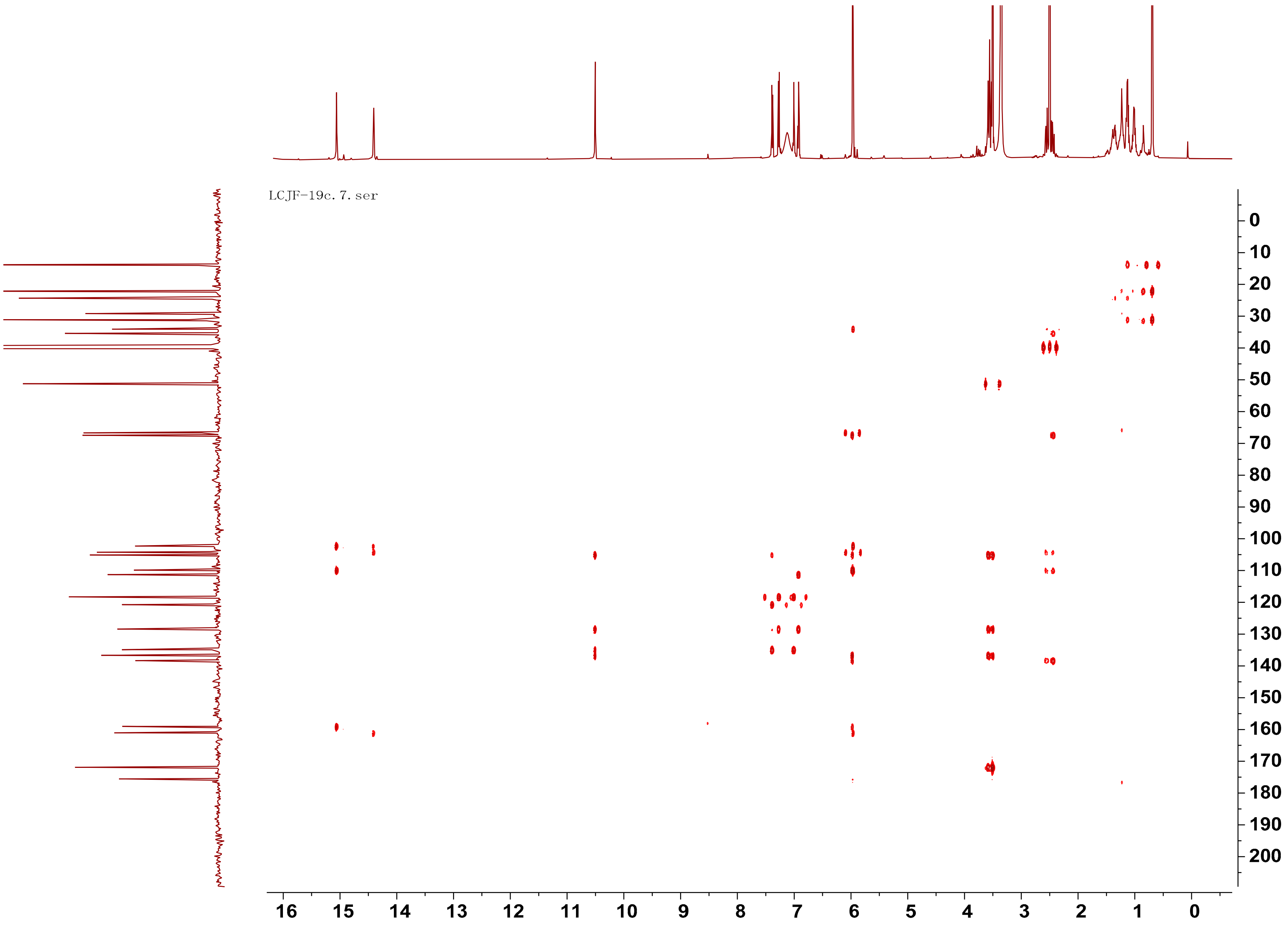

Figure S6. ROESY spectrum of **1**.

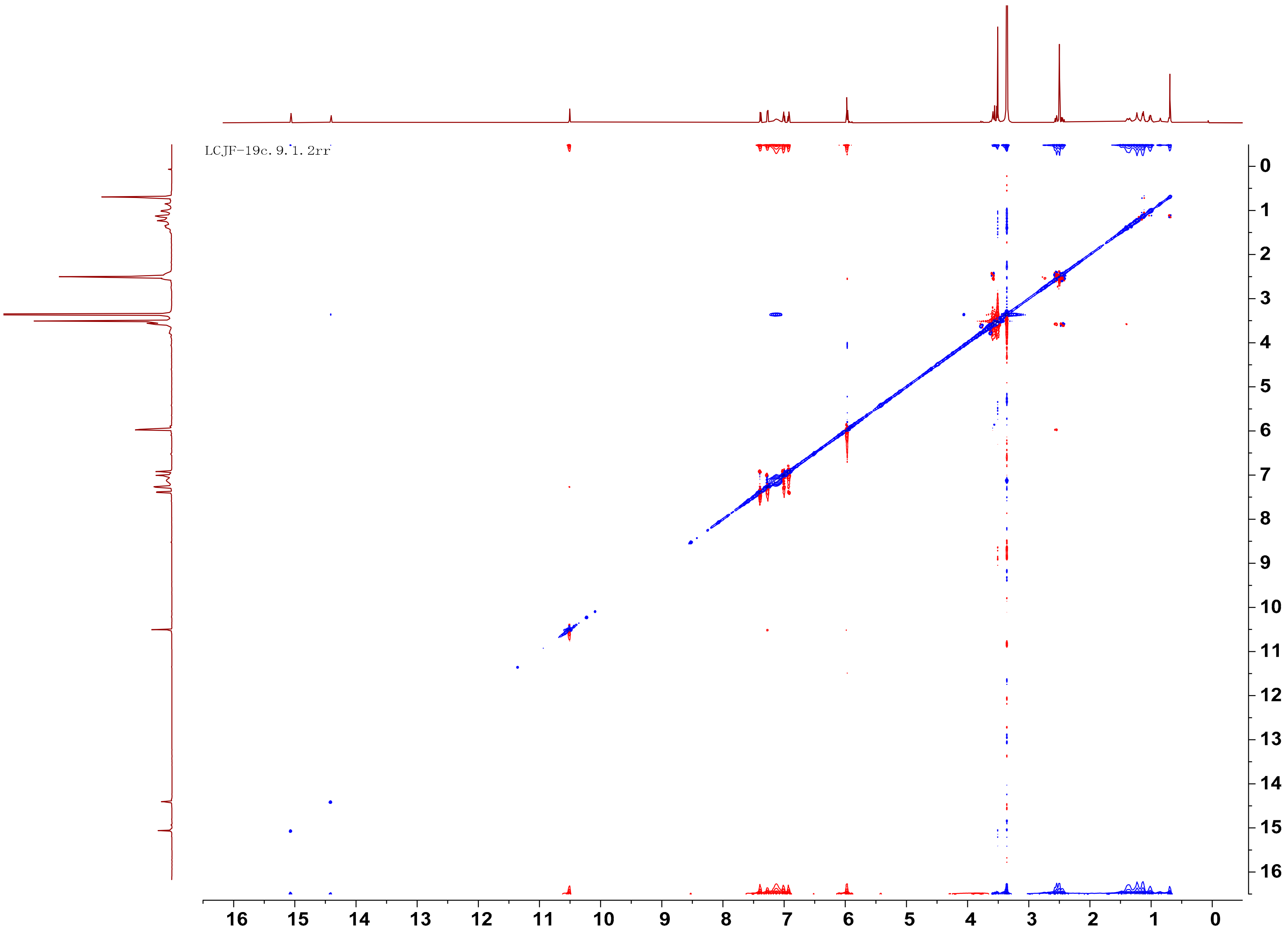

Figure S7. Entire and partly (+)-HRESIMS spectrum of **1**.

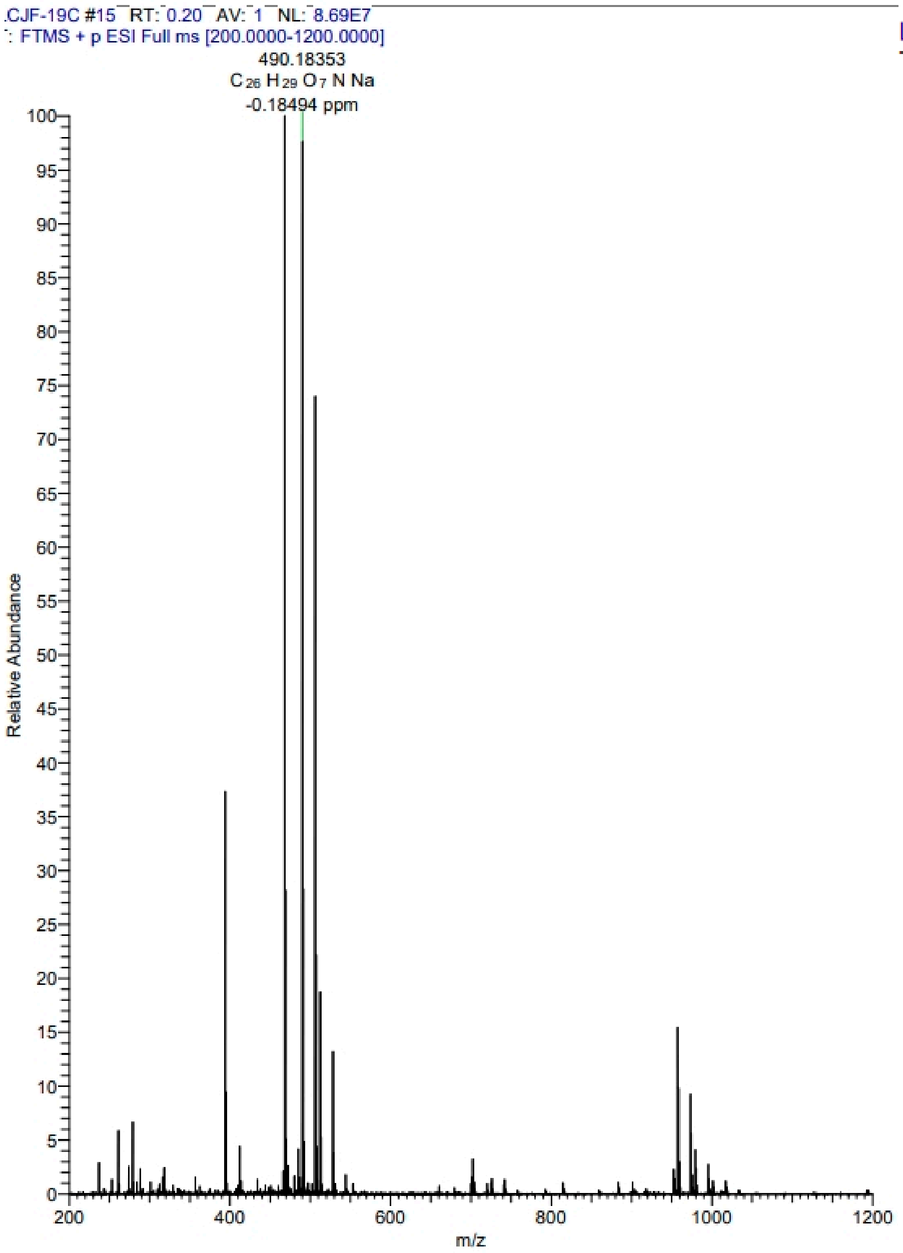

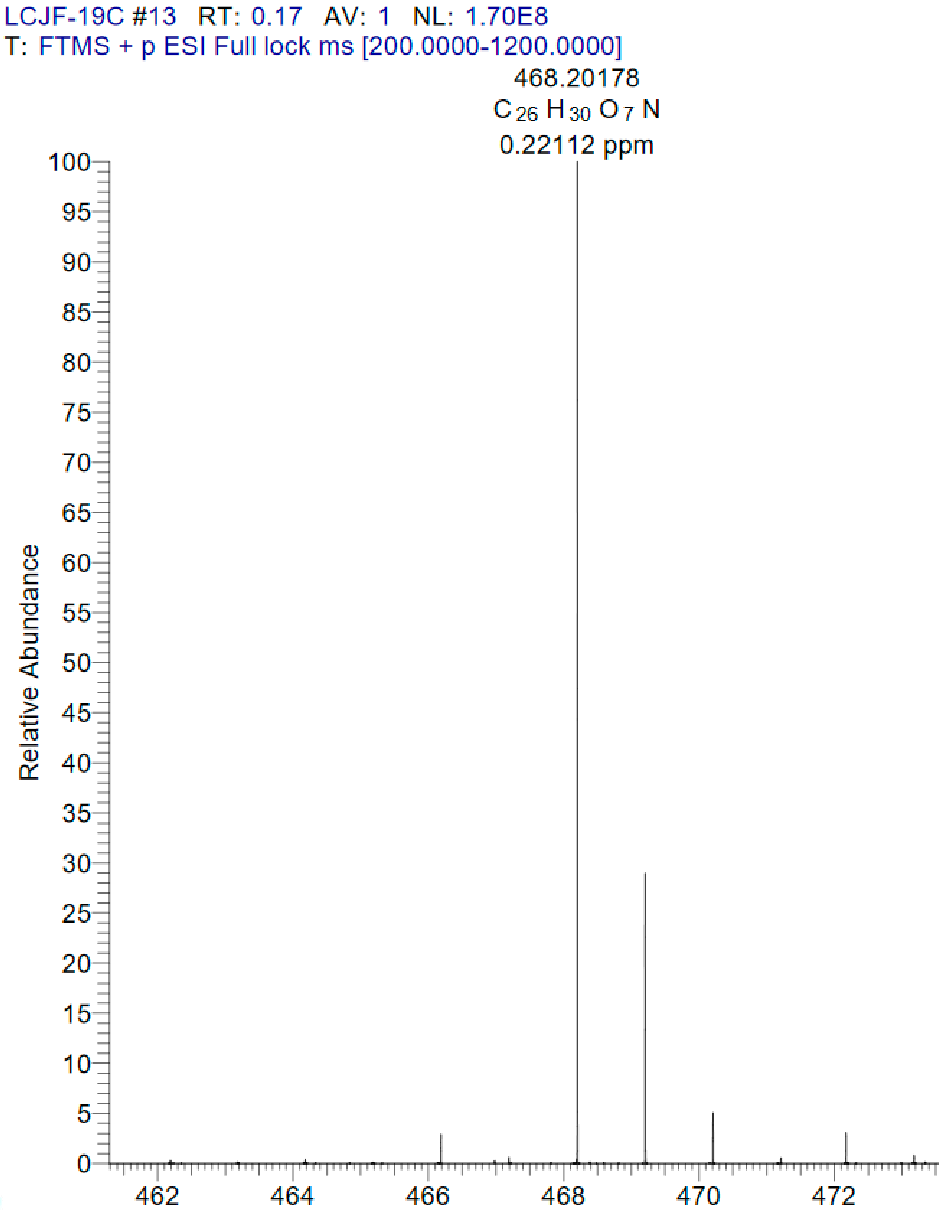

Figure S8. CD and UV spectra of compound **1**.

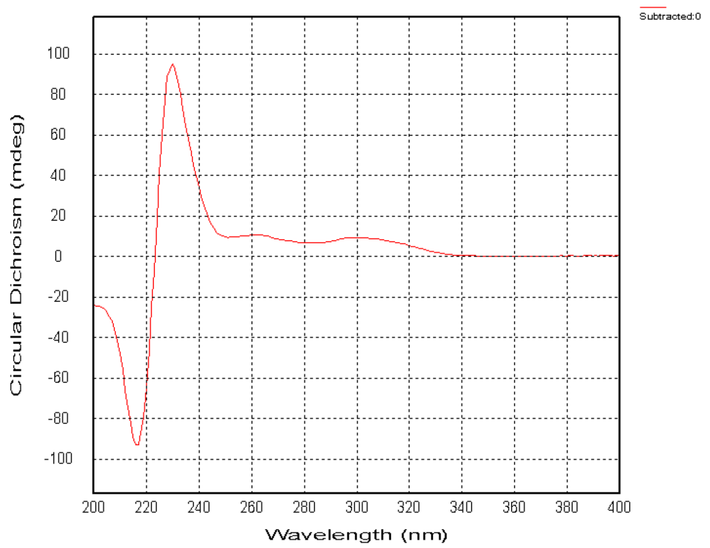

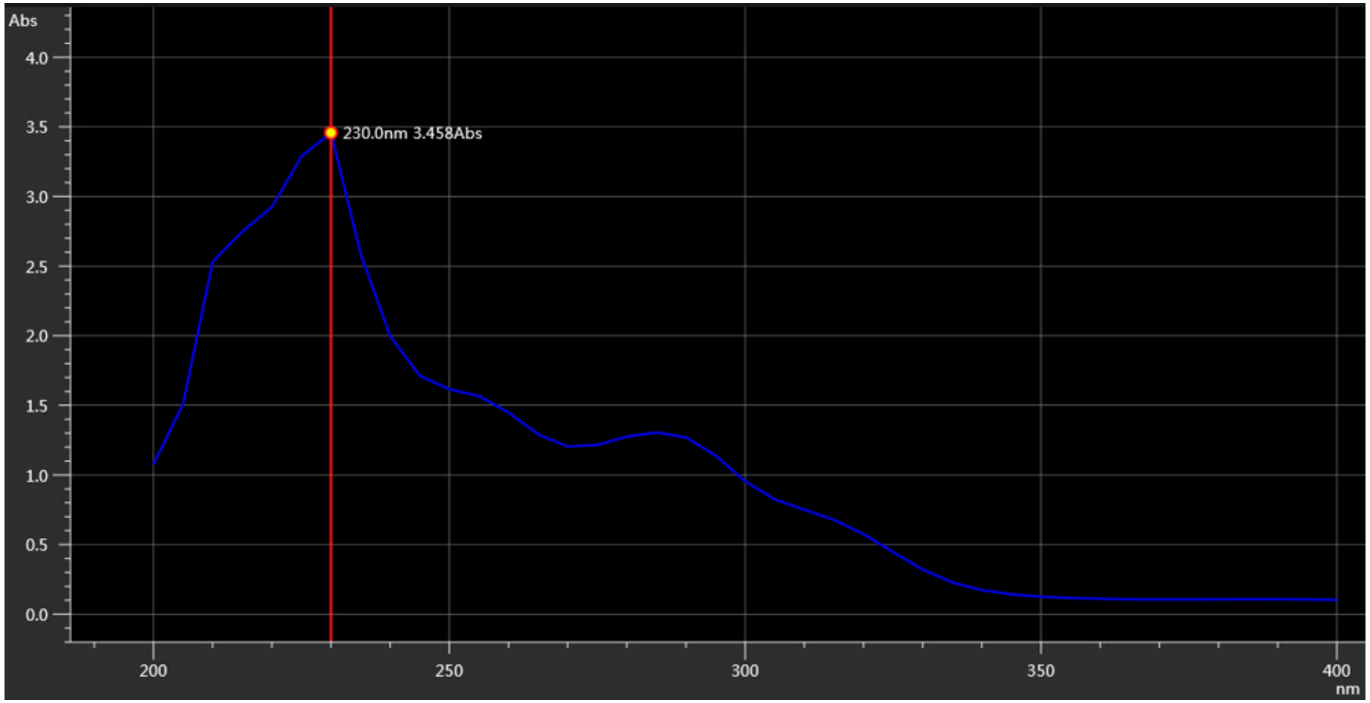

Figure S9. ^{1}H NMR spectrum of **2** (600 MHz, DMSO-d_6).

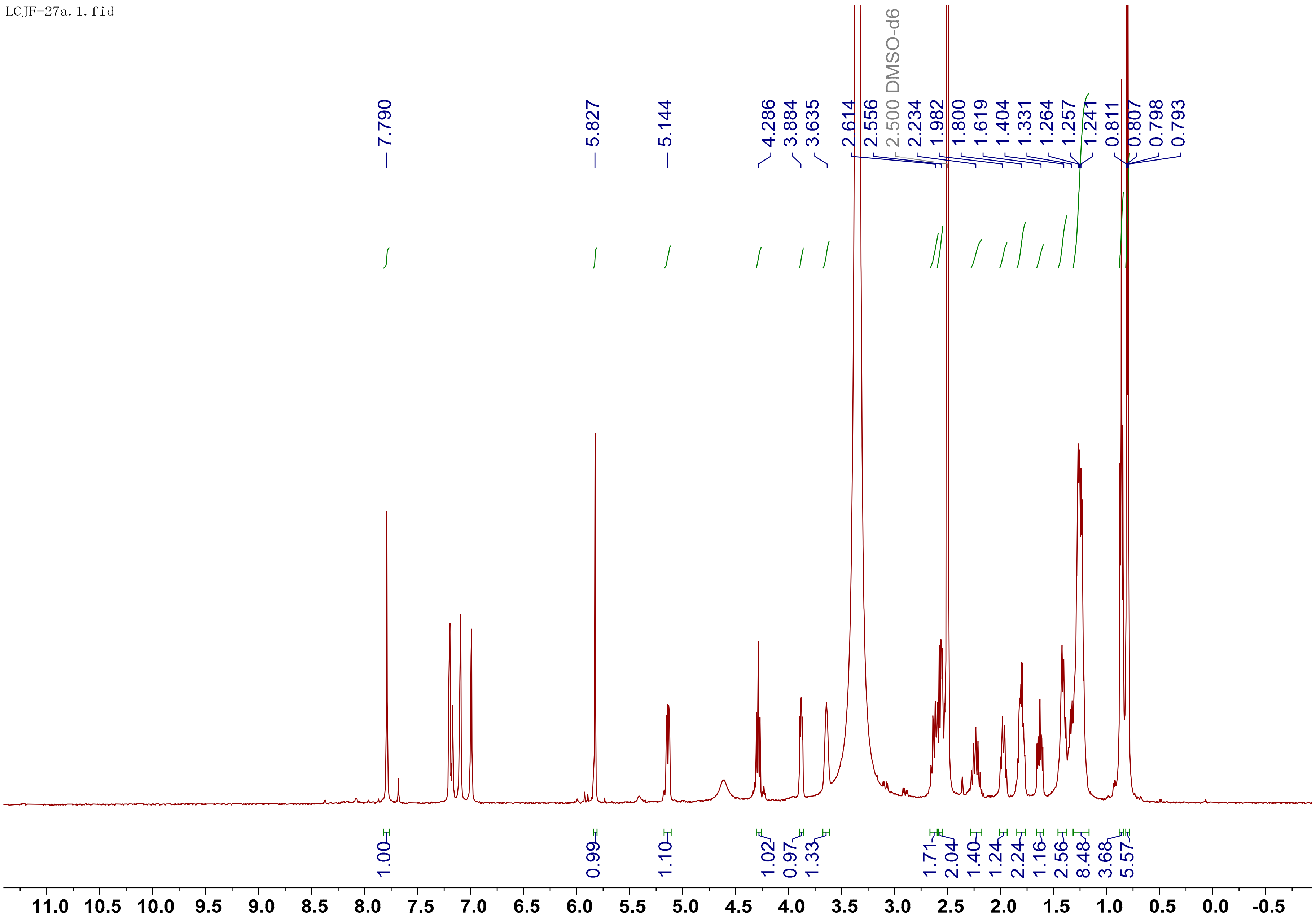

Figure S10. ^{13}C NMR spectrum of **2** (150 MHz, DMSO-d_6).

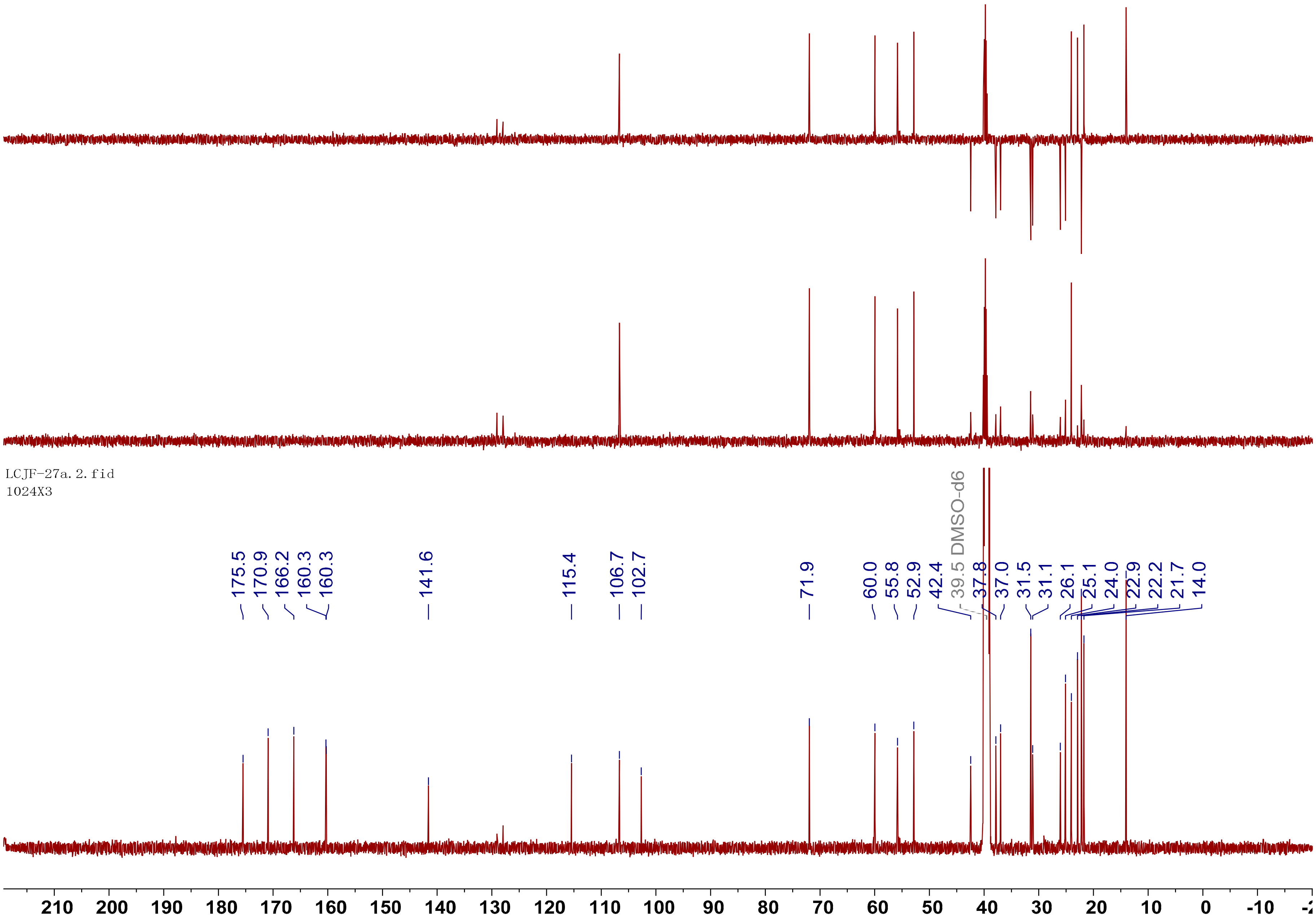

Figure S11. HSQC spectrum of 2.

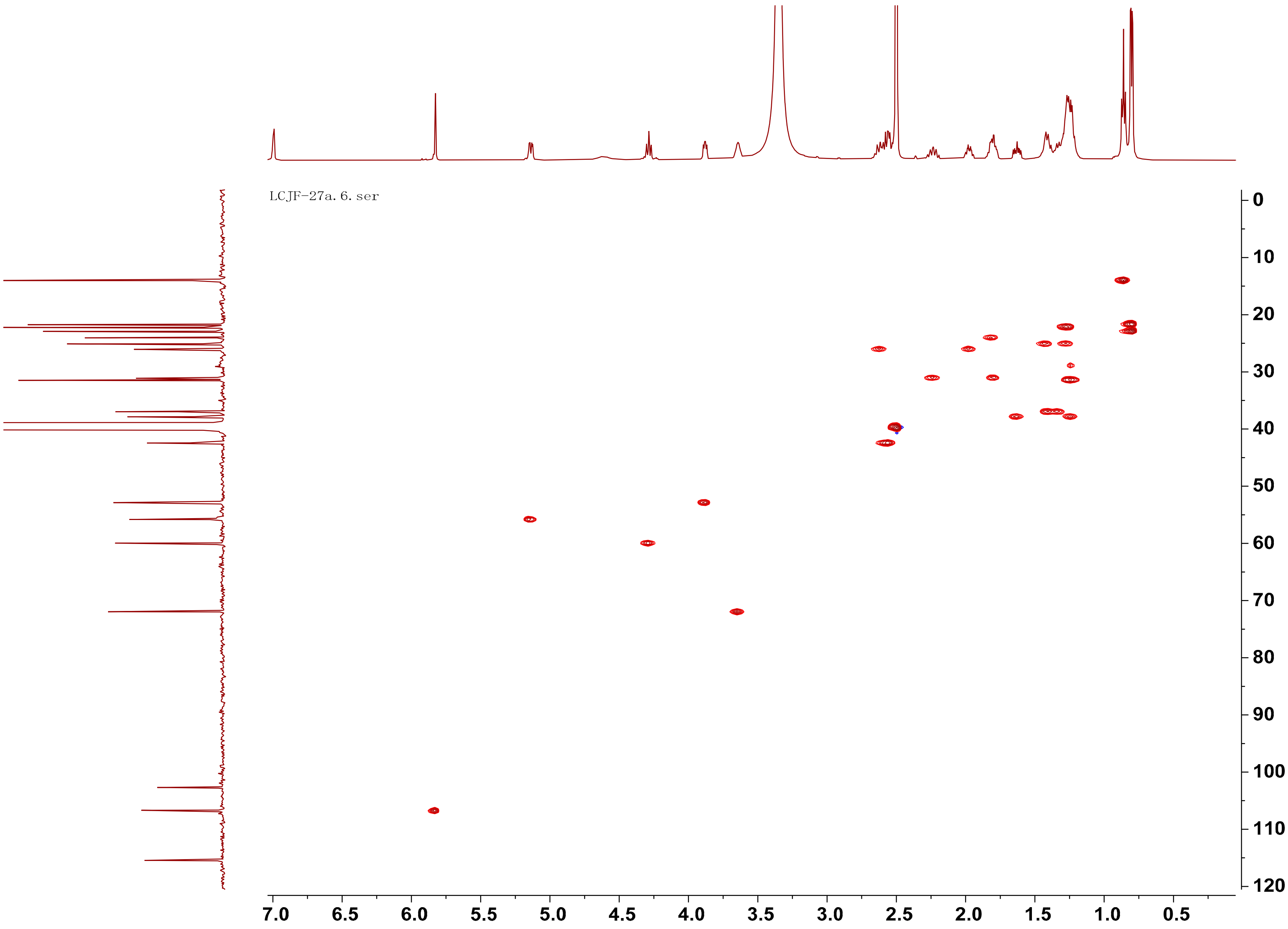

Figure S12. ^{1}H-^{1}H COSY spectrum of 2.

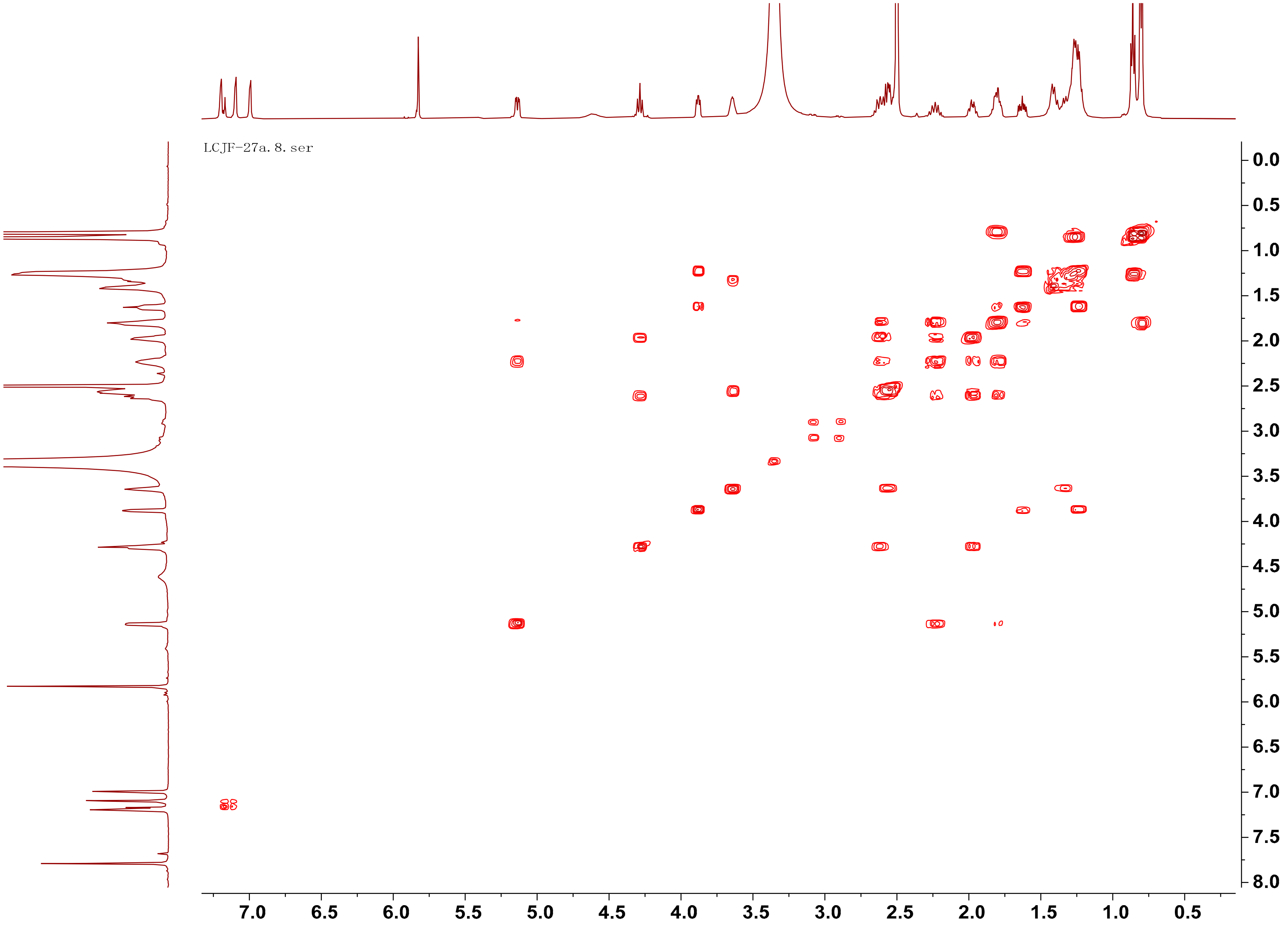

Figure S13. HMBC spectrum of **2**.

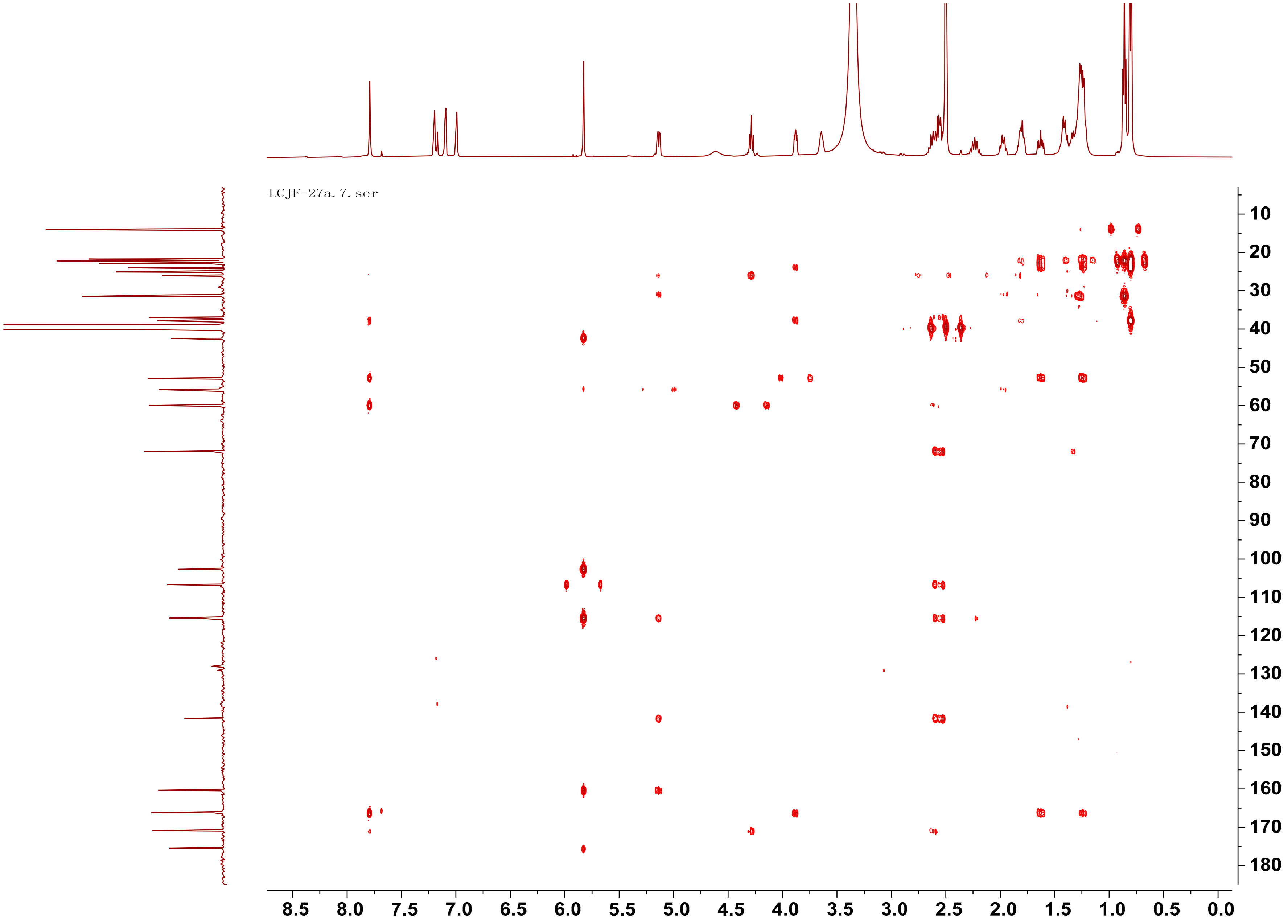

Figure S14. ROESY spectrum of **2**.

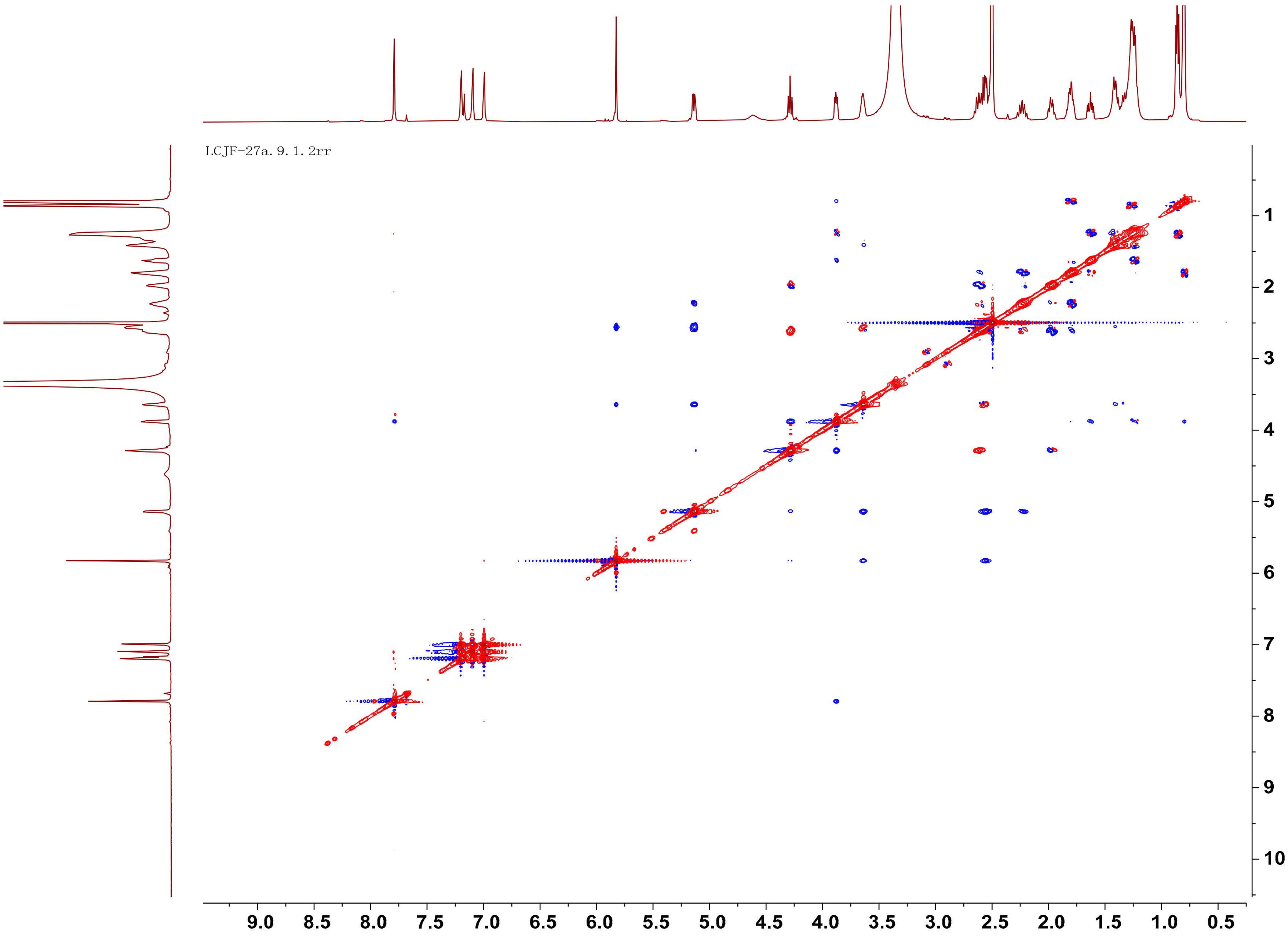

Figure S15. Enlarged ROESY spectrum of **2**.

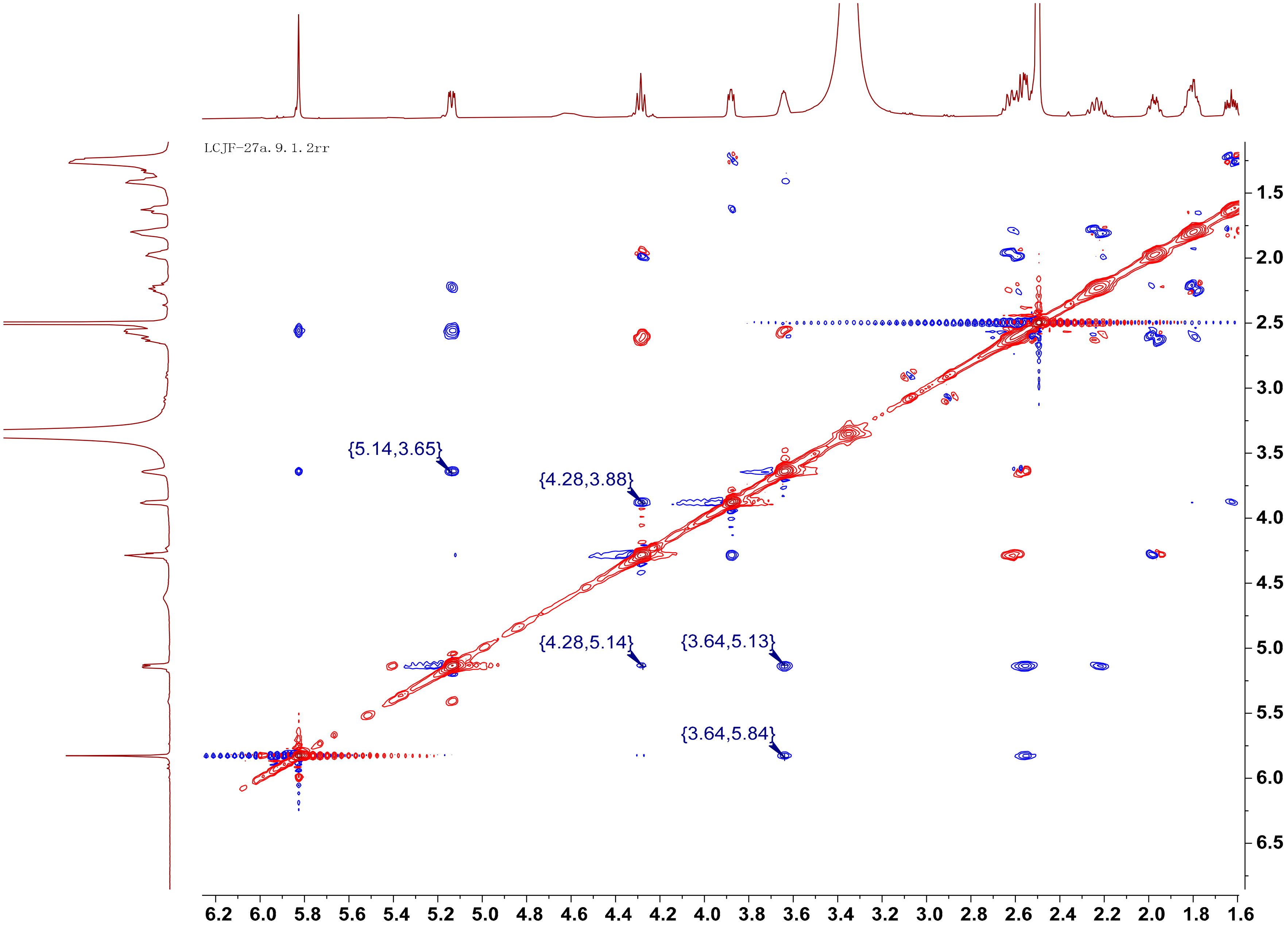

Figure S16. Entire and partly (+)-HRESIMS spectrum of **2**.

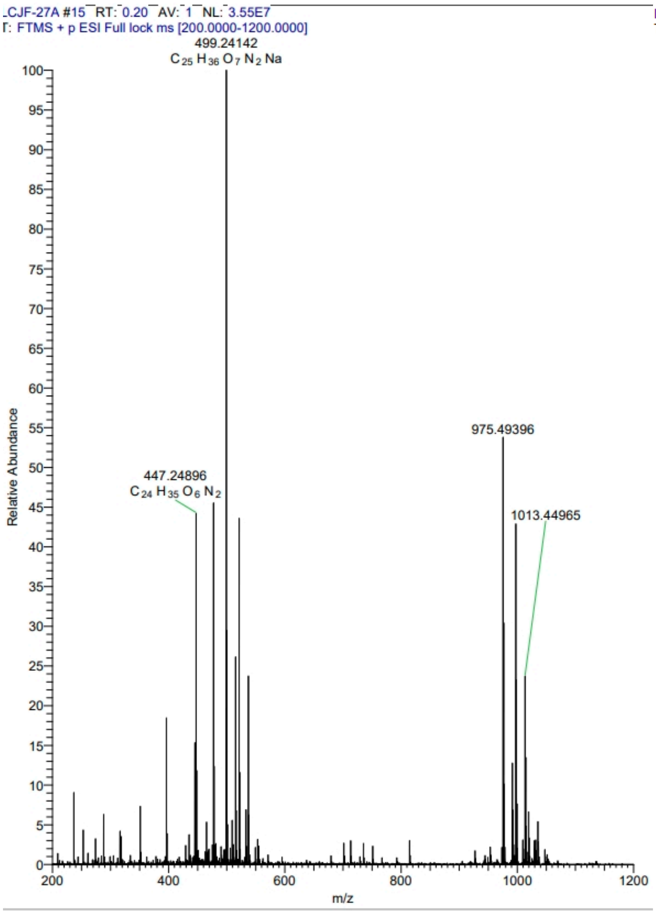

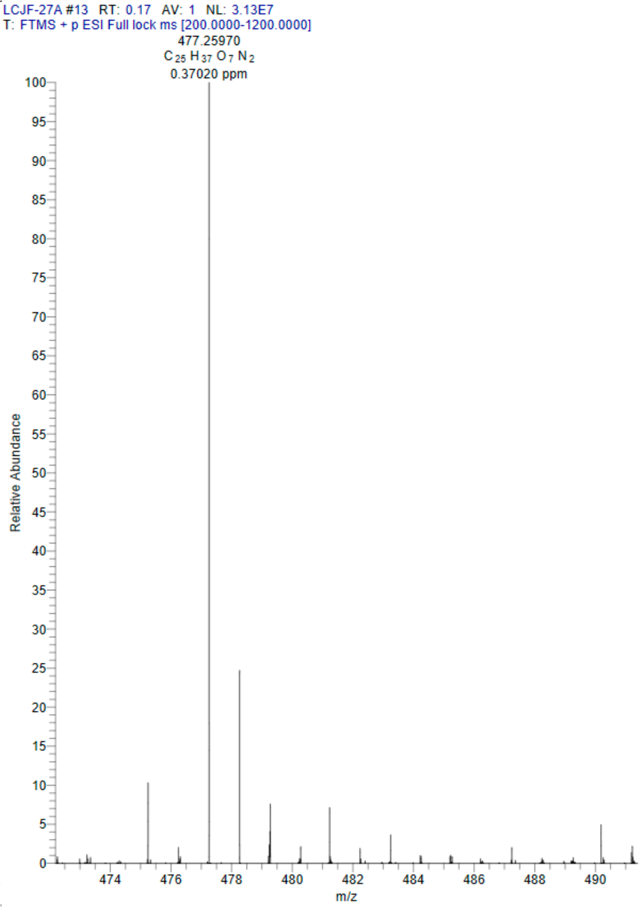

Figure S17. CD and UV spectra of compound **2**.

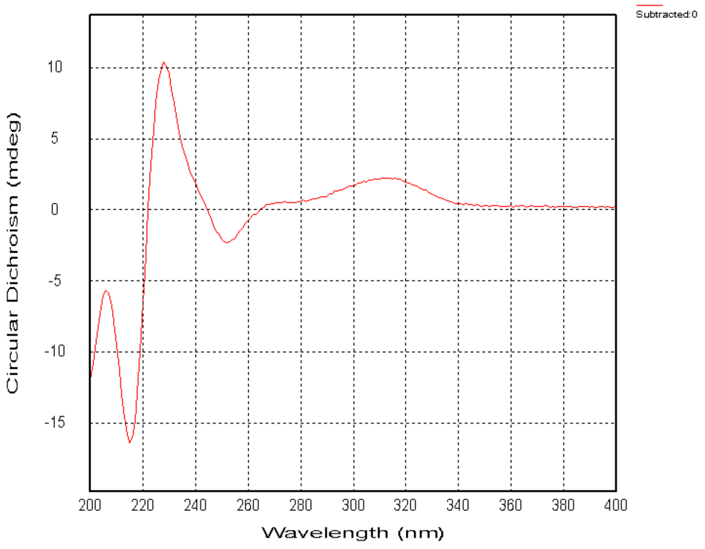

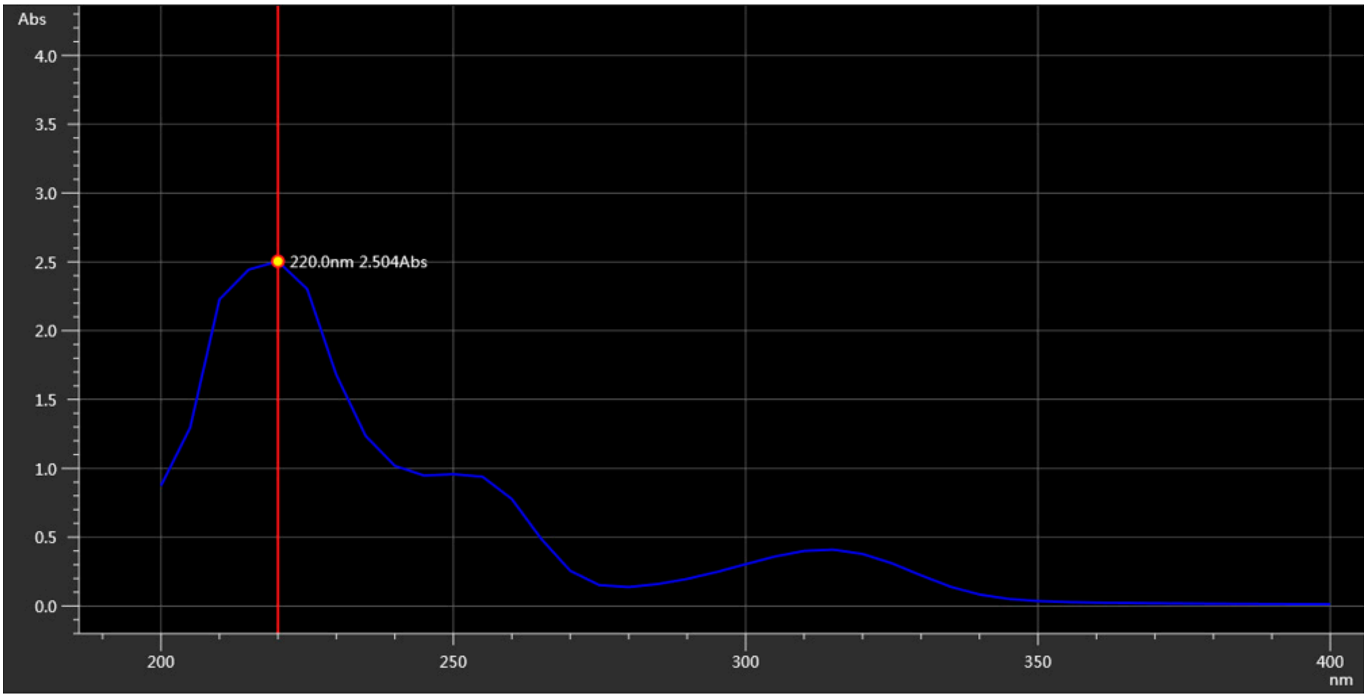

Figure S18. Acetylcholinesterase Inhibitory activity of 1.

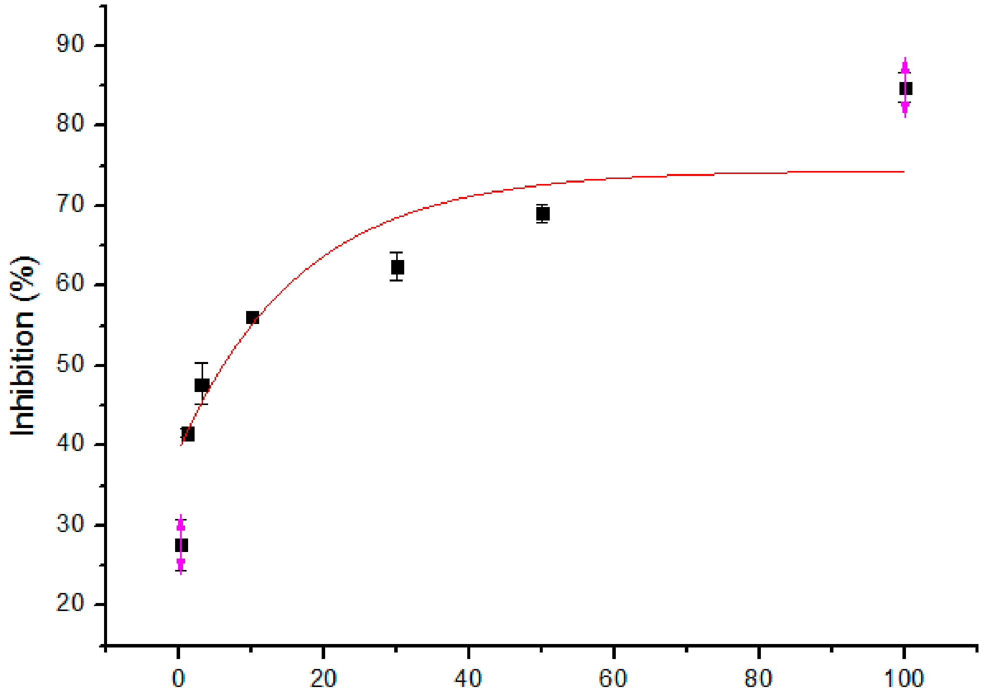

Figure S19. LC-MS analysis of crude extract (TIC).

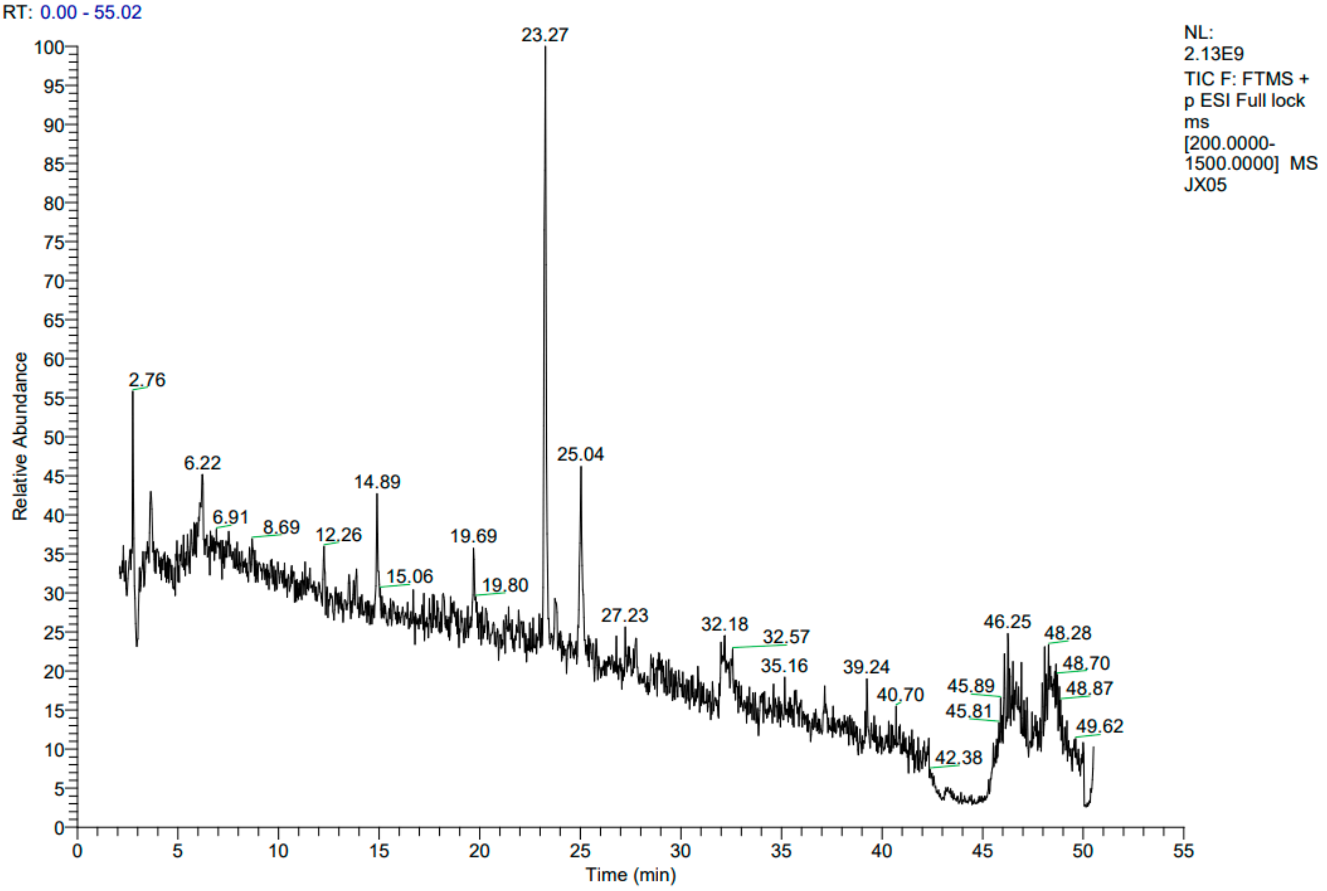

S2. Computational Details

S2.1 ECD calculation details of compound 1.

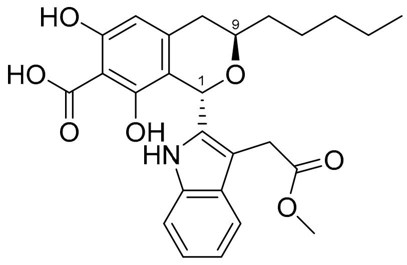

1*S*,9*R*-**1a**

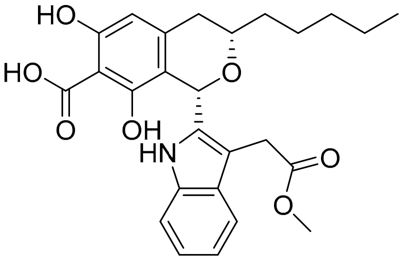

1*S*,9*S*-**1b**

Conformation searches based on molecular mechanics with MMFF94s force field were performed for **1a** and **1b** gave 17 and 23 conformers with populations higher than 1%, respectively. All these conformers were further optimized by the density functional theory method at the B3LYP/6-31G(d) level in Gaussian 16 program package, led to both 14 [**1aa–1an**] and 14 [**1ba–1bn**] conformers within a 3 kcal/mol energy threshold from global minimum, respectively. These predominant conformers were subjected to theoretical calculation of ECD using time-dependent density functional theory (TDDFT) at b3lyp/6-31g(d,p) level in MeOH with IEFPCM model. The calculated ECD curves for (1*S*,9*R*)-**1a** and (1*S*,9*S*)-**1b** were generated using SpecDis 1.71 with σ = 0.22 eV, and UV shift +12 nm, and σ = 0.16 eV, and UV shift -30 nm respectively

Table S1. Energy analysis for conformers of **1aa-1aq** and **1ba-1bw** at B3LYP/6-31G(d) level.

Species	Gibbs free energy	*ΔE* (kcal/mol)	P_E%
**1aa**	-1589.152166	0.00	21.4
**1ab**	-1589.150637	0.96	4.2
**1ac**	-1589.150446	1.08	3.5
**1ad**	-1589.15021	1.23	2.7
**1ae**	-1589.150203	1.23	2.7
**1af**	-1589.152086	0.05	19.7
**1ag**	-1589.151944	0.14	16.9
**1ah**	-1589.150308	1.17	3.0
**1ai**	-1589.151409	0.48	9.6
**1aj**	-1589.150234	1.21	2.8
**1ak**	-1589.149687	1.56	1.6
**1al**	-1589.150492	1.05	3.6
**1am**	-1589.149301	1.80	1.0
**1an**	-1589.151141	0.64	7.2
**1ba**	-1589.15104	0.00	21.1
**1bb**	-1589.150214	0.52	8.8
**1bc**	-1589.150027	0.64	7.2
**1bd**	-1589.149058	1.24	2.6
**1be**	-1589.149475	0.98	4.0
**1bf**	-1589.149416	1.02	3.8
**1bg**	-1589.148629	1.51	1.6
**1bh**	-1589.149791	0.78	5.6
**1bi**	-1589.150866	0.11	17.5
**1bj**	-1589.149236	1.13	3.1
**1bk**	-1589.150865	0.11	17.5
**1bl**	-1589.149296	1.09	3.3
**1bm**	-1589.148803	1.40	2.0
**1bn**	-1589.1487	1.47	1.8

S2.2 NMR calculation details of compound 1.

The optimized conformers **1aa-1an** and **1ba-1bn** were further subjected to NMR calculation by using the Gauge-Independent Atomic Orbital (GIAO) method at mPW1PW91/6-31G+(d,p) level with PCM model in methanol in Gaussian 16 software package. The calculated NMR data of these conformers were averaged according to the Boltzmann distribution theory and their relative Gibbs free energy. The DP4+ analysis of the results were conducted on the EXCEL file provided by the publication on *J. Org. Chem.*, 2015, 80 (24), pp 12526–12534.

Table S2. NMR calculation results of 1a at mPW1PW91/6-31+G(d,p)// B3LYP/6-31G(d) level of theory.

Shielding tensors															
No.	**1aa**	**1ab**	**1ac**	**1ad**	**1ae**	**1af**	**1ag**	**1ah**	**1ai**	**1aj**	**1ak**	**1al**	**1am**	**1an**	Scaled
1	126.8832	125.6641	125.5616	125.1037	125.1034	127.416	126.9796	125.6641	125.5091	127.8778	125.6624	125.442	128.2819	125.2923	127.0
2	85.8437	83.7736	84.2939	85.0639	85.0655	85.2703	84.3965	83.7736	84.323	85.03	84.7401	85.2143	85.5254	84.5924	85.0
3	41.5878	38.6002	42.5245	41.735	41.7312	37.8475	38.5567	38.6002	42.5198	42.3497	42.6511	38.124	38.077	38.5626	40.2
4	100.8429	101.4803	101.3118	99.3837	99.3855	100.9141	100.6697	101.4803	101.1982	100.913	101.1871	99.3515	101.0388	99.002	100.8
5	35.5625	40.0503	35.9916	36.4099	36.4122	39.2109	39.9053	40.0503	36.1897	35.426	35.6739	40.7549	39.3518	40.6898	37.5
6	90.0637	90.6177	90.1763	90.5391	90.5392	90.4417	89.9201	90.6177	90.0931	90.0667	90.1881	90.5107	90.924	90.4496	90.2
7	47.7452	46.6752	46.8439	48.6913	48.6956	47.4621	47.3043	46.6752	46.4674	48.3132	48.3355	49.6048	48.591	49.2864	47.8
8	158.4028	158.3308	158.3812	158.5528	158.5512	158.4022	159.5229	158.3308	158.4169	158.5219	158.3703	158.7345	158.1191	158.5338	158.6
9	130.2806	129.972	129.9696	130.3747	130.3768	130.1763	125.8333	129.972	130.2871	126.4287	130.5999	130.3613	130.1317	130.1351	128.9
10	159.1235	158.1614	158.1446	158.897	158.8997	159.203	158.4128	158.1614	158.6435	158.0671	158.07	158.816	158.7545	158.8482	158.6
11	168.741	168.2591	168.2857	168.5279	168.5285	168.7321	167.5742	168.2591	171.2273	166.4324	170.993	168.3661	168.985	171.5841	168.3
12	164.6634	164.4296	164.4382	164.8395	164.8417	164.6729	160.9156	164.4296	166.447	161.0961	165.9025	164.7864	163.9576	166.6094	163.5
13	169.5043	169.373	169.375	169.4623	169.4624	169.3719	169.4494	169.373	171.1886	168.9739	170.6789	169.4761	170.2092	171.4652	169.5
14	180.7573	180.6365	180.6404	180.8513	180.8517	180.6854	180.3012	180.6365	182.5062	180.3098	182.6606	180.8036	180.6613	182.5786	180.7
15	29.0499	28.7211	29.4581	28.2177	28.2211	28.6431	28.8098	28.7211	29.3427	29.1556	29.4413	28.8916	29.0168	28.9314	28.9
16	60.5053	60.587	60.5688	61.3847	61.384	60.8578	60.9247	60.587	60.3184	60.6786	61.9252	61.9629	61.4624	61.3751	60.8
17	89.1964	89.652	89.5808	89.1518	89.1511	89.0445	89.0952	89.652	89.7274	89.4889	88.4026	87.7411	88.756	87.8416	89.1
18	69.5019	69.0177	68.9442	69.9911	69.9904	69.4608	69.7335	69.0177	68.8772	69.0299	69.9008	68.5285	69.392	69.1756	69.5
19	79.6348	81.2082	81.1949	78.6836	78.6831	78.9531	79.6406	81.2082	81.2145	79.0489	79.2804	79.3292	77.1329	78.9074	79.4
20	79.223	79.7448	79.7362	79.9313	79.9326	79.2653	79.3488	79.7448	79.917	79.9627	79.5875	79.8844	79.845	79.9915	79.5
21	76.9442	76.565	76.5764	76.3437	76.3445	76.8823	77.2364	76.565	76.8339	77.28	75.7939	76.2702	76.9439	76.0559	76.9
22	87.6083	87.3903	87.3789	87.4235	87.4254	87.6267	87.7311	87.3903	87.6044	87.4778	87.4424	87.5858	87.8053	88.1825	87.6
23	65.3871	64.225	64.2121	64.27	64.2715	65.2444	64.6918	64.225	64.5271	65.451	63.8351	64.025	64.6967	63.5577	64.9
24	163.0564	165.4942	165.493	164.2044	164.2006	161.9363	162.4256	165.4942	165.5909	163.805	163.6215	163.8661	162.6103	163.4048	163.0
25	24.0321	23.9962	24.0333	25.1279	25.1296	24.7705	22.8905	23.9962	23.9719	24.9704	23.4537	25.4571	24.0437	25.351	24.1
25-OCH_3	143.6005	143.7833	143.7367	143.858	143.859	143.6293	143.4325	143.7833	143.6888	143.3343	143.4974	143.8565	143.1794	143.8744	143.6
P	0.21434	0.04244	0.03467	0.027	0.0268	0.19693	0.16943	0.02995	0.09614	0.02770	0.01552	0.03640	0.01031	0.07238	

Table S3. NMR calculation results of 1b at mPW1PW91/6-31+G(d,p)// B3LYP/6-31G(d) level of theory.

Shielding tensors														
No.	**1ba**	**1bb**	**1bc**	**1bd**	**1be**	**1bf**	**1bg**	**1bh**	**1bi**	**1bj**	**1bk**	**1bl**	**1bm**	**1bn**	Scaled
1	124.5793	121.8837	121.939	122.0249	121.9604	122.5838	122.499	123.0789	123.9558	122.4635	123.9707	122.4781	123.1236	122.444	123.4
2	82.0708	82.4004	82.3966	82.2552	82.6088	82.2299	82.7486	82.4153	81.1381	81.8559	81.455	82.5063	82.1031	82.9452	81.9
3	42.1014	40.556	41.1884	40.5479	38.0068	37.7205	41.8071	42.7619	42.6968	42.8224	42.1996	37.4814	38.4254	41.2154	41.5
4	100.3869	99.884	99.5479	99.9843	100.5981	101.0042	100.834	100.8622	99.0836	100.463	100.2793	100.9164	101.2674	100.8081	100.1
5	36.4578	36.5339	36.3774	36.4888	40.908	39.7694	35.8021	35.994	36.9731	35.9323	36.1451	40.1423	39.7533	36.2317	36.9
6	90.6436	90.9763	91.2593	91.2177	90.9397	91.1711	90.7711	90.6176	91.0876	90.5625	90.8879	91.1951	91.1737	90.7887	90.9
7	48.1158	50.2429	50.7253	50.3448	49.75	49.5733	49.2492	47.2516	49.6833	48.393	50.3972	47.9485	47.2948	47.8011	49.3
8	157.0111	158.4597	158.6704	158.4687	157.9403	158.3842	158.4913	158.3381	157.5335	158.0489	158.2973	158.289	158.2063	158.2854	157.9
9	120.9826	120.7899	120.9165	125.8065	121.0489	120.524	120.4037	120.2668	120.7788	120.5839	121.1956	124.0846	120.1719	124.1918	121.2
10	157.5777	157.5369	157.3425	162.9894	157.5864	157.7449	157.7213	160.7788	159.7828	157.5568	159.571	160.0277	162.1991	159.8697	158.8
11	168.8821	169.0632	169.0036	169.9791	169.0143	169.1946	169.1655	168.0191	167.2657	169.1182	167.8051	171.1528	172.8576	171.1708	168.6
12	163.2178	163.2405	163.1946	166.5228	163.209	163.1556	163.1722	162.4109	162.431	163.1724	162.5917	165.3223	164.6027	165.324	163.1
13	170.5612	170.5982	170.6263	172.6154	170.6243	170.6005	170.6284	171.7726	171.778	170.6918	171.7853	170.3342	174.9053	170.337	171.2
14	182.22	182.3759	182.2385	180.1897	182.3465	182.4123	182.4016	180.0635	180.086	182.2232	180.1369	182.2626	183.6234	182.2488	181.4
15	29.4731	29.0586	29.2123	29.4047	28.7909	28.8233	29.5103	29.4892	29.06	29.3808	28.7332	28.609	28.8507	29.2193	29.1
16	60.9314	58.1437	58.3376	58.1641	59.4389	59.1707	58.9482	58.8334	60.2288	59.0859	60.2255	59.4971	58.6682	59.7389	59.7
17	88.1554	92.435	92.2827	92.1086	90.3191	90.4434	90.6271	91.5149	89.6507	90.5428	89.74	91.0164	90.8642	91.7794	90.2
18	70.051	69.0722	69.0246	68.4387	67.8541	68.2745	68.6489	69.254	69.7346	68.6459	69.9014	68.7749	69.1343	68.6491	69.4
19	78.8712	80.5856	80.7754	80.7981	80.7249	79.4722	78.7527	80.0471	79.3911	78.4737	79.5304	80.1992	80.1387	80.0736	79.7
20	79.3549	79.8041	79.6084	79.7629	79.5654	79.4968	79.521	79.7719	79.2809	79.4963	79.7701	79.6366	79.6602	79.8533	79.6
21	76.6469	77.4091	77.429	77.5714	77.4825	77.0348	76.946	77.4744	76.7059	76.9133	77.0463	77.2734	77.3448	77.4255	77.0
22	87.7223	87.5273	87.3488	87.3474	87.2644	87.6081	87.7863	87.7286	87.8091	87.5953	87.6998	87.5116	87.6759	87.443	87.6
23	63.5515	64.921	64.9208	64.9937	64.5736	64.4378	64.6048	64.4807	63.8218	64.5968	64.0294	64.4103	64.3057	64.8886	64.2
24	161.1868	165.3498	165.2293	164.984	164.9954	163.6836	163.4805	162.5846	161.5419	163.2013	161.4656	162.5525	162.5404	162.6755	162.6
25	24.808	25.2453	25.3802	25.2342	24.0949	23.7632	23.8046	24.9691	21.9752	23.7472	21.9174	24.7287	24.8249	24.8401	23.8
25-OCH_3	143.5155	143.8133	143.8645	143.876	143.5254	143.7177	143.7324	143.9548	143.2372	143.7074	143.22	143.9413	143.917	143.9693	143.5
P	0.21093	0.08794	0.07214	0.02585	0.0402	0.03777	0.01641	0.05618	0.17543	0.03121	0.17524	0.03326	0.01973	0.01769	

Table S4. DP4+ analysis details of 1.

PCM-mPW1PW91/6-31G+(d,p)/ Shielding Tensors				
Nuclei	Sp2?	experimental	1a	1b
1C	x	104.2	90.2	90.9
2C	x	161.1	37.5	36.9
3C	x	102.3	100.8	100.1
4C	x	159	40.2	41.5
5C	x	109.9	85.0	81.9
6C	x	138.3	47.8	49.3
9C	x	175.5	28.9	29.1
10C		34.1	158.6	157.9
11C		67.5	128.9	121.2
13C	x	128.4	69.5	69.4
14C	x	134.9	64.9	64.2
15C	x	111.3	87.58	87.64
16C	x	120.7	76.87	77.03
17C	x	118.2	79.45	79.55
18C	x	118.3	79.41	79.65
19C		13.9	180.70	181.36
20C		22.1	169.49	171.20
21C		31.1	163.50	163.14
22C		24.3	168.31	168.63
23C		35.4	158.63	158.83
24C		66.7	126.99	123.37
27C	x	136.7	60.85	59.73
28C	x	105.1	89.14	90.16
29C		29.2	162.98	162.58
30C	x	171.9	24.13	23.78
33C		51.2	143.56	143.55
35H	x	5.96	24.79	24.91
38H		2.56	28.88	28.94
39H		2.44	28.92	28.48
40H		3.58	27.74	27.78
41H	x	7.27	23.94	23.86
42H	x	7	24.02	24.02
43H	x	6.92	24.06	24.07
44H	x	7.38	23.45	23.49
45H		0.69	30.98	30.70
48H		1.14	30.41	30.19
50H		1	30.84	30.24
52H		1.16	30.39	30.31
53H		1.22	30.09	29.74
54H		1.39	30.18	30.03
55H		1.35	29.84	29.95
56H		5.97	24.98	25.33
58H		3.51	27.65	27.63
59H		3.56	27.59	27.63
60H		3.5	27.84	27.86

S2.2 NMR calculation details of compound 2.

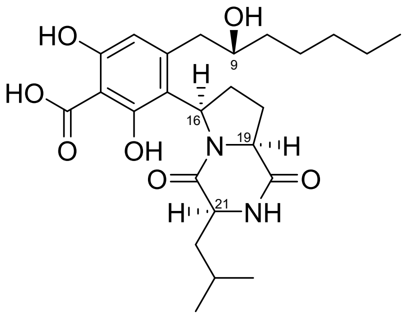

(9*R*,16*R*,19*S*,21*S*)-2a
2a

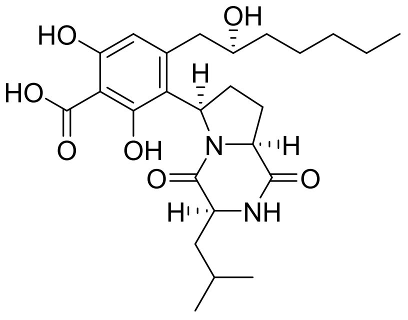

(9*S*,16*R*,19*S*,21*S*)-2b
2b

Conformation searches based on molecular mechanics with MMFF94s force field were performed for **2a** and **2b** gave 14 and 24 conformers with populations higher than 1%, respectively. All these conformers were further optimized by the density functional theory method at the B3LYP/6-31G(d) level in Gaussian 16 program package, led to both 8 [**1aa–1ah**] and 9 [**1ba–1bi**] conformers within a 3 kcal/mol energy threshold from global minimum, respectively. These predominant conformers were subjected to NMR calculation by using the Gauge-Independent Atomic Orbital (GIAO) method at mPW1PW91/6-31G+(d,p) level with PCM model in methanol in Gaussian 16 software package. The calculated NMR data of these conformers were averaged according to the Boltzmann distribution theory and their relative Gibbs free energy. The DP4+ analysis of the results were conducted on the EXCEL file provided by the publication on *J. Org. Chem.*, 2015, 80 (24), pp 12526–12534.

Table S5. Energy analysis for conformers of 2aa-2ah and 2ba-2bi at B3LYP/6-31G(d) level.

Species	Gibbs free energy	*ΔE* (kcal/mol)	P_E%
**2aa**	-1609.958315	0.00	54.0
**2ab**	-1609.957518	0.50	23.2
**2ac**	-1609.955114	2.01	1.8
**2ad**	-1609.954712	2.26	1.2
**2ae**	-1609.955493	1.77	2.7
**2af**	-1609.95557	1.72	3.0
**2ag**	-1609.95618	1.34	5.6
**2ah**	-1609.956557	1.10	8.4
**2ba**	-1609.956439	0.00	41.1
**2bb**	-1609.953328	1.95	1.5
**2bc**	-1609.955839	0.38	21.8
**2bd**	-1609.952984	2.17	1.1
**2be**	-1609.953925	1.58	2.9
**2bf**	-1609.954359	1.31	4.5
**2bg**	-1609.954664	1.11	6.3
**2bh**	-1609.955006	0.90	9.0
**2bi**	-1609.955266	0.74	11.9

Table S6. NMR calculation results of 2a at mPW1PW91/6-31+G(d,p)// B3LYP/6-31G(d) level of theory.

Shielding tensors									
GV No.	**2aa**	**2ab**	**2ac**	**2ad**	**2ae**	**2af**	**2ag**	**2ah**	Scaled
2	77.1758	77.4183	77.331	77.4032	76.942	78.0212	77.6376	77.5305	77.3
3	37.019	41.0831	36.4036	40.4143	36.6025	40.7613	36.7618	40.9981	38.4
4	100.7545	100.5636	101.1082	101.0527	101.1914	100.8267	100.9868	100.5745	100.7
5	40.4777	36.4491	39.1302	34.9923	39.0351	36.0755	39.9698	35.9965	38.9
6	88.1784	87.6688	88.2365	87.7602	88.3412	87.8613	88.3284	88.1258	88.1
7	44.1422	43.8848	44.4065	43.828	43.9454	43.3788	43.6218	44.0683	44.0
8	148.3905	148.3752	148.5696	148.6312	148.58	151.4786	151.4997	148.2681	148.7
9	119.8014	119.7658	120.1777	119.8916	119.9445	121.1178	121.1336	123.6569	120.2
10	154.9655	154.9362	155.0995	155.0861	155.0922	155.125	155.2189	156.2098	155.1
11	165.485	165.4832	165.4912	165.4902	165.4723	171.4382	171.4223	168.0566	166.2
12	161.1703	161.1734	161.1919	161.1863	161.1835	160.6436	160.6373	163.9444	161.4
13	169.4015	169.4026	169.4189	169.4158	169.4093	169.304	169.3083	169.095	169.4
14	180.1615	180.1612	180.1212	180.1632	180.1695	180.2143	180.2208	180.1591	180.2
15	28.6911	29.3744	28.588	29.2961	28.5515	29.3655	28.6395	29.4183	28.9
16	135.1853	134.9985	135.2369	134.8745	135.1423	134.8112	135.0499	134.8724	135.1
17	162.8766	162.7231	162.8261	162.8615	162.8545	162.6386	162.7123	162.7806	162.8
18	164.6464	164.897	165.1412	165.026	164.8571	165.0434	164.7425	164.914	164.8
19	133.1285	133.1477	132.9392	133.0004	132.8588	133.3529	133.387	133.2314	133.2
20	29.4191	29.1778	29.5808	30.3279	30.4909	29.1275	29.3896	29.1093	29.4
21	140.257	140.2582	138.8161	137.598	137.6612	140.3169	140.3008	140.1804	140.1
22	32.4471	32.2724	32.1643	32.7244	32.7724	32.3405	32.4429	32.2861	32.4
23	156.8889	157.1218	160.3054	156.8912	156.8145	156.9013	156.5927	157.1432	157.0
24	168.8416	168.7594	168.6596	168.5962	168.5358	168.8253	168.837	168.6665	168.8
25	171.9618	171.9879	175.8089	171.3682	171.3187	171.9124	171.9315	171.9766	172.0
26	176.7114	176.7028	171.8577	174.0963	174.2192	176.6251	176.6726	176.6568	176.5
P	0.23237	0.01821	0.54049	0.01190	0.02721	0.02952	0.05633	0.08397	

Table S7. NMR calculation results of 2d at mPW1PW91/6-31+G(d,p)// B3LYP/6-31G(d) level of theory.

Isotropic values										
GV No.	**2ba**	**2bb**	**2bc**	**2bd**	**2be**	**2bf**	**2bg**	**2bh**	**2bi**	Scaled
1C	76.7888	76.2285	77.0974	76.6676	76.437	77.1596	77.288	76.8312	76.9918	76.9
2C	36.651	36.6589	40.6834	40.4325	36.5046	40.5239	40.4039	36.6036	36.4099	37.9
3C	100.5601	100.7644	100.2802	100.5113	100.8163	100.3871	100.3887	100.6549	100.5927	100.5
4C	41.1697	40.8138	37.1581	36.85	40.7931	36.8453	37.2766	40.9804	41.2819	39.8
5C	87.0343	86.6974	86.5424	86.0817	86.7334	86.2437	85.6259	86.7845	86.0086	86.6
6C	45.7227	45.6154	45.4826	45.4478	45.5963	45.9422	46.4758	46.1489	46.7186	45.9
7C	150.169	150.3265	150.0996	150.4217	150.3904	149.9559	150.3045	149.9977	150.386	150.2
12C	121.1018	121.2968	121.1481	121.0951	121.1197	120.8659	120.8352	120.8438	120.8339	121.0
13C	158.14	158.1513	158.2103	158.0885	158.0761	160.1935	158.1748	160.1519	158.087	158.4
15C	165.5703	165.6243	165.5711	165.5871	165.5948	166.4383	165.5152	166.4431	165.5323	165.7
16C	161.3047	161.346	161.3205	161.3171	161.2925	162.2403	161.2782	162.2325	161.2881	161.4
17C	169.3296	169.3499	169.3216	169.3921	169.4054	171.6386	169.381	171.6542	169.3793	169.7
18C	180.2736	180.1152	180.2791	180.098	180.1036	180.0725	180.1942	180.0666	180.1813	180.2
19C	28.6543	28.8022	29.2927	29.3728	28.675	29.2977	29.3027	28.7197	28.7624	28.9
21C	134.4557	134.7626	134.3005	134.5379	134.6662	134.4951	134.5983	134.6343	134.7207	134.5
22C	162.8232	162.687	162.7091	162.7244	162.7073	162.6425	162.2257	162.733	162.2637	162.7
23C	164.8773	165.0938	165.1369	164.9103	164.7737	165.216	165.3966	164.8881	165.0286	165.0
24C	133.2295	132.8999	133.3109	133.0931	132.9764	133.0764	132.9154	133.0996	132.9055	133.2
26C	29.411	29.6071	29.187	30.299	30.454	29.0837	28.4365	29.3707	28.5165	29.2
27C	140.3386	138.7934	140.3659	137.7337	137.6528	140.2083	140.842	140.1048	140.9438	140.3
29C	32.0649	31.8592	31.9194	32.3409	32.3546	31.9417	30.7791	32.1595	30.9157	31.8
30C	156.6592	160.3024	156.9708	156.8678	156.7844	156.8445	154.5452	156.6168	154.3337	156.4
31C	168.7348	168.6447	168.6477	168.5224	168.4223	168.8496	167.1341	168.8988	167.1563	168.4
33C	171.9805	175.7285	171.9628	171.4404	171.3139	171.9487	176.1043	171.8952	176.0932	172.7
34C	176.7004	171.8514	176.6591	174.1066	174.2177	176.5403	172.4188	176.5647	172.4591	175.7
P	0.41098	0.01523	0.21769	0.01058	0.02867	0.04540	0.06271	0.09009	0.11865	

Table S8. DP4+ analysis details of 2.

PCM-mPW1PW91/6-31G+(d,p)/ Shielding Tensors				
Nuclei	Sp2?	experimental	2a	2b
1C	x	106.7	88.1	86.6
2C	x	160.3	38.9	39.8
3C	x	102.7	100.7	100.5
4C	x	160.3	38.4	37.9
5C	x	115.4	77.3	76.9
6C	x	141.6	44.0	45.9
7C	x	175.5	28.9	28.9
12C		42.4	148.7	150.2
13C		71.9	120.2	121.0
15C		37.0	155.1	158.4
16C		25.1	166.2	165.7
17C		31.5	161.4	161.4
18C		22.2	169.4	169.7
19C		14.0	180.2	180.2
21C		21.7	176.5	175.7
22C		22.9	172.0	172.7
23C	x	170.9	29.4	29.2
24C		60.0	133.2	133.2
26C	x	166.2	32.4	31.8
27C		52.9	140.1	140.3
29C		26.1	164.8	165.0
30C		31.1	162.8	162.7
31C		55.8	135.1	134.5
33C		37.8	157.0	156.4
34C		24.0	168.8	168.4
35H	x	5.82	24.99	25.05
39H		2.55	28.90	28.61
41H		3.63	27.66	27.67
43H		1.32	30.10	30.39
44H		1.40	30.04	30.06
45H		1.28	29.85	30.32
46H		1.41	30.28	29.89
47H		1.25	30.33	30.37
49H		1.27	30.23	30.26
51H		0.86	30.63	30.67
54H		0.81	30.68	30.68
57H		0.80	30.66	30.66
60H		4.29	27.05	27.07
61H		3.88	27.38	27.38
63H		1.98	29.40	29.41
64H		2.63	28.76	28.74
65H		2.24	28.93	28.91
66H		1.79	29.68	29.70
67H		5.14	25.39	25.31
68H		1.63	29.77	29.75
69H		1.24	30.23	30.31
70H		1.81	29.90	29.78

S2.3 Standard orientations.

Standard orientations of **1aa**

C	3.65897	2.16753	0.25562
C	4.54475	1.10285	0.40183
C	4.12161	-0.23063	0.09652
C	2.78005	-0.42347	-0.34940
C	1.90031	0.65981	-0.48538
C	2.35438	1.95531	-0.19150
O	2.26549	-1.63079	-0.66536
O	5.78477	1.38178	0.83408
C	5.09177	-1.30237	0.25499
C	1.43160	3.13727	-0.40301
C	-0.03324	2.71603	-0.54899
O	-0.10216	1.63322	-1.48249
C	-2.12800	-1.26247	1.12061
C	-1.12138	-0.93266	2.07048
C	-1.24789	-1.23732	3.42996
C	-2.40913	-1.88366	3.83734
C	-3.42001	-2.22151	2.91293
C	-3.29016	-1.92006	1.56334
C	-5.34090	2.41972	1.15622
C	-4.62933	2.85146	-0.13019
C	-3.12890	3.10628	0.06686
C	-2.40686	3.48401	-1.23386
C	-0.92156	3.84493	-1.07039
C	0.48291	0.42040	-0.98845
O	4.64071	-2.55357	-0.06104
O	6.25290	-1.17331	0.64091
C	-0.42231	-0.23523	0.03606
C	-1.66455	-0.80066	-0.16102
C	-2.38248	-0.91959	-1.48152
C	-2.04028	-2.23069	-2.17127
O	-1.07595	-2.41220	-2.88378
O	-2.93235	-3.19943	-1.86154
C	-2.64454	-4.49682	-2.40706
N	-0.10745	-0.30067	1.38299
H	4.01545	3.16585	0.48802
H	2.94589	-2.32271	-0.57083
H	6.29020	0.53473	0.87990
H	1.54681	3.85440	0.41966
H	1.74224	3.65868	-1.31989
H	-0.40828	2.37280	0.42710
H	-0.46865	-0.97781	4.14216
H	-2.53954	-2.13441	4.88655
H	-4.31271	-2.73070	3.26610
H	-4.06482	-2.20213	0.85549

H	-5.24504	3.18371	1.93790
H	-6.41068	2.25231	0.98513
H	-4.91495	1.48866	1.54944
H	-4.76424	2.07707	-0.89900
H	-5.10385	3.75986	-0.52853
H	-2.67421	2.20429	0.49753
H	-2.99285	3.90716	0.81017
H	-2.91723	4.34422	-1.68957
H	-2.49290	2.65912	-1.95152
H	-0.81954	4.69755	-0.38354
H	-0.52076	4.17141	-2.03957
H	0.51740	-0.23855	-1.86109
H	5.38702	-3.16494	0.08513
H	-3.46517	-0.85829	-1.33225
H	-2.07803	-0.10912	-2.14839
H	-2.62255	-4.45844	-3.49952
H	-3.45023	-5.14561	-2.06196
H	-1.67824	-4.85870	-2.04556
H	0.77532	-0.00198	1.76934

Standard orientations of **1ab**

C	3.01106	1.91330	0.67167
C	4.06222	1.08978	0.28431
C	3.84830	0.08023	-0.70405
C	2.53419	-0.05047	-1.25329
C	1.48400	0.77567	-0.83636
C	1.73666	1.77181	0.11419
O	2.24283	-0.97966	-2.18306
O	5.25179	1.31218	0.88629
C	4.87916	-0.81766	-1.19419
C	0.64247	2.73967	0.50802
C	-0.73613	2.34110	-0.02799
O	-0.59951	1.89547	-1.39052
C	-1.93182	-2.21533	-0.14835
C	-2.75488	-1.56992	-1.10894
C	-4.05845	-1.99552	-1.38627
C	-4.53706	-3.09669	-0.68438
C	-3.73512	-3.75967	0.26713
C	-2.44215	-3.33008	0.53933
C	-6.12360	0.98385	0.84677
C	-5.36167	1.97887	-0.03452
C	-3.91647	2.21350	0.42572
C	-3.14474	3.17645	-0.48728
C	-1.71868	3.51106	-0.02187
C	0.12094	0.65870	-1.49116
O	6.11433	-0.65462	-0.63095

O	4.73386	-1.68947	-2.05194
C	-0.77048	-0.47713	-1.02476
C	-0.67515	-1.50539	-0.10844
C	0.46353	-1.88676	0.78851
C	0.59288	-1.05859	2.06173
O	0.02076	-0.02130	2.31812
O	1.48330	-1.63752	2.90009
C	1.74224	-0.91877	4.11651
N	-2.01471	-0.53806	-1.63848
H	3.21107	2.68138	1.41197
H	3.07370	-1.47369	-2.39351
H	5.91794	0.69129	0.53968
H	0.60354	2.82132	1.60065
H	0.91281	3.73347	0.12175
H	-1.11856	1.51216	0.58000
H	-4.67311	-1.48793	-2.12515
H	-5.54503	-3.45431	-0.87613
H	-4.13888	-4.61848	0.79645
H	-1.83949	-3.84842	1.28133
H	-7.15821	0.85931	0.50607
H	-5.64514	-0.00251	0.83114
H	-6.15555	1.32264	1.88979
H	-5.89599	2.93972	-0.05570
H	-5.35583	1.61439	-1.07243
H	-3.39883	1.24677	0.47968
H	-3.92476	2.61179	1.45163
H	-3.70779	4.11707	-0.56741
H	-3.09792	2.76445	-1.50401
H	-1.75000	3.91824	0.99887
H	-1.30567	4.30168	-0.66337
H	0.27601	0.53004	-2.56975
H	6.69922	-1.31341	-1.05038
H	0.36776	-2.93543	1.09319
H	1.42799	-1.83007	0.27374
H	2.46149	-1.52550	4.66783
H	2.16144	0.06692	3.89637
H	0.82239	-0.79385	4.69392
H	-2.36811	0.20483	-2.22322

Standard orientations of **1ac**

C	3.00886	1.92152	0.67584
C	4.05978	1.09329	0.29233
C	3.85076	0.07999	-0.69821
C	2.54307	-0.05117	-1.25339
C	1.49441	0.77717	-0.84034
C	1.74061	1.77776	0.11158

O	2.22276	-0.97786	-2.18847
O	5.25242	1.28921	0.87831
C	4.98683	-0.74361	-1.07485
C	0.64287	2.74637	0.49508
C	-0.73381	2.34297	-0.04104
O	-0.59289	1.89086	-1.40116
C	-1.91583	-2.21519	-0.13512
C	-2.73625	-1.58373	-1.10711
C	-4.03599	-2.01867	-1.38782
C	-4.51329	-3.11510	-0.67763
C	-3.71378	-3.76440	0.28526
C	-2.42463	-3.32550	0.56085
C	-6.12119	0.97763	0.81907
C	-5.35800	1.97046	-0.06360
C	-3.91527	2.21072	0.40148
C	-3.14220	3.17147	-0.51270
C	-1.71854	3.51109	-0.04352
C	0.13063	0.65595	-1.49480
O	4.73710	-1.68657	-2.03505
O	6.12421	-0.66285	-0.61222
C	-0.75762	-0.48021	-1.02243
C	-0.66283	-1.49857	-0.09491
C	0.47051	-1.86443	0.81528
C	0.57762	-1.03052	2.08728
O	0.00100	0.00806	2.32781
O	1.45422	-1.60493	2.94207
C	1.69845	-0.87812	4.15717
N	-1.99770	-0.55403	-1.64343
H	3.20695	2.69066	1.41548
H	3.01515	-1.49117	-2.43285
H	5.88925	0.63673	0.49980
H	0.60151	2.83666	1.58683
H	0.91268	3.73736	0.10141
H	-1.11648	1.51658	0.57012
H	-4.64893	-1.52143	-2.13511
H	-5.51844	-3.47948	-0.87145
H	-4.11657	-4.61959	0.82112
H	-1.82409	-3.83279	1.31215
H	-7.15402	0.84883	0.47456
H	-5.64016	-0.00758	0.81024
H	-6.15846	1.32122	1.86032
H	-5.89455	2.92989	-0.09129
H	-5.34706	1.60138	-1.09986
H	-3.39546	1.24552	0.46202
H	-3.92870	2.61354	1.42553
H	-3.70697	4.11050	-0.59888

H	-3.09076	2.75521	-1.52750
H	-1.75432	3.92248	0.97535
H	-1.30487	4.29985	-0.68686
H	0.28443	0.52239	-2.57283
H	5.57751	-2.15874	-2.18586
H	0.38414	-2.91331	1.12190
H	1.44035	-1.79530	0.31237
H	2.41054	-1.48154	4.72109
H	2.12115	0.10561	3.93539
H	0.77134	-0.74913	4.72189
H	-2.35289	0.18359	-2.23383

Standard orientations of **1ad**

C	2.64622	2.63267	1.08681
C	3.76704	1.86304	0.77816
C	3.69134	0.85539	-0.23419
C	2.44025	0.64781	-0.88035
C	1.31392	1.39948	-0.52336
C	1.43058	2.41104	0.43946
O	2.24272	-0.26014	-1.85725
O	4.89825	2.11411	1.45726
C	4.89274	0.08472	-0.52063
C	0.23576	3.28744	0.75114
C	-1.08029	2.69206	0.24235
O	-0.89519	2.24859	-1.10982
C	-1.73049	-2.00518	0.04858
C	-2.57087	-1.47145	-0.96349
C	-3.80005	-2.04802	-1.30146
C	-4.18720	-3.18661	-0.60340
C	-3.36744	-3.73779	0.40251
C	-2.14632	-3.16226	0.73216
C	-6.21904	0.56331	1.07721
C	-5.60681	1.66820	0.21037
C	-4.20739	2.09684	0.67242
C	-3.58277	3.17332	-0.22624
C	-2.22168	3.70844	0.24643
C	-0.00644	1.12407	-1.21263
O	4.78461	-0.81727	-1.53463
O	5.96998	0.19512	0.06644
C	-0.74822	-0.13265	-0.78379
C	-0.57757	-1.13708	0.15299
C	0.54011	-1.33958	1.15049
C	1.48395	-2.47587	0.77275
O	2.50598	-2.37044	0.12558
O	1.04456	-3.65963	1.25438
C	1.83829	-4.80589	0.90599

N	-1.93663	-0.35879	-1.46549
H	2.74639	3.40306	1.84462
H	2.99545	-0.87963	-1.88738
H	5.58704	1.48023	1.13950
H	0.17576	3.47287	1.83096
H	0.38976	4.26581	0.27309
H	-1.34646	1.82449	0.86513
H	-4.42779	-1.62315	-2.08045
H	-5.13585	-3.66065	-0.84030
H	-3.69852	-4.63026	0.92676
H	-1.51767	-3.60264	1.49924
H	-7.22753	0.30175	0.73623
H	-5.60895	-0.34702	1.04415
H	-6.29418	0.87801	2.12560
H	-6.27020	2.54502	0.20450
H	-5.55229	1.32333	-0.83255
H	-3.55939	1.21076	0.70715
H	-4.26814	2.47303	1.70512
H	-4.27652	4.02279	-0.29630
H	-3.47496	2.78415	-1.24691
H	-2.31476	4.10658	1.26698
H	-1.92358	4.55021	-0.39311
H	0.17752	1.03547	-2.28996
H	5.63392	-1.29584	-1.56908
H	1.14377	-0.43794	1.24985
H	0.10813	-1.57191	2.13084
H	1.34104	-5.65788	1.37010
H	1.87691	-4.92869	-0.17953
H	2.85617	-4.69807	1.28988
H	-2.35165	0.33226	-2.07304

Standard orientations of **1ae**

C	2.64696	-2.63227	-1.08649
C	3.76754	-1.86234	-0.77780
C	3.69153	-0.85464	0.23446
C	2.44035	-0.64730	0.88049
C	1.31422	-1.39929	0.52349
C	1.43120	-2.41088	-0.43925
O	2.24252	0.26070	1.85730
O	4.89887	-2.11319	-1.45680
C	4.89269	-0.08347	0.52076
C	0.23660	-3.28754	-0.75107
C	-1.07960	-2.69233	-0.24250
O	-0.89474	-2.24895	1.10973
C	-1.73109	2.00453	-0.04839
C	-2.57101	1.47099	0.96417

C	-3.80010	2.04754	1.30247
C	-4.18765	3.18590	0.60425
C	-3.36834	3.73690	-0.40212
C	-2.14730	3.16141	-0.73210
C	-6.21807	-0.56333	-1.07760
C	-5.60595	-1.66827	-0.21073
C	-4.20657	-2.09704	-0.67280
C	-3.58208	-3.17365	0.22578
C	-2.22095	-3.70875	-0.24683
C	-0.00622	-1.12427	1.21275
O	4.78424	0.81853	1.53473
O	5.96993	-0.19350	-0.06633
C	-0.74823	0.13239	0.78414
C	-0.57811	1.13655	-0.15302
C	0.53924	1.33905	-1.15092
C	1.48300	2.47551	-0.77350
O	2.50520	2.37024	-0.12659
O	1.04328	3.65920	-1.25509
C	1.83681	4.80561	-0.90679
N	-1.93643	0.35854	1.46621
H	2.74737	-3.40268	-1.84426
H	2.99529	0.88012	1.88766
H	5.58737	-1.47899	-1.13914
H	0.17680	-3.47302	-1.83089
H	0.39070	-4.26587	-0.27296
H	-1.34570	-1.82474	-0.86527
H	-4.42750	1.62283	2.08182
H	-5.13626	3.65991	0.84139
H	-3.69974	4.62921	-0.92646
H	-1.51899	3.60165	-1.49955
H	-7.22655	-0.30170	-0.73665
H	-5.60792	0.34696	-1.04451
H	-6.29319	-0.87803	-2.12599
H	-6.26943	-2.54502	-0.20485
H	-5.55138	-1.32336	0.83217
H	-3.55848	-1.21103	-0.70744
H	-4.26736	-2.47314	-1.70552
H	-4.27587	-4.02311	0.29566
H	-3.47435	-2.78463	1.24651
H	-2.31394	-4.10677	-1.26743
H	-1.92290	-4.55059	0.39265
H	0.17768	-1.03584	2.29011
H	5.63332	1.29750	1.56907
H	1.14304	0.43750	-1.25029
H	0.10695	1.57118	-2.13118
H	1.87681	4.92757	0.17878

H	2.85425	4.69870	-1.29212
H	1.33850	5.65770	-1.36958
H	-2.35108	-0.33230	2.07423

Standard orientations of **1af**

C	3.54780	2.26740	0.27717
C	4.47722	1.24354	0.43120
C	4.10918	-0.10320	0.12916
C	2.77427	-0.35029	-0.32209
C	1.84828	0.69368	-0.46292
C	2.25104	2.00428	-0.17264
O	2.34543	-1.58447	-0.63316
O	5.70099	1.60847	0.87093
C	5.00607	-1.24189	0.24196
C	1.28561	3.15038	-0.39010
C	-0.16075	2.67363	-0.55420
O	-0.17719	1.58372	-1.48254
C	-2.11763	-1.34349	1.15530
C	-1.10847	-1.00069	2.09625
C	-1.21619	-1.31701	3.45485
C	-2.36137	-1.98849	3.86704
C	-3.37318	-2.34189	2.94924
C	-3.26246	-2.02949	1.60048
C	-5.47131	2.20835	1.10526
C	-4.76263	2.65009	-0.17934
C	-3.27453	2.96170	0.02952
C	-2.55293	3.35050	-1.26822
C	-1.08326	3.76604	-1.09334
C	0.44339	0.39734	-0.96838
O	6.26705	-0.95111	0.68166
O	4.72666	-2.41190	-0.01856
C	-0.44309	-0.27882	0.05822
C	-1.67678	-0.86524	-0.12891
C	-2.42797	-0.98111	-1.43002
C	-2.37740	-2.40039	-1.97311
O	-3.21104	-3.25794	-1.77551
O	-1.24882	-2.60610	-2.69049
C	-1.06512	-3.94490	-3.17704
N	-0.11350	-0.34503	1.40268
H	3.86230	3.28033	0.50711
H	3.09546	-2.21469	-0.49766
H	6.27641	0.82473	0.93703
H	1.36317	3.86796	0.43664
H	1.58742	3.68769	-1.30083
H	-0.53561	2.32063	0.41820
H	-0.43540	-1.04848	4.16202

H	-2.47703	-2.24974	4.91549
H	-4.25015	-2.87493	3.30638
H	-4.02936	-2.33068	0.89260
H	-5.01608	1.29824	1.51428
H	-5.41153	2.98491	1.87813
H	-6.53226	1.99970	0.92534
H	-5.26556	3.53572	-0.59385
H	-4.86165	1.86200	-0.93969
H	-2.79187	2.08200	0.47562
H	-3.17541	3.77566	0.76445
H	-3.08866	4.18692	-1.73881
H	-2.60175	2.51515	-1.97716
H	-1.01883	4.62690	-0.41220
H	-0.68420	4.09947	-2.06096
H	0.51146	-0.26845	-1.83414
H	6.75502	-1.79580	0.70693
H	-3.48437	-0.73264	-1.28367
H	-2.01145	-0.29002	-2.16711
H	-1.88661	-4.22959	-3.84012
H	-0.11989	-3.93207	-3.72064
H	-1.02008	-4.65220	-2.34454
H	0.76972	-0.03895	1.78158

Standard orientations of **1ag**

C	-2.85464	-2.61684	0.54843
C	-4.13245	-2.07628	0.44288
C	-4.32240	-0.81972	-0.20927
C	-3.16825	-0.15250	-0.72916
C	-1.88754	-0.70648	-0.60060
C	-1.74240	-1.95176	0.02605
O	-3.25574	1.03152	-1.36191
O	-5.13533	-2.80294	0.98188
C	-5.61382	-0.17789	-0.39063
C	-0.37911	-2.60825	0.09262
C	0.76438	-1.65592	-0.27508
O	0.38038	-0.91663	-1.44377
C	0.86278	3.02663	0.42666
C	-0.22196	2.71570	1.29137
C	-0.51772	3.47886	2.42580
C	0.29458	4.57512	2.69115
C	1.37633	4.90315	1.84745
C	1.66832	4.14175	0.72302
C	6.05559	-5.11911	1.03652
C	4.68379	-4.52966	1.38040
C	4.05894	-3.73839	0.22440
C	2.68577	-3.14232	0.56175

C	2.07915	-2.35587	-0.60838
C	-0.68127	0.00499	-1.19837
O	-6.68132	-0.85509	0.12878
O	-5.81518	0.89633	-0.95717
C	-0.20032	1.19384	-0.38135
C	0.85761	2.04474	-0.62743
C	1.82613	1.96911	-1.77802
C	3.25757	1.74088	-1.30808
O	3.67917	0.73289	-0.78292
O	4.03344	2.82713	-1.54152
C	5.39973	2.70881	-1.11113
N	-0.83629	1.59373	0.78177
H	-2.74767	-3.57907	1.03892
H	-4.20900	1.29325	-1.39463
H	-5.98519	-2.34569	0.84675
H	-0.22300	-3.03296	1.09165
H	-0.37478	-3.45489	-0.60972
H	0.93599	-0.94707	0.54963
H	-1.35198	3.22579	3.07524
H	0.09184	5.19020	3.56375
H	1.99036	5.76794	2.08456
H	2.50510	4.40044	0.08037
H	6.76960	-4.33019	0.76980
H	6.47591	-5.67709	1.88122
H	5.98946	-5.80586	0.18348
H	4.00164	-5.33959	1.67689
H	4.77686	-3.87449	2.25826
H	3.96494	-4.39406	-0.65434
H	4.73923	-2.92733	-0.07226
H	2.78700	-2.47789	1.43254
H	2.01068	-3.95396	0.87003
H	1.91225	-3.02341	-1.46595
H	2.78004	-1.57858	-0.93019
H	-0.95768	0.36932	-2.19407
H	-7.47291	-0.31887	-0.06636
H	1.56984	1.11968	-2.41714
H	1.79701	2.88391	-2.38001
H	5.44823	2.54366	-0.03158
H	5.87328	3.65438	-1.37717
H	5.89130	1.87549	-1.62011
H	-1.67038	1.15952	1.14537

Standard orientations of **1ah**

C	-2.90597	-2.06407	0.62033
C	-4.00283	-1.29711	0.23817
C	-3.84720	-0.25493	-0.73208

C	-2.54411	-0.03109	-1.26829
C	-1.44956	-0.79832	-0.85586
C	-1.64301	-1.83021	0.07475
O	-2.27283	0.92956	-2.18423
O	-5.18727	-1.57858	0.80569
C	-5.02900	0.50133	-1.10928
C	-0.49017	-2.73443	0.45419
C	0.86376	-2.22457	-0.04648
O	0.71536	-1.76170	-1.40237
C	1.74403	2.41040	-0.07906
C	2.60618	1.85933	-1.06379
C	3.86728	2.39710	-1.34171
C	4.26228	3.51504	-0.61559
C	3.42061	4.08484	0.36148
C	2.17015	3.54408	0.63455
C	6.47712	-2.55328	0.10738
C	5.41990	-1.44571	0.19250
C	4.01459	-1.92752	0.59175
C	3.34504	-2.85081	-0.43729
C	1.93162	-3.31689	-0.05039
C	-0.08892	-0.57774	-1.49080
O	-4.82879	1.47482	-2.05040
O	-6.16421	0.34051	-0.66243
C	0.71483	0.61064	-0.99641
C	0.54767	1.60206	-0.05003
C	-0.60712	1.86791	0.86807
C	-0.63599	1.02276	2.13673
O	-0.00385	0.01110	2.35172
O	-1.51517	1.54673	3.02076
C	-1.68885	0.79979	4.23577
N	1.94508	0.78653	-1.61625
H	-3.06326	-2.85725	1.34418
H	-3.09315	1.39656	-2.42919
H	-5.85987	-0.96110	0.43041
H	-0.46089	-2.85096	1.54388
H	-0.68570	-3.73041	0.03021
H	1.17207	-1.38265	0.58477
H	4.51341	1.95968	-2.09845
H	5.23596	3.95815	-0.80625
H	3.75947	4.95938	0.91027
H	1.53613	3.99150	1.39636
H	6.24099	-3.28902	-0.66973
H	7.46470	-2.13780	-0.12449
H	6.55947	-3.09253	1.05969
H	5.75001	-0.69379	0.92122
H	5.36051	-0.92140	-0.77251

H	3.38389	-1.04580	0.76069
H	4.07414	-2.44949	1.55908
H	3.96593	-3.74325	-0.58741
H	3.29730	-2.34538	-1.41132
H	1.95529	-3.77183	0.95017
H	1.60420	-4.10233	-0.74528
H	-0.23885	-0.44156	-2.56912
H	-5.69577	1.89526	-2.20385
H	-0.60976	2.91944	1.17700
H	-1.57037	1.71484	0.37085
H	-2.41438	1.36250	4.82410
H	-0.74105	0.71386	4.77370
H	-2.06726	-0.20264	4.01775
H	2.35125	0.09101	-2.22448

Standard orientations of **1ai**

C	3.41943	-2.36704	-0.94170
C	4.56112	-1.70814	-0.49176
C	4.44485	-0.60965	0.41974
C	3.13697	-0.21803	0.83408
C	1.99941	-0.88677	0.36314
C	2.15127	-1.97116	-0.51539
O	2.90076	0.80015	1.69168
O	5.74757	-2.14380	-0.94386
C	5.67063	0.03342	0.86556
C	0.93134	-2.74847	-0.96405
C	-0.38531	-2.02164	-0.66745
O	-0.33536	-1.50866	0.66851
C	-0.93105	2.73333	-0.43173
C	0.15119	2.52416	-1.32885
C	0.44113	3.40850	-2.37358
C	-0.37659	4.52314	-2.51526
C	-1.46003	4.74914	-1.64049
C	-1.74541	3.86615	-0.60770
C	-6.70443	-3.32780	-0.71515
C	-5.48094	-2.48646	-0.33757
C	-4.15133	-3.12709	-0.75675
C	-2.93135	-2.25129	-0.44575
C	-1.60157	-2.93767	-0.78120
C	0.61638	-0.45074	0.82837
O	5.50404	1.06986	1.74210
O	6.81200	-0.27308	0.52427
C	0.14483	0.83299	0.17252
C	-0.91598	1.64572	0.51200
C	-1.84415	1.45640	1.67090
C	-3.30729	1.25151	1.29304

O	-3.75867	1.11770	0.17801
O	-4.07146	1.24030	2.41248
C	-5.47754	1.04549	2.19629
N	0.77259	1.35526	-0.94792
H	3.54553	-3.20198	-1.62340
H	3.74361	1.20196	1.97318
H	6.45388	-1.58729	-0.53613
H	1.00833	-2.98183	-2.03360
H	0.92908	-3.71203	-0.43434
H	-0.50781	-1.17919	-1.36620
H	1.27356	3.23075	-3.04985
H	-0.17989	5.23090	-3.31604
H	-2.08386	5.62719	-1.78443
H	-2.59231	4.04489	0.04904
H	-6.66366	-4.32049	-0.24939
H	-7.63662	-2.84811	-0.39467
H	-6.76336	-3.47543	-1.80065
H	-5.55999	-1.48933	-0.79157
H	-5.47099	-2.32447	0.75040
H	-4.17897	-3.34345	-1.83567
H	-4.04604	-4.10186	-0.25602
H	-2.93010	-1.98280	0.61737
H	-3.01117	-1.30294	-0.99295
H	-1.62867	-3.32694	-1.80953
H	-1.45432	-3.80543	-0.12251
H	0.64836	-0.28394	1.90965
H	6.39631	1.40407	1.95267
H	-1.55076	0.58665	2.27015
H	-1.81025	2.31443	2.35679
H	-5.88259	1.83596	1.55847
H	-5.93387	1.08078	3.18633
H	-5.66448	0.07833	1.72190
H	1.61816	0.97696	-1.34659

Standard orientations of **1aj**

C	-3.23820	-1.83391	0.86078
C	-4.24945	-0.93458	0.53438
C	-4.02750	0.06393	-0.46880
C	-2.74805	0.10679	-1.09807
C	-1.73740	-0.79367	-0.74078
C	-1.99663	-1.77647	0.22631
O	-2.42173	0.99888	-2.06173
O	-5.41757	-1.04838	1.18738
C	-5.12300	0.96612	-0.78098
C	-0.93773	-2.80629	0.55751
C	0.44144	-2.44244	0.00210

O	0.29290	-1.99175	-1.35636
C	1.77996	2.10789	-0.27145
C	2.36823	1.65645	-1.48218
C	3.53225	2.22948	-2.00653
C	4.10775	3.27675	-1.29502
C	3.53868	3.74321	-0.09231
C	2.38345	3.16979	0.42511
C	5.33832	-1.48246	-1.17208
C	5.10442	-2.25393	0.13256
C	3.62470	-2.43744	0.51048
C	2.82866	-3.30947	-0.47262
C	1.39666	-3.63417	-0.01769
C	-0.40090	-0.74334	-1.45799
O	-4.86316	1.89149	-1.75533
O	-6.23465	0.96451	-0.25342
C	0.52089	0.40069	-1.08126
C	0.60876	1.29779	-0.03397
C	-0.28889	1.46881	1.16359
C	0.34984	0.92846	2.43653
O	0.58124	-0.24129	2.66261
O	0.63988	1.91970	3.30864
C	1.26554	1.49943	4.53348
N	1.57508	0.63668	-1.95490
H	-3.44482	-2.58426	1.61702
H	-3.18308	1.57834	-2.24988
H	-6.02784	-0.35188	0.84571
H	-0.87700	-2.93409	1.64482
H	-1.25635	-3.77225	0.13865
H	0.85737	-1.62915	0.60875
H	3.96868	1.87308	-2.93594
H	5.01252	3.74397	-1.67429
H	4.01590	4.56297	0.43788
H	1.95989	3.52576	1.36033
H	4.87642	-0.48827	-1.13022
H	6.40957	-1.34388	-1.35982
H	4.92044	-2.01048	-2.03717
H	5.58423	-3.24129	0.06350
H	5.61176	-1.72633	0.95150
H	3.15416	-1.44944	0.60489
H	3.57185	-2.89463	1.50937
H	3.36677	-4.25729	-0.61818
H	2.77972	-2.82764	-1.45596
H	1.41829	-4.06906	0.99170
H	0.96708	-4.39701	-0.68158
H	-0.60302	-0.64153	-2.53173
H	-5.67507	2.42296	-1.85685

H	-0.53037	2.52644	1.31103
H	-1.22406	0.92273	1.02848
H	1.42631	2.41224	5.10769
H	2.21633	1.00192	4.32555
H	0.61516	0.81066	5.07892
H	1.79756	0.03098	-2.73074

Standard orientations of **1ak**

C	2.59362	2.70137	0.94638
C	3.72823	1.93333	0.69665
C	3.65894	0.83688	-0.21233
C	2.40078	0.54794	-0.81839
C	1.26037	1.30651	-0.52682
C	1.37255	2.40144	0.33799
O	2.24493	-0.47033	-1.68408
O	4.85401	2.30198	1.34622
C	4.77864	-0.03248	-0.53837
C	0.17224	3.29236	0.57944
C	-1.13807	2.66034	0.10018
O	-0.93504	2.11168	-1.21004
C	-1.75235	-2.06675	0.25968
C	-2.61792	-1.59433	-0.76110
C	-3.84613	-2.20256	-1.04335
C	-4.20109	-3.31252	-0.28428
C	-3.35094	-3.80769	0.72596
C	-2.13250	-3.19910	1.00011
C	-6.29030	0.61353	1.04120
C	-5.66878	1.65044	0.10015
C	-4.27121	2.10795	0.53975
C	-3.63401	3.10907	-0.43397
C	-2.27619	3.67643	0.00958
C	-0.05322	0.97642	-1.20592
O	5.93979	0.23894	0.12685
O	4.76391	-0.95778	-1.34918
C	-0.80391	-0.23567	-0.68396
C	-0.60910	-1.18508	0.30172
C	0.52538	-1.32562	1.28095
C	1.64885	-2.28170	0.89277
O	2.82674	-2.07741	1.10546
O	1.18488	-3.41427	0.33091
C	2.18854	-4.36613	-0.05558
N	-2.00316	-0.50414	-1.33281
H	2.68622	3.54159	1.62709
H	3.12512	-0.91240	-1.78872
H	5.57436	1.68134	1.13204
H	0.10203	3.54867	1.64420

H	0.32906	4.23770	0.03986
H	-1.41469	1.84445	0.78507
H	-4.49602	-1.82593	-1.82901
H	-5.14735	-3.81034	-0.47814
H	-3.65491	-4.68275	1.29404
H	-1.47780	-3.60069	1.76861
H	-7.29726	0.33030	0.71331
H	-5.68264	-0.29828	1.07907
H	-6.37143	1.00424	2.06323
H	-6.32929	2.52625	0.02520
H	-5.60829	1.23007	-0.91441
H	-3.62764	1.22472	0.64832
H	-4.33888	2.56306	1.53973
H	-4.32416	3.95216	-0.57790
H	-3.51707	2.64101	-1.41997
H	-2.37934	4.15532	0.99386
H	-1.96797	4.46351	-0.69186
H	0.14833	0.79800	-2.26950
H	6.59073	-0.42428	-0.17001
H	1.00105	-0.37063	1.50421
H	0.12197	-1.70224	2.23249
H	1.64674	-5.18472	-0.52998
H	2.89514	-3.91260	-0.75553
H	2.73769	-4.72390	0.82000
H	-2.42987	0.14658	-1.97546

Standard orientations of **1al**

C	3.62827	-1.96670	-1.03420
C	4.64930	-1.09310	-0.67380
C	4.38000	-0.01506	0.22392
C	3.04603	0.12994	0.71893
C	2.02639	-0.75337	0.33485
C	2.33311	-1.81120	-0.53195
O	2.70690	1.10793	1.57489
O	5.86224	-1.33452	-1.21693
C	5.37618	0.94260	0.67580
C	1.25929	-2.81454	-0.89692
C	-0.14593	-2.29590	-0.58091
O	-0.14641	-1.77646	0.75318
C	-1.55424	2.31469	-0.09581
C	-0.63051	2.22202	-1.17216
C	-0.66691	3.09096	-2.26829
C	-1.65599	4.06698	-2.27858
C	-2.59144	4.16960	-1.22736
C	-2.55384	3.30321	-0.14309
C	-5.18448	-0.78140	-2.62730

C	-4.64295	-1.57793	-1.43569
C	-3.18955	-2.03187	-1.62783
C	-2.65478	-2.87529	-0.46110
C	-1.21582	-3.38441	-0.64819
C	0.62357	-0.57632	0.90010
O	6.63216	0.76306	0.16767
O	5.18239	1.87485	1.45567
C	-0.11100	0.62002	0.33776
C	-1.19981	1.29136	0.85421
C	-1.84562	1.03333	2.18726
C	-3.23714	0.41961	2.10110
O	-4.15125	0.84311	1.42577
O	-3.34950	-0.65279	2.91669
C	-4.64958	-1.26507	2.96016
N	0.21583	1.17021	-0.89263
H	3.86849	-2.78046	-1.71088
H	3.51457	1.64731	1.76232
H	6.50498	-0.67520	-0.89774
H	1.34282	-3.08469	-1.95728
H	1.43400	-3.73590	-0.32256
H	-0.39646	-1.48711	-1.28342
H	0.04968	3.00561	-3.08137
H	-1.71364	4.75881	-3.11467
H	-3.36037	4.93598	-1.27512
H	-3.29836	3.36425	0.64416
H	-5.15564	-1.37453	-3.55041
H	-6.22288	-0.47253	-2.46130
H	-4.59213	0.12605	-2.79475
H	-4.71070	-0.96025	-0.53077
H	-5.27833	-2.45988	-1.26502
H	-2.56226	-1.14088	-1.76788
H	-3.11061	-2.61396	-2.55946
H	-3.31247	-3.74517	-0.32378
H	-2.70250	-2.29839	0.47078
H	-1.12546	-3.89827	-1.61610
H	-0.98277	-4.12635	0.12735
H	0.69984	-0.43980	1.98341
H	7.19086	1.46495	0.55167
H	-1.22032	0.39119	2.81146
H	-1.96962	1.98450	2.72570
H	-5.41343	-0.52732	3.21897
H	-4.58218	-2.03734	3.72715
H	-4.89580	-1.70948	1.99243
H	1.04228	0.92310	-1.41558

Standard orientations of **1am**

C	2.38712	-2.86594	-0.88288
C	3.57668	-2.18893	-0.62570
C	3.58220	-1.07335	0.26241
C	2.34195	-0.67172	0.84061
C	1.14789	-1.33905	0.54107
C	1.18448	-2.45516	-0.30321
O	2.25606	0.37147	1.68633
O	4.67912	-2.66056	-1.24798
C	4.76253	-0.28920	0.58977
C	-0.08218	-3.24544	-0.55576
C	-1.34123	-2.48907	-0.12286
O	-1.12306	-1.94314	1.18597
C	-1.53713	2.28622	-0.31258
C	-2.44502	1.91442	0.71308
C	-3.60311	2.64859	0.99138
C	-3.84299	3.78240	0.22340
C	-2.94933	4.17776	-0.79321
C	-1.80091	3.44421	-1.06380
C	-6.93785	-1.94324	-0.28425
C	-5.72554	-1.00991	-0.38739
C	-4.40528	-1.70925	-0.75450
C	-3.88761	-2.68482	0.31312
C	-2.57153	-3.39253	-0.04947
C	-0.14487	-0.89014	1.19224
O	5.90856	-0.66705	-0.04866
O	4.80913	0.65264	1.38062
C	-0.77552	0.38076	0.65271
C	-0.48770	1.29445	-0.34391
C	0.65559	1.30932	-1.32233
C	1.87156	2.15025	-0.94599
O	3.02009	1.83413	-1.18200
O	1.53010	3.31668	-0.36667
C	2.62918	4.16059	0.01120
N	-1.94298	0.77361	1.29593
H	2.42165	-3.72298	-1.54785
H	3.16803	0.74036	1.80137
H	5.44389	-2.09581	-1.03322
H	-0.14950	-3.51903	-1.61640
H	-0.02220	-4.18792	0.00778
H	-1.52084	-1.66100	-0.82517
H	-4.28725	2.34729	1.78052
H	-4.73267	4.37669	0.41359
H	-3.16288	5.07432	-1.36896
H	-1.11026	3.76899	-1.83723
H	-7.85318	-1.37676	-0.07760
H	-7.09252	-2.49278	-1.22155

H	-6.82254	-2.68177	0.51711
H	-5.59542	-0.47116	0.56237
H	-5.93251	-0.23971	-1.14185
H	-3.64981	-0.93572	-0.94260
H	-4.53592	-2.24924	-1.70501
H	-4.64361	-3.45794	0.49988
H	-3.75311	-2.15245	1.26393
H	-2.67953	-3.89887	-1.01938
H	-2.36502	-4.17580	0.69232
H	0.05230	-0.72302	2.25862
H	6.60555	-0.05066	0.24461
H	1.03259	0.30867	-1.53445
H	0.29323	1.71318	-2.27928
H	3.19177	4.47855	-0.87124
H	2.17930	5.01958	0.50963
H	3.30312	3.62873	0.68786
H	-2.42994	0.18022	1.95098

Standard orientations of **1an**

C	-3.33270	-2.53127	0.77903
C	-4.47063	-1.81163	0.42373
C	-4.35194	-0.62964	-0.37619
C	-3.04639	-0.21984	-0.77817
C	-1.91049	-0.94988	-0.40029
C	-2.06625	-2.11512	0.36554
O	-2.80974	0.87142	-1.53866
O	-5.65560	-2.27024	0.85683
C	-5.57445	0.07599	-0.72747
C	-0.85221	-2.95174	0.70693
C	0.46614	-2.19661	0.50819
O	0.43332	-1.53546	-0.75980
C	0.83933	2.75041	0.50130
C	0.00254	2.28694	1.55289
C	-0.18079	3.00775	2.73770
C	0.49076	4.21834	2.86145
C	1.32636	4.69809	1.83141
C	1.50863	3.97931	0.65684
C	6.74402	-3.72775	0.35064
C	5.58054	-2.73061	0.34978
C	4.20471	-3.40496	0.41607
C	3.02980	-2.41858	0.45482
C	1.67332	-3.13139	0.50831
C	-0.53126	-0.47961	-0.84634
O	-5.40639	1.19205	-1.49917
O	-6.71387	-0.24407	-0.39232
C	-0.08065	0.77468	-0.12901

C	0.77430	1.76907	-0.55343
C	1.47150	1.84443	-1.88681
C	2.96831	2.09442	-1.76433
O	3.51910	3.14490	-2.02061
O	3.62636	1.00360	-1.31865
C	5.03895	1.18336	-1.12335
N	-0.52509	1.07898	1.15077
H	-3.45965	-3.42868	1.37591
H	-3.64970	1.31816	-1.75344
H	-6.35930	-1.66340	0.52297
H	-0.92993	-3.32414	1.73621
H	-0.85057	-3.83652	0.05392
H	0.58569	-1.44585	1.30591
H	-0.82511	2.63681	3.53087
H	0.36928	4.80497	3.76812
H	1.83655	5.64864	1.96197
H	2.15714	4.35515	-0.12869
H	6.68119	-4.41074	-0.50550
H	7.71175	-3.21583	0.29768
H	6.74031	-4.33920	1.26142
H	5.68839	-2.04119	1.19947
H	5.63267	-2.11130	-0.55704
H	4.16541	-4.05383	1.30446
H	4.09061	-4.07330	-0.45058
H	3.05657	-1.75871	-0.42055
H	3.13152	-1.76496	1.33374
H	1.61945	-3.75913	1.40961
H	1.57489	-3.80844	-0.35190
H	-0.57139	-0.24683	-1.91437
H	-6.29586	1.56252	-1.65345
H	1.32465	0.91006	-2.43754
H	1.07604	2.66814	-2.49163
H	5.52968	1.42869	-2.06922
H	5.40376	0.23244	-0.73584
H	5.22620	1.98842	-0.40802
H	-1.25862	0.57278	1.62347

Standard orientations of **1ba**

C	-3.14353	-2.69762	0.78348
C	-4.21296	-1.97478	0.26291
C	-3.96738	-0.78332	-0.49011
C	-2.61522	-0.35790	-0.65616
C	-1.55371	-1.05308	-0.05860
C	-1.83253	-2.25123	0.62070
O	-2.27176	0.71397	-1.40348
O	-5.44904	-2.45256	0.47627

C	-5.11830	-0.08924	-1.04668
C	-0.68692	-3.09922	1.12873
C	0.59223	-2.77480	0.35824
O	0.82083	-1.36906	0.47654
C	0.56745	3.00597	0.82359
C	0.33125	2.32232	2.04664
C	0.41462	2.96356	3.28671
C	0.73598	4.31664	3.28839
C	0.97112	5.01437	2.08572
C	0.89132	4.37356	0.85531
C	6.12035	-2.27093	-0.27649
C	5.61780	-3.71744	-0.19630
C	4.34329	-3.90262	0.64411
C	3.09686	-3.21749	0.06764
C	1.83261	-3.48519	0.89399
C	-0.11811	-0.53493	-0.21540
O	-4.83509	1.03510	-1.77181
O	-6.29479	-0.42488	-0.91754
C	0.10352	0.84053	0.35672
C	0.41178	2.04209	-0.23821
C	0.54283	2.33478	-1.70938
C	1.99282	2.56318	-2.10670
O	2.53037	3.64498	-2.21014
O	2.63385	1.38916	-2.30840
C	4.03345	1.49890	-2.61082
N	0.02394	1.01582	1.72929
H	-3.35702	-3.62334	1.30792
H	-3.07176	1.15494	-1.74542
H	-6.09105	-1.84657	0.03402
H	-0.94601	-4.16103	1.03708
H	-0.50824	-2.90663	2.19654
H	0.45204	-3.03362	-0.70540
H	0.23402	2.42634	4.21432
H	0.80938	4.84494	4.23527
H	1.22615	6.06994	2.12570
H	1.10203	4.90819	-0.06659
H	6.31371	-1.86451	0.72419
H	7.05490	-2.21018	-0.84596
H	5.39328	-1.61202	-0.76452
H	6.41170	-4.34658	0.22826
H	5.43866	-4.09952	-1.21173
H	4.52498	-3.52740	1.66248
H	4.14290	-4.97896	0.74835
H	2.93562	-3.56849	-0.96309
H	3.24774	-2.13429	0.00674
H	1.99138	-3.16302	1.93273

H	1.62568	-4.56428	0.92636
H	0.14056	-0.50802	-1.28418
H	-5.69011	1.39726	-2.07172
H	0.12757	1.51525	-2.30030
H	-0.00407	3.24850	-1.96492
H	4.39137	0.47440	-2.72034
H	4.18525	2.05947	-3.53744
H	4.56258	2.00603	-1.79964
H	0.01051	0.24219	2.37647

Standard orientations of **1bb**

C	-1.54285	-3.15786	0.31728
C	-2.77271	-2.78891	-0.22084
C	-2.87000	-1.62907	-1.05151
C	-1.69164	-0.85944	-1.27269
C	-0.47771	-1.19974	-0.65944
C	-0.40704	-2.37716	0.10049
O	-1.65316	0.21164	-2.09314
O	-3.83207	-3.56768	0.05591
C	-4.17707	-1.29182	-1.59150
C	0.92433	-2.81558	0.67083
C	2.08176	-2.14350	-0.06757
O	1.85189	-0.73161	-0.05929
C	0.08667	3.16741	0.36745
C	0.49637	3.35991	-0.98049
C	0.57981	4.62824	-1.56384
C	0.24859	5.72218	-0.77235
C	-0.15312	5.55677	0.56942
C	-0.23604	4.29453	1.14334
C	7.22419	0.04217	-0.42953
C	7.18123	-1.48536	-0.30233
C	5.97078	-2.02494	0.47780
C	4.61602	-1.78490	-0.20239
C	3.44275	-2.39059	0.57799
C	0.74927	-0.31145	-0.86744
O	-4.21960	-0.17637	-2.37916
O	-5.21959	-1.91783	-1.39988
C	0.51751	1.14406	-0.54450
C	0.10960	1.75083	0.62193
C	-0.22767	1.07783	1.91523
C	-1.71061	0.77821	2.10988
O	-2.59116	0.90558	1.28849
O	-1.92678	0.31319	3.36418
C	-3.28301	-0.05694	3.65670
N	0.77354	2.11366	-1.49796
H	-1.49218	-4.06605	0.90921

H	-2.55766	0.45381	-2.36662
H	-4.61946	-3.17823	-0.39609
H	1.00758	-3.90814	0.61512
H	0.98550	-2.54762	1.73556
H	2.10162	-2.49916	-1.11310
H	0.89353	4.75558	-2.59695
H	0.30206	6.72197	-1.19473
H	-0.40378	6.43357	1.16048
H	-0.55343	4.18150	2.17715
H	7.21941	0.51987	0.55827
H	8.13110	0.36810	-0.95172
H	6.36553	0.43100	-0.98796
H	8.09884	-1.82675	0.19556
H	7.19458	-1.93692	-1.30493
H	5.95765	-1.57276	1.48088
H	6.10426	-3.10542	0.63369
H	4.63910	-2.21622	-1.21499
H	4.43365	-0.71180	-0.32527
H	3.41954	-1.97609	1.59543
H	3.58181	-3.47607	0.68255
H	1.04849	-0.37562	-1.93010
H	-5.15267	-0.05239	-2.63557
H	0.31032	0.12729	2.00519
H	0.10121	1.67988	2.77182
H	-3.27762	-0.39617	4.69330
H	-3.95158	0.79996	3.53705
H	-3.61372	-0.86043	2.99267
H	0.95889	1.91303	-2.46880

Standard orientations of **1bc**

C	-1.17023	-3.24095	0.36624
C	-2.41389	-3.03648	-0.22519
C	-2.61974	-1.91325	-1.08587
C	-1.53703	-1.00797	-1.28064
C	-0.31605	-1.18404	-0.61446
C	-0.13119	-2.32985	0.17398
O	-1.59788	0.04523	-2.12263
O	-3.38020	-3.93433	0.03095
C	-3.93577	-1.75028	-1.68201
C	1.22071	-2.58982	0.80218
C	2.31422	-1.79322	0.09054
O	1.91317	-0.42051	0.06020
C	-0.34678	3.22844	0.35739
C	0.09095	3.45657	-0.97610
C	0.03822	4.71893	-1.57584
C	-0.46072	5.77065	-0.81574

C	-0.89392	5.57027	0.51141
C	-0.84104	4.31398	1.10161
C	7.70092	-0.96462	-1.26941
C	7.26953	-0.40108	0.08997
C	6.13469	-1.18212	0.77335
C	4.78854	-1.12597	0.03815
C	3.66995	-1.85935	0.78889
C	0.79799	-0.15233	-0.79401
O	-4.08318	-0.66397	-2.49716
O	-4.90043	-2.49641	-1.51431
C	0.37308	1.26613	-0.50404
C	-0.15599	1.82912	0.63544
C	-0.45746	1.13279	1.92522
C	-1.89371	0.63743	2.06145
O	-2.75773	0.67352	1.21389
O	-2.08632	0.11523	3.29690
C	-3.39162	-0.43384	3.53504
N	0.54377	2.24976	-1.46217
H	-1.03187	-4.12507	0.98020
H	-2.51333	0.16789	-2.43646
H	-4.19068	-3.65392	-0.45946
H	1.43961	-3.66455	0.77453
H	1.20721	-2.29683	1.86196
H	2.41676	-2.16205	-0.94556
H	0.37598	4.87374	-2.59755
H	-0.51609	6.76456	-1.25156
H	-1.27757	6.41463	1.07785
H	-1.18480	4.17277	2.12344
H	6.89078	-0.92253	-2.00612
H	8.54591	-0.39953	-1.67929
H	8.01424	-2.01267	-1.18041
H	6.96310	0.64789	-0.02932
H	8.13898	-0.38881	0.76098
H	5.99712	-0.78582	1.78950
H	6.44172	-2.23214	0.89583
H	4.89007	-1.56270	-0.96526
H	4.49006	-0.08017	-0.10592
H	3.55764	-1.43532	1.79641
H	3.93762	-2.91753	0.91986
H	1.14238	-0.19172	-1.84408
H	-5.01349	-0.66073	-2.79086
H	0.20055	0.26684	2.05847
H	-0.25400	1.78643	2.78325
H	-3.37599	-0.79175	4.56524
H	-4.16210	0.33121	3.40632
H	-3.59215	-1.25933	2.84632

H	0.79474	2.06392	-2.42113

Standard orientations of **1bd**

C	0.24560	-3.20393	0.60904
C	-0.87000	-3.54902	-0.14898
C	-1.39402	-2.62807	-1.10922
C	-0.76751	-1.35437	-1.23093
C	0.30532	-0.99215	-0.40428
C	0.83037	-1.94339	0.48347
O	-1.13519	-0.43577	-2.14911
O	-1.40399	-4.76625	0.04777
C	-2.55701	-3.04119	-1.87773
C	2.06035	-1.60222	1.29616
C	2.81761	-0.43027	0.67132
O	1.89455	0.64699	0.48853
C	-1.69080	3.01065	0.23082
C	-1.20426	3.37932	-1.05343
C	-1.69228	4.49051	-1.74845
C	-2.68542	5.24597	-1.13545
C	-3.17839	4.90443	0.14106
C	-2.69143	3.79860	0.82620
C	7.51519	-0.26094	-1.42331
C	6.68801	-0.06026	-0.14897
C	5.55185	0.95686	-0.32739
C	4.74805	1.26296	0.94867
C	3.95695	0.08699	1.54784
C	0.90551	0.40969	-0.51693
O	-3.02039	-2.12825	-2.78232
O	-3.13929	-4.12132	-1.77970
C	-0.10622	1.52160	-0.38781
C	-0.97657	1.82778	0.63401
C	-1.13876	1.09092	1.92633
C	-2.23628	0.03151	1.92364
O	-2.90649	-0.31801	0.97767
O	-2.36252	-0.50435	3.16186
C	-3.33434	-1.55482	3.28034
N	-0.22646	2.46885	-1.38921
H	0.64609	-3.93957	1.29911
H	-1.96212	-0.71401	-2.58541
H	-2.17673	-4.85903	-0.56071
H	2.70571	-2.48584	1.37704
H	1.77125	-1.32684	2.32078
H	3.20798	-0.74028	-0.31288
H	-1.30903	4.75575	-2.73065
H	-3.08728	6.11563	-1.64833
H	-3.95428	5.51735	0.59196

H	-3.08568	3.54323	1.80681
H	7.97503	0.68022	-1.74911
H	8.31968	-0.98917	-1.26931
H	6.89036	-0.62510	-2.24839
H	7.34586	0.27473	0.66600
H	6.28003	-1.02912	0.16973
H	4.87779	0.61077	-1.12439
H	5.98100	1.90005	-0.69447
H	4.04355	2.07640	0.74143
H	5.43846	1.63753	1.71718
H	3.52276	0.40403	2.50537
H	4.62469	-0.75505	1.77283
H	1.38363	0.50432	-1.50952
H	-3.81466	-2.52029	-3.19122
H	-0.20390	0.59152	2.20437
H	-1.35420	1.78303	2.75060
H	-3.31381	-1.85680	4.32822
H	-4.32898	-1.19411	3.00448
H	-3.06999	-2.39669	2.63432
H	0.20936	2.38826	-2.29514

Standard orientations of **1be**

C	-1.64914	-2.99479	0.42521
C	-2.89905	-2.69697	-0.10701
C	-3.04227	-1.59213	-0.99955
C	-1.88592	-0.80116	-1.28272
C	-0.65080	-1.06674	-0.67514
C	-0.53573	-2.19888	0.14184
O	-1.93200	0.21947	-2.15931
O	-3.91280	-3.51656	0.24809
C	-4.28970	-1.22215	-1.64682
C	0.82617	-2.58348	0.67109
C	1.92166	-2.00102	-0.22428
O	1.73110	-0.58529	-0.29747
C	0.03678	3.27246	0.47116
C	-0.08140	3.47226	-0.93059
C	-0.34711	4.72705	-1.48801
C	-0.48709	5.80038	-0.61566
C	-0.36257	5.62807	0.77861
C	-0.10282	4.37872	1.32697
C	7.05035	0.02003	-1.21294
C	6.98588	-1.49610	-0.99253
C	5.84714	-1.95661	-0.06724
C	4.43733	-1.71565	-0.62404
C	3.33408	-2.24274	0.30209
C	0.54473	-0.15816	-0.96875

O	-5.37099	-1.98835	-1.31563
O	-4.44444	-0.29979	-2.44830
C	0.31822	1.28092	-0.56296
C	0.29178	1.87122	0.68255
C	0.51691	1.19992	2.00457
C	-0.73781	0.58558	2.61410
O	-1.87550	0.96298	2.45463
O	-0.40846	-0.46200	3.41640
C	-1.51599	-1.09178	4.08046
N	0.13059	2.25070	-1.53422
H	-1.55791	-3.87223	1.05735
H	-2.86601	0.30368	-2.47966
H	-4.73832	-3.22471	-0.17954
H	0.90686	-3.67608	0.72682
H	0.95658	-2.19600	1.69085
H	1.83015	-2.43239	-1.23659
H	-0.43889	4.85939	-2.56310
H	-0.69512	6.78927	-1.01532
H	-0.47821	6.48848	1.43232
H	-0.02392	4.25752	2.40449
H	7.15329	0.55263	-0.25912
H	7.90882	0.29031	-1.83854
H	6.15018	0.40072	-1.70791
H	7.94048	-1.83483	-0.56767
H	6.88919	-2.00348	-1.96334
H	5.94466	-1.44929	0.90436
H	5.97053	-3.03019	0.13732
H	4.34894	-2.20350	-1.60689
H	4.26895	-0.64646	-0.79177
H	3.42000	-1.77010	1.29042
H	3.46048	-3.32354	0.45816
H	0.72805	-0.17514	-2.05774
H	-6.13002	-1.62728	-1.81124
H	1.28313	0.42656	1.92376
H	0.88921	1.92965	2.73760
H	-1.07776	-1.86367	4.71466
H	-2.06781	-0.36626	4.68398
H	-2.19570	-1.53701	3.34887
H	-0.13105	2.02594	-2.48261

Standard orientations of **1bf**

C	-2.33718	-2.82810	1.03594
C	-3.62825	-2.41199	0.72950
C	-3.82549	-1.32699	-0.17779
C	-2.66859	-0.68130	-0.71610
C	-1.37469	-1.06585	-0.33988

C	-1.22244	-2.17521	0.50377
O	-2.77450	0.31164	-1.61968
O	-4.63410	-3.10054	1.31196
C	-5.12871	-0.84514	-0.60334
C	0.16879	-2.69613	0.78519
C	1.12290	-2.27886	-0.33636
O	1.06957	-0.85484	-0.45225
C	0.09821	3.20845	0.30577
C	-0.24954	3.35602	-1.06399
C	-0.42431	4.60909	-1.66020
C	-0.23211	5.73123	-0.86240
C	0.13393	5.60844	0.49425
C	0.30584	4.36181	1.08170
C	6.12711	-0.90218	-2.42617
C	5.96622	-2.38575	-2.07343
C	4.98820	-2.66286	-0.91952
C	3.52679	-2.30516	-1.22272
C	2.57768	-2.65601	-0.07010
C	-0.16976	-0.32528	-0.92302
O	-6.20002	-1.47942	-0.04094
O	-5.33803	0.06408	-1.40763
C	-0.13874	1.14993	-0.60088
C	0.14955	1.79602	0.58093
C	0.44877	1.15504	1.90441
C	1.93273	1.06105	2.23136
O	2.76383	1.91018	2.00638
O	2.21494	-0.09939	2.88134
C	3.57905	-0.23511	3.31158
N	-0.34637	2.08801	-1.59819
H	-2.21843	-3.68404	1.69234
H	-3.73807	0.48481	-1.77059
H	-5.49215	-2.74023	1.02344
H	0.13983	-3.78745	0.89187
H	0.55156	-2.28760	1.73066
H	0.79143	-2.73517	-1.28586
H	-0.69506	4.70307	-2.70885
H	-0.35835	6.72047	-1.29423
H	0.29116	6.50705	1.08482
H	0.61134	4.27794	2.12095
H	6.45873	-0.32339	-1.55506
H	6.87138	-0.76523	-3.21908
H	5.18804	-0.46022	-2.77665
H	6.94896	-2.79689	-1.80541
H	5.63839	-2.94083	-2.96436
H	5.31829	-2.10959	-0.02716
H	5.04693	-3.72883	-0.65470

H	3.20608	-2.83700	-2.13168
H	3.43144	-1.23542	-1.43747
H	2.89603	-2.14178	0.84640
H	2.62157	-3.73468	0.13766
H	-0.20866	-0.42797	-2.02230
H	-6.99864	-1.05906	-0.41199
H	0.00049	1.75423	2.71087
H	0.00578	0.16069	1.97598
H	3.62796	-1.18908	3.83828
H	4.25539	-0.23522	2.45247
H	3.85617	0.58648	3.97766
H	-0.79228	1.86414	-2.47526

Standard orientations of **1bg**

C	-2.31777	-2.85403	0.99322
C	-3.60648	-2.42790	0.68346
C	-3.79910	-1.32997	-0.21440
C	-2.64221	-0.68569	-0.74498
C	-1.35270	-1.08154	-0.36849
C	-1.20337	-2.19855	0.47058
O	-2.70925	0.32095	-1.64871
O	-4.63174	-3.09470	1.23719
C	-5.16604	-0.94851	-0.53079
C	0.18699	-2.71611	0.76238
C	1.15168	-2.29484	-0.34762
O	1.09466	-0.87076	-0.46100
C	0.09534	3.19644	0.27292
C	-0.12748	3.33611	-1.12372
C	-0.21802	4.58580	-1.74568
C	-0.07049	5.71238	-0.94481
C	0.17111	5.59699	0.44021
C	0.26138	4.35405	1.05295
C	6.17511	-0.90706	-2.38627
C	6.01403	-2.39034	-2.03240
C	5.02452	-2.66765	-0.88836
C	3.56526	-2.31559	-1.20809
C	2.60486	-2.66636	-0.06486
C	-0.14097	-0.34429	-0.94438
O	-5.29874	0.09883	-1.40159
O	-6.18468	-1.47709	-0.08799
C	-0.10846	1.13245	-0.63153
C	0.09124	1.78618	0.56451
C	0.25921	1.15102	1.91551
C	1.70217	1.10159	2.39840
O	2.48553	2.02354	2.37094
O	2.00479	-0.11222	2.92708

C	3.33089	-0.22210	3.47034
N	-0.21019	2.06450	-1.65014
H	-2.20472	-3.71550	1.64327
H	-3.64156	0.55491	-1.81566
H	-5.47120	-2.68943	0.91237
H	0.15989	-3.80747	0.86820
H	0.55814	-2.30552	1.71154
H	0.83318	-2.75080	-1.30185
H	-0.39137	4.67372	-2.81537
H	-0.13338	6.69914	-1.39579
H	0.29803	6.49866	1.03336
H	0.47843	4.27356	2.11402
H	6.49681	-0.32604	-1.51289
H	6.92698	-0.76970	-3.17191
H	5.23852	-0.46795	-2.74702
H	6.99492	-2.79837	-1.75323
H	5.69717	-2.94785	-2.92577
H	5.34316	-2.11147	0.00623
H	5.08364	-3.73281	-0.62059
H	3.25608	-2.85096	-2.11899
H	3.46859	-1.24671	-1.42694
H	2.91173	-2.14795	0.85336
H	2.65055	-3.74421	0.14660
H	-0.17002	-0.45352	-2.04368
H	-6.25640	0.24994	-1.51190
H	-0.28912	1.73883	2.66617
H	-0.16046	0.14468	1.93491
H	3.40073	-1.23330	3.87312
H	4.07990	-0.06601	2.68942
H	3.48538	0.51798	4.26011
H	-0.54105	1.83931	-2.57601

Standard orientations of **1bh**

C	-1.49543	-3.18007	1.15153
C	-2.86615	-3.12189	0.91507
C	-3.39400	-2.15338	0.00236
C	-2.48058	-1.25154	-0.62026
C	-1.11355	-1.28060	-0.32085
C	-0.62471	-2.27947	0.53838
O	-2.86306	-0.33417	-1.54165
O	-3.64564	-4.00997	1.55265
C	-4.82802	-2.15861	-0.23347
C	0.86660	-2.39652	0.75094
C	1.61820	-1.80270	-0.44206
O	1.18415	-0.44771	-0.60586
C	-0.79596	3.23230	0.17008

C	-1.11942	3.27802	-1.21362
C	-1.55812	4.44828	-1.84157
C	-1.66274	5.59464	-1.06206
C	-1.33289	5.57674	0.30921
C	-0.89813	4.41177	0.92813
C	7.67793	-2.22044	-0.91494
C	6.16363	-2.41545	-1.04560
C	5.40767	-1.09697	-1.26316
C	3.89220	-1.24493	-1.47390
C	3.13253	-1.77272	-0.24877
C	-0.17636	-0.27842	-0.99785
O	-5.28266	-1.22172	-1.12239
O	-5.64457	-2.91610	0.28902
C	-0.50912	1.16476	-0.70225
C	-0.41977	1.87620	0.47499
C	-0.01063	1.34443	1.82140
C	1.48842	1.33319	2.09028
O	2.11599	0.37990	2.50688
O	2.04353	2.53786	1.85297
C	3.45831	2.62599	2.07901
N	-0.90289	2.01488	-1.72054
H	-1.12176	-3.95110	1.81729
H	-3.83154	-0.36391	-1.65518
H	-4.57915	-3.85388	1.27264
H	1.13609	-3.44922	0.89992
H	1.17232	-1.84643	1.65089
H	1.37181	-2.37410	-1.35477
H	-1.80381	4.46319	-2.90051
H	-1.99936	6.52147	-1.51871
H	-1.41694	6.49320	0.88719
H	-0.63208	4.41431	1.98191
H	8.09677	-1.75438	-1.81553
H	7.92142	-1.57151	-0.06452
H	8.19429	-3.17525	-0.76285
H	5.95292	-3.09361	-1.88551
H	5.78725	-2.91888	-0.14526
H	5.83564	-0.58787	-2.13876
H	5.59057	-0.43047	-0.40666
H	3.70954	-1.91063	-2.33146
H	3.47082	-0.26991	-1.74451
H	3.34773	-1.14691	0.62695
H	3.45898	-2.79181	-0.00308
H	-0.25640	-0.42503	-2.09033
H	-6.25052	-1.33423	-1.17353
H	-0.36094	0.32124	1.97082
H	-0.47208	1.95948	2.60609

H	3.69999	2.38074	3.11687
H	3.99473	1.94030	1.41748
H	3.72582	3.65911	1.85535
H	-1.20324	1.69158	-2.62761

Standard orientations of **1bi**

C	-0.23598	3.73676	1.10913
C	1.13369	3.95410	0.96967
C	1.93283	3.03210	0.22366
C	1.29056	1.89324	-0.33709
C	-0.06800	1.63749	-0.10307
C	-0.83353	2.59240	0.57963
O	1.88944	1.00804	-1.15096
O	1.65085	5.05791	1.53377
C	3.35466	3.31302	0.08372
C	-2.32927	2.39239	0.67922
C	-2.80803	1.45754	-0.43275
O	-2.06989	0.23503	-0.33249
C	0.97285	-2.81120	0.66529
C	0.11785	-2.38274	1.71643
C	-0.02557	-3.10250	2.90664
C	0.70935	-4.27502	3.04103
C	1.56648	-4.71854	2.01373
C	1.70605	-4.00037	0.83210
C	-8.20535	-1.35887	-0.33979
C	-6.71693	-0.99748	-0.29300
C	-6.28486	-0.09697	-1.45937
C	-4.78299	0.22895	-1.50301
C	-4.28950	1.10001	-0.33937
C	-0.66805	0.32875	-0.62477
O	4.06429	2.38427	-0.60930
O	3.93734	4.29539	0.54631
C	-0.06156	-0.90046	0.01996
C	0.84458	-1.85020	-0.40644
C	1.54411	-1.93337	-1.74017
C	3.06776	-1.91236	-1.63476
O	3.76805	-0.95097	-1.38270
O	3.58807	-3.13740	-1.87232
C	5.01977	-3.23330	-1.77737
N	-0.47053	-1.20729	1.30701
H	-0.82299	4.48156	1.63687
H	2.86443	1.00025	-1.09430
H	2.61699	5.07742	1.32528
H	-2.83779	3.36253	0.62089
H	-2.60011	1.95077	1.64942
H	-2.60453	1.92491	-1.41108

H	-0.68700	-2.75811	3.69746
H	0.62095	-4.85856	3.95346
H	2.12597	-5.64023	2.15024
H	2.36617	-4.35442	0.04617
H	-8.45484	-1.89091	-1.26615
H	-8.48815	-2.00292	0.50070
H	-8.83327	-0.46022	-0.29655
H	-6.11515	-1.91742	-0.30773
H	-6.49688	-0.50496	0.66356
H	-6.86259	0.83947	-1.42789
H	-6.56172	-0.58994	-2.40211
H	-4.56203	0.74820	-2.44668
H	-4.20199	-0.70123	-1.52076
H	-4.45671	0.59162	0.61906
H	-4.86534	2.03600	-0.30546
H	-0.51868	0.26756	-1.71285
H	4.99460	2.67698	-0.59860
H	1.26076	-2.85527	-2.26034
H	1.26015	-1.08311	-2.36380
H	5.25752	-4.27331	-2.00197
H	5.49656	-2.56465	-2.49873
H	5.35448	-2.96988	-0.77076
H	-1.28582	-0.77958	1.71952

Standard orientations of **1bj**

C	1.96563	-2.98254	-1.16283
C	3.28885	-2.74259	-0.80255
C	3.59328	-1.73692	0.16942
C	2.51221	-0.98701	0.72075
C	1.19372	-1.19261	0.29575
C	0.92831	-2.22634	-0.61799
O	2.68029	-0.05599	1.68996
O	4.23719	-3.49660	-1.38095
C	4.98838	-1.55167	0.53482
C	-0.50761	-2.54481	-0.96858
C	-1.44131	-2.07594	0.14883
O	-1.20973	-0.68068	0.35706
C	0.31181	3.26651	-0.08492
C	0.50409	3.28748	1.32303
C	0.72778	4.47356	2.03012
C	0.74708	5.65864	1.30379
C	0.53868	5.66332	-0.09139
C	0.31568	4.48335	-0.78886
C	-6.29619	-2.39228	2.95728
C	-6.32348	-1.53568	1.68565
C	-5.34367	-1.98889	0.59047

C	-3.86109	-1.84411	0.96034
C	-2.92144	-2.24198	-0.18482
C	0.06859	-0.34647	0.89696
O	5.22772	-0.58735	1.47584
O	5.94445	-2.17492	0.07591
C	0.22909	1.13950	0.68290
C	0.15118	1.88834	-0.47088
C	-0.04708	1.36669	-1.86609
C	-1.46318	1.54632	-2.39522
O	-2.10398	2.57227	-2.35410
O	-1.92138	0.40997	-2.98037
C	-3.23075	0.51522	-3.56384
N	0.41185	1.98450	1.76417
H	1.76316	-3.77985	-1.87047
H	3.62968	0.04734	1.88931
H	5.11186	-3.22150	-1.01525
H	-0.61406	-3.62198	-1.14569
H	-0.79936	-2.03144	-1.89516
H	-1.20795	-2.62804	1.07658
H	0.87540	4.47040	3.10725
H	0.91655	6.59882	1.82186
H	0.54353	6.60989	-0.62517
H	0.12398	4.49846	-1.85764
H	-5.32325	-2.34879	3.45955
H	-7.05112	-2.05337	3.67599
H	-6.50471	-3.44522	2.72831
H	-6.11320	-0.48846	1.94519
H	-7.34078	-1.54754	1.27168
H	-5.53758	-1.40262	-0.31924
H	-5.55533	-3.03671	0.32736
H	-3.63182	-2.46091	1.84063
H	-3.65172	-0.80538	1.24423
H	-3.13630	-1.63375	-1.07360
H	-3.09572	-3.29050	-0.46557
H	0.05459	-0.52838	1.98694
H	6.19357	-0.56535	1.61293
H	0.60482	1.91932	-2.55829
H	0.23304	0.31535	-1.93815
H	-3.43394	-0.46005	-4.00788
H	-3.97424	0.75233	-2.79822
H	-3.24950	1.29708	-4.32780
H	0.67944	1.66114	2.68135

Standard orientations of **1bk**

C	-0.78216	3.53269	1.19955
C	0.51546	3.99213	0.98084

C	1.42346	3.22289	0.18770
C	0.96593	1.98212	-0.33676
C	-0.30819	1.48756	-0.02395
C	-1.19293	2.29472	0.70389
O	1.66635	1.21252	-1.18635
O	0.85664	5.17592	1.51590
C	2.76084	3.75417	-0.03440
C	-2.61988	1.82934	0.89028
C	-2.98486	0.81184	-0.19184
O	-2.03266	-0.25555	-0.13411
C	1.56405	-2.69379	0.66976
C	0.71079	-2.41707	1.77199
C	0.77158	-3.14039	2.96711
C	1.71252	-4.16024	3.05383
C	2.57195	-4.45121	1.97522
C	2.50825	-3.73001	0.78891
C	-8.65709	-1.32591	-0.80949
C	-7.33785	-0.55853	-0.94725
C	-6.10806	-1.47773	-0.93342
C	-4.76250	-0.76523	-1.14433
C	-4.37038	0.19528	-0.01284
C	-0.69116	0.08723	-0.51127
O	3.58515	2.96239	-0.76926
O	3.18249	4.82941	0.39524
C	0.16520	-1.00628	0.09294
C	1.20033	-1.78094	-0.38992
C	1.82235	-1.74740	-1.76363
C	3.32086	-1.45197	-1.74975
O	3.85046	-0.37864	-1.53604
O	4.03797	-2.56500	-2.02328
C	5.46662	-2.40119	-2.01481
N	-0.10337	-1.37031	1.40187
H	-1.46243	4.16417	1.76195
H	2.62841	1.38065	-1.18668
H	1.78961	5.36762	1.25120
H	-3.29760	2.69136	0.86166
H	-2.75066	1.35628	1.87455
H	-2.92521	1.29798	-1.18029
H	0.10782	-2.91379	3.79753
H	1.78595	-4.74214	3.96863
H	3.29553	-5.25596	2.07525
H	3.17261	-3.96640	-0.03666
H	-8.69180	-1.88814	0.13191
H	-9.51875	-0.64875	-0.82450
H	-8.78388	-2.04550	-1.62773
H	-7.26509	0.17870	-0.13638

H	-7.34561	0.01798	-1.88378
H	-6.22825	-2.23588	-1.72027
H	-6.08038	-2.03190	0.01690
H	-4.79077	-0.21119	-2.09475
H	-3.96987	-1.51496	-1.24697
H	-4.38885	-0.33781	0.94867
H	-5.09580	1.01525	0.06691
H	-0.59900	0.04422	-1.60656
H	4.44641	3.41805	-0.81255
H	1.67875	-2.70899	-2.26902
H	1.35349	-0.96647	-2.36602
H	5.87375	-3.38309	-2.25710
H	5.77140	-1.66287	-2.76098
H	5.80796	-2.07464	-1.02914
H	-0.95604	-1.09270	1.86416

Standard orientations of **1bl**

C	2.57770	-2.33747	-1.43001
C	3.78295	-1.85316	-0.93318
C	3.78041	-0.91859	0.14706
C	2.51660	-0.48899	0.66045
C	1.31187	-0.93597	0.10329
C	1.35481	-1.89705	-0.91694
O	2.43124	0.35099	1.71009
O	4.90593	-2.33307	-1.51126
C	4.97964	-0.38572	0.76966
C	0.05821	-2.49142	-1.41569
C	-1.01821	-2.38111	-0.33282
O	-1.14474	-1.00022	0.02615
C	-0.55133	3.21032	-0.06190
C	-0.41704	3.17192	1.35276
C	-0.48814	4.32383	2.14271
C	-0.70986	5.53385	1.49510
C	-0.86262	5.59405	0.09424
C	-0.78979	4.44719	-0.68594
C	-4.69710	-2.45696	3.19966
C	-4.52196	-3.76136	2.41265
C	-3.34219	-3.75520	1.42567
C	-3.50149	-2.76940	0.25805
C	-2.39335	-2.85188	-0.80475
C	-0.01112	-0.42760	0.67576
O	6.15998	-0.81042	0.22765
O	5.01673	0.39691	1.72057
C	-0.18399	1.06869	0.56657
C	-0.38827	1.86240	-0.54159
C	-0.42407	1.41638	-1.97735

C	-1.76856	0.90505	-2.47702
O	-1.93199	-0.11975	-3.10935
O	-2.76762	1.75764	-2.17810
C	-4.07569	1.36050	-2.61546
N	-0.23177	1.85238	1.70634
H	2.61202	-3.07811	-2.22232
H	3.35259	0.59196	1.98203
H	5.69005	-1.94241	-1.08499
H	0.21855	-3.53749	-1.70419
H	-0.29843	-1.95501	-2.30515
H	-0.70075	-2.95615	0.55324
H	-0.37808	4.27531	3.22312
H	-0.77131	6.44815	2.07915
H	-1.04428	6.55536	-0.37899
H	-0.92255	4.50515	-1.76322
H	-3.78798	-2.21243	3.76369
H	-4.91521	-1.60852	2.54188
H	-5.52146	-2.53779	3.91761
H	-4.38276	-4.58863	3.12190
H	-5.44862	-3.98443	1.86423
H	-3.21808	-4.76949	1.01835
H	-2.41897	-3.53480	1.98083
H	-3.54598	-1.74064	0.63275
H	-4.46461	-2.96487	-0.23487
H	-2.66948	-2.24098	-1.67232
H	-2.29402	-3.88768	-1.16000
H	-0.03949	-0.69169	1.74827
H	6.87100	-0.37851	0.73752
H	0.29988	0.62149	-2.16831
H	-0.14591	2.26155	-2.62199
H	-4.74241	2.16843	-2.31265
H	-4.09723	1.22853	-3.70074
H	-4.37019	0.42239	-2.13722
H	0.09557	1.53428	2.60627

Standard orientations of **1bm**

C	-1.33414	-3.14730	1.27028
C	-2.69666	-3.17072	0.99276
C	-3.23981	-2.26672	0.02940
C	-2.35008	-1.33899	-0.59756
C	-0.99273	-1.28466	-0.25665
C	-0.48566	-2.22608	0.65047
O	-2.77609	-0.48950	-1.55225
O	-3.42857	-4.09051	1.65902
C	-4.63677	-2.23728	-0.36719
C	1.00306	-2.26662	0.90569

C	1.75594	-1.68014	-0.29107
O	1.26735	-0.35208	-0.51154
C	-0.90478	3.25006	0.10437
C	-1.24594	3.22409	-1.27536
C	-1.76373	4.34086	-1.93922
C	-1.92939	5.50709	-1.20086
C	-1.58218	5.56107	0.16512
C	-1.06954	4.44865	0.82013
C	6.39348	-3.14201	-1.79919
C	6.35440	-2.07530	-0.69843
C	5.52604	-0.82763	-1.05349
C	4.02472	-1.05963	-1.29618
C	3.26321	-1.57872	-0.06879
C	-0.08959	-0.25887	-0.94105
O	-5.44820	-3.12803	0.27738
O	-5.13472	-1.49397	-1.21433
C	-0.50256	1.17292	-0.69572
C	-0.44488	1.93103	0.45394
C	-0.00038	1.47334	1.81586
C	1.49864	1.53506	2.07627
O	2.15965	0.63558	2.55653
O	2.01009	2.73936	1.75523
C	3.42037	2.89388	1.97236
N	-0.95559	1.95928	-1.74061
H	-0.94353	-3.87378	1.97536
H	-3.74913	-0.63015	-1.67195
H	-4.36169	-4.03210	1.38506
H	1.31639	-3.30002	1.09825
H	1.26197	-1.67274	1.79241
H	1.55421	-2.29264	-1.18793
H	-2.02362	4.30054	-2.99401
H	-2.32867	6.39360	-1.68617
H	-1.71552	6.49174	0.71022
H	-0.79266	4.50679	1.86960
H	5.40010	-3.55888	-2.00136
H	6.77337	-2.72446	-2.74028
H	7.04730	-3.97550	-1.51756
H	5.97657	-2.52247	0.23091
H	7.38152	-1.75448	-0.47786
H	5.95783	-0.36465	-1.95260
H	5.64076	-0.08911	-0.24682
H	3.88425	-1.75655	-2.13494
H	3.56888	-0.11293	-1.60823
H	3.43422	-0.91728	0.79009
H	3.62686	-2.57493	0.21674
H	-0.13570	-0.44030	-2.03000

H	-6.34528	-2.99823	-0.08383
H	-0.30666	0.44376	2.01187
H	-0.48207	2.10003	2.57906
H	3.67089	2.72375	3.02309
H	3.98215	2.18819	1.35421
H	3.64956	3.91994	1.68319
H	-1.27549	1.57690	-2.61790

Standard orientations of **1bn**

C	2.58194	-2.35276	-1.41345
C	3.78425	-1.85750	-0.91614
C	3.77837	-0.91315	0.15990
C	2.51634	-0.48824	0.67177
C	1.31590	-0.94582	0.11600
C	1.36094	-1.91295	-0.90270
O	2.39443	0.35717	1.72401
O	4.92044	-2.31381	-1.46723
C	5.06227	-0.45462	0.66331
C	0.06596	-2.50763	-1.40509
C	-1.01704	-2.39616	-0.32997
O	-1.14254	-1.01518	0.02889
C	-0.56033	3.19622	-0.03881
C	-0.48951	3.14421	1.38021
C	-0.61289	4.28618	2.17832
C	-0.82117	5.50032	1.53396
C	-0.90979	5.57409	0.12818
C	-0.78611	4.43703	-0.65987
C	-4.71746	-2.47596	3.17766
C	-4.54803	-3.77599	2.38222
C	-3.36191	-3.77245	1.40287
C	-3.50643	-2.77822	0.24049
C	-2.39030	-2.86028	-0.81386
C	-0.01264	-0.44373	0.68521
O	5.00428	0.43850	1.69946
O	6.16749	-0.79429	0.24305
C	-0.19424	1.05204	0.58294
C	-0.36025	1.85557	-0.52467
C	-0.33750	1.42210	-1.96494
C	-1.66706	0.92609	-2.51805
O	-1.82455	-0.11040	-3.13215
O	-2.66017	1.80642	-2.28543
C	-3.95572	1.42492	-2.77183
N	-0.29971	1.82386	1.72656
H	2.62045	-3.09747	-2.20167
H	3.27782	0.64969	2.01690
H	5.67901	-1.88645	-1.00233

H	0.22767	-3.55380	-1.69212
H	-0.28442	-1.97058	-2.29662
H	-0.70873	-2.97315	0.55814
H	-0.55308	4.22708	3.26222
H	-0.92270	6.40708	2.12416
H	-1.08264	6.53814	-0.34274
H	-0.87146	4.50472	-1.74127
H	-4.92356	-1.62083	2.52465
H	-5.54751	-2.55486	3.88922
H	-3.81020	-2.24368	3.74990
H	-5.47295	-3.98725	1.82630
H	-4.42035	-4.60951	3.08627
H	-3.24278	-4.78501	0.98991
H	-2.44056	-3.56269	1.96527
H	-3.54828	-1.75152	0.62108
H	-4.46669	-2.96512	-0.26119
H	-2.65704	-2.24394	-1.68070
H	-2.29232	-3.89461	-1.17361
H	-0.04505	-0.71357	1.75645
H	5.92678	0.66327	1.92415
H	-0.02698	2.27006	-2.59063
H	0.38634	0.62175	-2.13090
H	-4.61900	2.25119	-2.51440
H	-3.93248	1.27147	-3.85424
H	-4.28885	0.50227	-2.28896
H	-0.04305	1.49326	2.64435

Standard orientations of **2aa**

C	1.35760	2.95650	-0.37260
C	0.27510	3.55470	-1.00760
C	-1.02470	3.12420	-0.73900
C	-1.21460	2.07020	0.18170
C	-0.12620	1.43080	0.82900
C	1.18200	1.90550	0.54980
C	-2.21040	3.73290	-1.38850
O	-1.92170	4.73200	-2.24630
O	-3.36360	3.38850	-1.19120
O	-2.47950	1.62380	0.48670
O	0.59050	4.56370	-1.87740
C	2.45090	1.34990	1.18080
C	3.13230	0.26640	0.32250
O	2.55020	-1.00720	0.59260
C	4.62770	0.17650	0.66300
C	5.35990	-0.85400	-0.20450
C	6.85370	-0.89410	0.12400
C	7.58570	-1.92020	-0.74060

C	9.07070	-1.96290	-0.41750
O	-4.72200	-1.17960	1.86300
C	-2.80920	-5.41040	-1.20710
C	-2.89860	-4.41210	-3.49990
C	-3.58970	-1.36310	1.42930
C	-2.30140	-1.08910	2.21130
N	-1.21790	-0.77600	1.28900
C	-0.96220	-1.54500	0.17220
C	-2.08620	-2.57410	-0.13260
N	-3.38860	-2.00450	0.22390
C	-2.37540	0.11150	3.14300
C	-0.94970	0.66630	3.14910
C	-0.34310	0.26740	1.79310
O	0.10000	-1.48960	-0.44470
C	-2.03260	-3.00960	-1.60360
C	-3.02530	-4.12180	-2.00140
H	2.35830	3.32090	-0.60290
H	-2.80450	5.01360	-2.57840
H	-3.15280	2.11380	-0.03330
H	-0.24520	4.90350	-2.26090
H	3.13770	2.19700	1.31710
H	2.25260	0.98830	2.19550
H	3.02120	0.47670	-0.74810
H	1.71640	-1.06980	0.07340
H	5.09570	1.15900	0.52670
H	4.74730	-0.10460	1.71710
H	4.92750	-1.84960	-0.04940
H	5.22400	-0.60320	-1.26350
H	7.29180	0.09880	-0.03440
H	6.99030	-1.14220	1.18370
H	7.15460	-2.91570	-0.58280
H	7.45540	-1.67450	-1.80100
H	9.57440	-2.70300	-1.04710
H	9.53920	-0.98980	-0.59600
H	9.23670	-2.23780	0.62900
H	-3.46940	-6.20560	-1.56990
H	-1.77610	-5.76300	-1.29550
H	-3.03230	-5.26620	-0.14580
H	-3.62470	-5.17030	-3.81220
H	-3.08790	-3.50830	-4.08870
H	-1.89730	-4.77810	-3.75140
H	-2.02310	-1.99900	2.75680
H	-1.90850	-3.42540	0.53460
H	-4.23990	-2.26210	-0.27830
H	-2.70780	-0.17120	4.14720
H	-3.07800	0.86850	2.77910

H	-0.38660	0.17360	3.95220
H	-0.92360	1.74230	3.34880
H	0.60340	-0.23890	1.98790
H	-1.01520	-3.34830	-1.84130
H	-2.21440	-2.13080	-2.23760
H	-4.04990	-3.77650	-1.82160

Standard orientations of **2ab**

C	1.35510	2.95900	-0.38900
C	0.27040	3.55130	-1.02480
C	-1.03050	3.11960	-0.75440
C	-1.21800	2.07010	0.16900
C	-0.12670	1.43610	0.81850
C	1.18120	1.91210	0.53760
C	-2.14450	3.79680	-1.46160
O	-3.36680	3.31940	-1.15620
O	-2.00800	4.70990	-2.25890
O	-2.47820	1.62060	0.47770
O	0.57990	4.56000	-1.90050
C	2.45110	1.36260	1.17200
C	3.13510	0.27540	0.32030
O	2.55770	-0.99820	0.60070
C	4.63130	0.19310	0.65900
C	5.36560	-0.84110	-0.20220
C	6.86010	-0.87380	0.12420
C	7.59420	-1.90360	-0.73420
C	9.07980	-1.93890	-0.41320
O	-4.72200	-1.13960	1.83470
C	-2.80350	-5.42480	-1.16690
C	-2.87360	-4.45280	-3.47170
C	-3.58720	-1.34220	1.41640
C	-2.30340	-1.07380	2.20830
N	-1.21330	-0.77080	1.29020
C	-0.95350	-1.54900	0.18050
C	-2.07800	-2.57880	-0.12040
N	-3.38070	-1.99940	0.22010
C	-2.37540	0.12990	3.13620
C	-0.94820	0.68110	3.14270
C	-0.34030	0.27680	1.78890
O	0.11070	-1.49940	-0.43290
C	-2.01560	-3.03160	-1.58570
C	-3.00950	-4.14470	-1.97750
H	2.35440	3.32530	-0.62170
H	-3.97520	3.86840	-1.70170
H	-3.13480	2.11790	-0.05350
H	-0.24690	4.91230	-2.29570

H	3.13610	2.21180	1.30360
H	2.25330	1.00660	2.18880
H	3.02150	0.47770	-0.75160
H	1.72550	-1.06890	0.08020
H	5.09570	1.17620	0.51480
H	4.75350	-0.07990	1.71500
H	4.93690	-1.83700	-0.03920
H	5.22720	-0.59840	-1.26270
H	7.29450	0.11950	-0.04210
H	6.99920	-1.11370	1.18540
H	7.16680	-2.89930	-0.56850
H	7.46130	-1.66600	-1.79610
H	9.58510	-2.68180	-1.03820
H	9.54470	-0.96550	-0.59940
H	9.24850	-2.20560	0.63500
H	-3.46410	-6.22190	-1.52500
H	-1.77110	-5.78210	-1.24410
H	-3.03340	-5.26760	-0.10890
H	-3.60020	-5.21210	-3.78020
H	-3.05560	-3.55520	-4.07220
H	-1.87190	-4.82520	-3.71200
H	-2.03430	-1.98390	2.75810
H	-1.90820	-3.42270	0.55800
H	-4.22900	-2.25880	-0.28610
H	-2.70930	-0.14940	4.14090
H	-3.07590	0.88770	2.77030
H	-0.38780	0.18900	3.94810
H	-0.91940	1.75760	3.33950
H	0.60740	-0.22630	1.98610
H	-0.99780	-3.37650	-1.81220
H	-2.18970	-2.15960	-2.23120
H	-4.03410	-3.79380	-1.80880

Standard orientations of **2ac**

C	-0.98290	2.84210	-0.10530
C	0.17250	3.39310	0.43740
C	1.40940	2.78300	0.22680
C	1.46020	1.59800	-0.53960
C	0.29340	1.00200	-1.08280
C	-0.94630	1.65950	-0.87090
C	2.66860	3.33160	0.78480
O	2.51280	4.46710	1.49420
O	3.77000	2.82870	0.63800
O	2.66040	0.97250	-0.78710
O	-0.00930	4.54150	1.15990
C	-2.28140	1.17390	-1.41710

C	-3.07380	0.31450	-0.41280
O	-2.66020	-1.04720	-0.50500
C	-4.57580	0.36740	-0.73140
C	-5.41120	-0.43300	0.27450
C	-6.90520	-0.32970	-0.03910
C	-7.74000	-1.12620	0.96350
C	-9.22540	-1.02580	0.65560
O	4.51620	-2.33890	-1.74400
C	2.36630	-2.64140	4.37770
C	1.96550	-0.77440	2.76710
C	3.38700	-2.28580	-1.26820
C	2.12310	-1.94860	-2.06570
N	1.11120	-1.35940	-1.19930
C	0.77510	-1.91710	0.01660
C	1.79060	-2.98690	0.49800
N	3.14520	-2.70330	0.02380
C	2.33330	-0.92050	-3.16740
C	0.99040	-0.19310	-3.25930
C	0.35820	-0.30890	-1.86180
O	-0.27750	-1.66310	0.59860
C	1.74140	-3.22630	2.01540
C	2.47840	-2.20550	2.91260
H	-1.93090	3.34850	0.07400
H	3.42780	4.68610	1.78400
H	3.39430	1.44610	-0.33890
H	0.86700	4.83270	1.48900
H	-2.86440	2.06870	-1.67620
H	-2.14280	0.64890	-2.36830
H	-2.91450	0.65490	0.61750
H	-1.83210	-1.14240	0.01840
H	-4.91520	1.41030	-0.73180
H	-4.75200	-0.03920	-1.73550
H	-5.10920	-1.48690	0.25830
H	-5.22240	-0.05740	1.28750
H	-7.21320	0.72280	-0.01910
H	-7.09440	-0.70260	-1.05310
H	-7.43930	-2.18020	0.94460
H	-7.55730	-0.75470	1.97860
H	-9.80340	-1.60250	1.38460
H	-9.56560	0.01380	0.69830
H	-9.44690	-1.41950	-0.34150
H	2.92780	-1.96390	5.02990
H	1.32320	-2.64650	4.71210
H	2.77300	-3.64870	4.51700
H	2.46200	-0.10940	3.48230
H	2.17410	-0.37900	1.77010

H	0.88750	-0.71530	2.94910
H	1.71030	-2.88140	-2.46940
H	1.48350	-3.91610	-0.00140
H	3.96540	-3.02540	0.54130
H	2.60910	-1.39030	-4.11720
H	3.13340	-0.21530	-2.91940
H	0.35620	-0.71980	-3.98400
H	1.09980	0.83680	-3.61390
H	-0.64390	-0.72310	-1.98310
H	2.19300	-4.21050	2.20380
H	0.69620	-3.30860	2.34040
H	3.54460	-2.21000	2.65520

Standard orientations of **2ad**

C	-0.97310	2.82440	-0.18860
C	0.18810	3.37490	0.34090
C	1.41990	2.74290	0.15420
C	1.45870	1.53740	-0.57630
C	0.28460	0.94230	-1.10790
C	-0.94830	1.61980	-0.91880
C	2.62160	3.38180	0.74340
O	3.76680	2.70110	0.54160
O	2.61560	4.43010	1.36720
O	2.64810	0.88950	-0.80330
O	0.02260	4.54890	1.02960
C	-2.28780	1.13200	-1.45210
C	-3.08910	0.31020	-0.42340
O	-2.69060	-1.05820	-0.47650
C	-4.59050	0.37040	-0.74330
C	-5.43430	-0.39170	0.28520
C	-6.92720	-0.28100	-0.03110
C	-7.77030	-1.03920	0.99390
C	-9.25470	-0.93130	0.68330
O	4.49800	-2.38840	-1.67150
C	3.23070	-1.03400	2.70370
C	1.79360	-2.08620	4.45520
C	3.36870	-2.32800	-1.19650
C	2.10070	-2.03570	-2.00430
N	1.08560	-1.42350	-1.15840
C	0.75270	-1.93640	0.07650
C	1.78700	-2.95800	0.61420
N	3.13560	-2.68930	0.11300
C	2.30190	-1.04630	-3.14320
C	0.95790	-0.32370	-3.25290
C	0.33360	-0.39190	-1.84950
O	-0.30900	-1.67950	0.64060

C	1.75570	-3.07400	2.14650
C	1.91530	-1.76760	2.96050
H	-1.91470	3.34820	-0.02670
H	4.44800	3.25180	0.99080
H	3.36990	1.37960	-0.35660
H	0.89400	4.85780	1.35980
H	-2.86160	2.02460	-1.73780
H	-2.15350	0.57770	-2.38720
H	-2.92570	0.67820	0.59670
H	-1.86420	-1.14890	0.05020
H	-4.91850	1.41660	-0.77350
H	-4.77160	-0.06280	-1.73530
H	-5.14390	-1.44880	0.29910
H	-5.24100	0.01060	1.28700
H	-7.22360	0.77500	-0.04130
H	-7.12080	-0.68060	-1.03410
H	-7.48130	-2.09650	1.00500
H	-7.58320	-0.64090	1.99790
H	-9.83870	-1.48050	1.42850
H	-9.58340	0.11280	0.69620
H	-9.48080	-1.35090	-0.30220
H	3.34660	-0.19050	3.39330
H	4.09080	-1.69800	2.83860
H	3.26420	-0.61920	1.69260
H	1.84670	-1.17100	5.05460
H	0.83570	-2.57000	4.67390
H	2.59580	-2.75480	4.78560
H	1.69670	-2.98700	-2.37150
H	1.50370	-3.92980	0.18860
H	3.96010	-2.98100	0.64140
H	2.57280	-1.54900	-4.07740
H	3.10210	-0.33100	-2.92570
H	0.32070	-0.87610	-3.95560
H	1.06380	0.69350	-3.64340
H	-0.67200	-0.80280	-1.95080
H	2.52800	-3.78840	2.46230
H	0.79280	-3.52370	2.42550
H	1.09640	-1.08340	2.71060

Standard orientations of **2ae**

C	-0.97370	2.81910	-0.22120
C	0.18670	3.37770	0.30280
C	1.41680	2.74450	0.12220
C	1.45590	1.52940	-0.59600
C	0.28340	0.92680	-1.12050
C	-0.94910	1.60590	-0.93800

C	2.68060	3.29940	0.66340
O	2.53670	4.46590	1.32340
O	3.77580	2.77610	0.54390
O	2.64960	0.88120	-0.81460
O	0.01660	4.55650	0.97760
C	-2.28880	1.11070	-1.46370
C	-3.08840	0.30150	-0.42380
O	-2.68650	-1.06640	-0.45680
C	-4.58980	0.35350	-0.74500
C	-5.43220	-0.39580	0.29400
C	-6.92530	-0.29330	-0.02450
C	-7.76690	-1.03860	1.01100
C	-9.25150	-0.93890	0.69840
O	4.48740	-2.44940	-1.66810
C	3.26160	-1.00160	2.69630
C	1.82560	-2.01160	4.47330
C	3.36370	-2.36380	-1.18360
C	2.09350	-2.06780	-1.98590
N	1.08640	-1.43880	-1.14290
C	0.75780	-1.93280	0.10080
C	1.79060	-2.95050	0.64850
N	3.13710	-2.69990	0.13350
C	2.29800	-1.09060	-3.13470
C	0.95680	-0.36380	-3.25040
C	0.33370	-0.41580	-1.84570
O	-0.30060	-1.66420	0.66590
C	1.76900	-3.03990	2.18280
C	1.94210	-1.72060	2.97260
H	-1.91620	3.34310	-0.06520
H	3.45310	4.68520	1.60810
H	3.38760	1.36340	-0.38270
H	0.89540	4.85100	1.29690
H	-2.86360	1.99920	-1.76010
H	-2.15530	0.54460	-2.39180
H	-2.92640	0.68460	0.59100
H	-1.85790	-1.14690	0.06820
H	-4.92030	1.39820	-0.79050
H	-4.76940	-0.09450	-1.73070
H	-5.13930	-1.45190	0.32330
H	-5.24030	0.02140	1.29000
H	-7.22420	0.76170	-0.05000
H	-7.11750	-0.70780	-1.02160
H	-7.47530	-2.09500	1.03760
H	-7.58120	-0.62540	2.00930
H	-9.83440	-1.47870	1.45120
H	-9.58270	0.10440	0.69610

H	-9.47620	-1.37320	-0.28100
H	3.38720	-0.14670	3.37010
H	4.11740	-1.66960	2.83890
H	3.29320	-0.60530	1.67780
H	1.88870	-1.08630	5.05600
H	0.86520	-2.48410	4.70540
H	2.62450	-2.68030	4.81160
H	1.67970	-3.01890	-2.34250
H	1.49840	-3.92780	0.24190
H	3.96380	-2.98770	0.66090
H	2.56660	-1.60320	-4.06410
H	3.10090	-0.37630	-2.92370
H	0.31660	-0.92010	-3.94720
H	1.06680	0.64920	-3.65070
H	-0.67150	-0.82890	-1.94170
H	2.53900	-3.75350	2.50590
H	0.80530	-3.47870	2.47600
H	1.12700	-1.03480	2.71450

Standard orientations of **2af**

C	1.50400	2.79870	-0.79950
C	0.37250	3.58740	-0.97110
C	-0.83480	3.23480	-0.36280
C	-0.88200	2.06310	0.42100
C	0.25400	1.23070	0.59800
C	1.47070	1.62770	-0.01700
C	-2.00340	4.12170	-0.58080
O	-3.13240	3.71110	0.02920
O	-1.98570	5.14250	-1.24840
O	-2.04190	1.68290	1.04990
O	0.54340	4.69990	-1.75410
C	2.78190	0.86290	0.10010
C	3.01650	-0.12170	-1.06140
O	2.38650	-1.37260	-0.79220
C	4.51070	-0.39460	-1.29990
C	5.23610	-1.01210	-0.09770
C	6.70330	-1.29930	-0.42610
C	7.43120	-1.91150	0.77040
C	8.88910	-2.19840	0.44820
O	-4.13740	-1.04620	2.66980
C	-3.83130	-4.91680	-1.32780
C	-4.45030	-3.56410	-3.33990
C	-3.22060	-1.27900	1.88940
C	-1.73400	-1.26920	2.26020
N	-0.93820	-0.91640	1.09180
C	-1.13460	-1.51600	-0.13540

C	-2.43130	-2.37060	-0.19740
N	-3.47680	-1.73930	0.61340
C	-1.35360	-0.24010	3.31440
C	0.07280	0.16700	2.93930
C	0.18200	-0.05350	1.42100
O	-0.30610	-1.46270	-1.04210
C	-2.87510	-2.57080	-1.65300
C	-4.08750	-3.50380	-1.85300
H	2.42870	3.10760	-1.28570
H	-3.79330	4.39870	-0.21500
H	-2.75120	2.32160	0.82740
H	-0.30610	5.18830	-1.81460
H	3.58850	1.60910	0.11440
H	2.84550	0.36200	1.07180
H	2.59700	0.26900	-1.99670
H	1.43250	-1.27960	-1.01580
H	4.60380	-1.08610	-2.14750
H	5.00840	0.53880	-1.58990
H	5.18730	-0.33140	0.75970
H	4.74150	-1.94440	0.19940
H	6.76150	-1.98410	-1.28090
H	7.20220	-0.36860	-0.72280
H	7.37980	-1.22910	1.62680
H	6.93860	-2.84430	1.06870
H	9.39040	-2.63560	1.31740
H	8.97540	-2.90390	-0.38430
H	9.41900	-1.27970	0.17690
H	-4.67450	-5.57640	-1.56010
H	-2.93070	-5.34990	-1.77590
H	-3.70920	-4.92150	-0.24060
H	-5.33550	-4.18880	-3.49960
H	-4.67490	-2.56430	-3.72640
H	-3.62960	-3.98140	-3.93340
H	-1.44380	-2.28070	2.56940
H	-2.18710	-3.32980	0.27330
H	-4.46540	-1.82920	0.37340
H	-1.40660	-0.65460	4.32640
H	-2.01710	0.63060	3.29490
H	0.77350	-0.50600	3.45010
H	0.31170	1.18630	3.25900
H	1.06470	-0.66730	1.23550
H	-2.03130	-2.96500	-2.23500
H	-3.10960	-1.58920	-2.08770
H	-4.95430	-3.09320	-1.32230

Standard orientations of **2ag**

C	1.50150	2.80260	-0.78010
C	0.37040	3.59340	-0.94780
C	-0.83440	3.23690	-0.34100
C	-0.88080	2.05940	0.43750
C	0.25380	1.22550	0.60940
C	1.46940	1.62640	-0.00450
C	-2.06580	4.04950	-0.48810
O	-1.91490	5.15370	-1.24660
O	-3.14200	3.77710	0.01690
O	-2.04400	1.67810	1.06520
O	0.54430	4.70720	-1.72460
C	2.78020	0.86020	0.10570
C	3.01280	-0.11600	-1.06340
O	2.37970	-1.36730	-0.80460
C	4.50640	-0.39050	-1.30380
C	5.23000	-1.01910	-0.10630
C	6.69650	-1.30740	-0.43680
C	7.42280	-1.93060	0.75510
C	8.88000	-2.21870	0.43090
O	-4.12790	-1.10730	2.69240
C	-3.83220	-4.90170	-1.36980
C	-4.46840	-3.52060	-3.35710
C	-3.21560	-1.31370	1.89940
C	-1.72670	-1.29340	2.25890
N	-0.93920	-0.92400	1.09000
C	-1.13970	-1.51070	-0.14260
C	-2.43430	-2.36790	-0.20900
N	-3.47720	-1.75430	0.61790
C	-1.34950	-0.26910	3.31890
C	0.07410	0.14720	2.94380
C	0.18130	-0.06350	1.42400
O	-0.31440	-1.44620	-1.05170
C	-2.88670	-2.54740	-1.66480
C	-4.09680	-3.48180	-1.87170
H	2.42640	3.11320	-1.26500
H	-2.80960	5.56410	-1.23780
H	-2.76890	2.30740	0.85970
H	-0.31350	5.17950	-1.77090
H	3.58730	1.60580	0.12450
H	2.84460	0.35200	1.07350
H	2.59450	0.28320	-1.99570
H	1.42520	-1.26930	-1.02410
H	4.59800	-1.07540	-2.15670
H	5.00640	0.54410	-1.58630
H	5.18290	-0.34480	0.75640
H	4.73310	-1.95230	0.18360

H	6.75320	-1.98570	-1.29680
H	7.19780	-0.37570	-0.72620
H	7.37300	-1.25470	1.61670
H	6.92780	-2.86440	1.04610
H	9.38010	-2.66380	1.29670
H	8.96460	-2.91800	-0.40700
H	9.41220	-1.29930	0.16670
H	-4.67440	-5.56080	-1.60710
H	-2.93280	-5.32450	-1.83000
H	-3.70350	-4.92260	-0.28360
H	-5.35240	-4.14620	-3.52110
H	-4.69910	-2.51590	-3.72690
H	-3.64990	-3.92570	-3.96190
H	-1.42550	-2.30460	2.55830
H	-2.18360	-3.33350	0.24510
H	-4.46720	-1.84530	0.38380
H	-1.39930	-0.68960	4.32860
H	-2.01730	0.59860	3.30460
H	0.77920	-0.52530	3.44930
H	0.30840	1.16570	3.26940
H	1.06350	-0.67670	1.23390
H	-2.04510	-2.92940	-2.25820
H	-3.12740	-1.56010	-2.08280
H	-4.96180	-3.08260	-1.32960

Standard orientations of **2ah**

C	1.84530	-2.75430	0.16610
C	0.95540	-3.37560	1.03410
C	-0.41180	-3.09690	0.96480
C	-0.86670	-2.17080	0.00320
C	0.02280	-1.50910	-0.88320
C	1.40350	-1.82970	-0.80090
C	-1.30910	-3.79750	1.91550
O	-2.61030	-3.46980	1.79390
O	-0.94040	-4.60630	2.75100
O	-2.20250	-1.87430	-0.11220
O	1.51720	-4.25490	1.92370
C	2.48010	-1.23220	-1.69590
C	3.17100	-0.00040	-1.07690
O	2.42040	1.17660	-1.36620
C	4.57400	0.18000	-1.67750
C	5.32600	1.42350	-1.18460
C	5.61780	1.40210	0.31730
C	6.47890	2.59640	0.73080
C	6.76780	2.58440	2.22350
O	-4.93140	0.51070	-1.27500

C	-3.01080	5.24480	0.95080
C	-2.58060	4.51610	3.30570
C	-3.77110	0.86010	-1.08680
C	-2.61920	0.63050	-2.07060
N	-1.36230	0.52700	-1.34090
C	-1.00420	1.43790	-0.36800
C	-2.16700	2.38470	0.04050
N	-3.43720	1.65470	-0.00850
C	-2.71600	-0.66460	-2.86330
C	-1.25910	-1.07950	-3.07840
C	-0.47780	-0.48190	-1.89590
O	0.14800	1.55190	0.04540
C	-1.90590	2.99160	1.42600
C	-2.93440	4.04270	1.89270
H	2.90370	-2.99970	0.24710
H	-3.05370	-4.01340	2.48460
H	-2.70130	-2.37270	0.56860
H	0.81290	-4.63950	2.48920
H	3.22490	-2.02110	-1.87130
H	2.07650	-1.00630	-2.68870
H	3.24840	-0.10370	0.01090
H	1.68560	1.22810	-0.71350
H	5.18030	-0.71100	-1.47360
H	4.48520	0.26530	-2.76850
H	6.27470	1.48410	-1.73270
H	4.76220	2.32880	-1.43940
H	4.67950	1.42650	0.88260
H	6.13250	0.46990	0.57930
H	7.42740	2.58030	0.18150
H	5.96860	3.53100	0.47060
H	7.38490	3.44590	2.49700
H	5.84040	2.63310	2.80310
H	7.30600	1.67570	2.51160
H	-3.68400	6.01030	1.35190
H	-2.02580	5.70230	0.80980
H	-3.39890	4.95760	-0.03090
H	-3.32260	5.23290	3.67330
H	-2.55820	3.67310	4.00440
H	-1.59940	5.00270	3.32870
H	-2.54890	1.49940	-2.73620
H	-2.20870	3.16530	-0.72770
H	-4.21010	1.88580	0.61780
H	-3.24410	-0.52330	-3.81190
H	-3.25730	-1.44160	-2.31370
H	-0.90080	-0.62430	-4.01080
H	-1.14680	-2.16330	-3.18340

H	0.36210	0.08290	-2.30310
H	-0.90830	3.45110	1.43480
H	-1.87100	2.17930	2.16510
H	-3.92810	3.58270	1.94220

Standard orientations of **2ba**

C	2.15890	-2.62200	-0.28690
C	1.56820	-3.18610	0.83820
C	0.21010	-2.99410	1.09070
C	-0.54610	-2.22160	0.18240
C	0.03580	-1.62300	-0.96550
C	1.42070	-1.83670	-1.19620
C	-0.46460	-3.57440	2.27660
O	0.34370	-4.29900	3.07590
O	-1.64640	-3.43200	2.54210
O	-1.89310	-2.02460	0.38160
O	2.40670	-3.91750	1.63580
C	2.19150	-1.28110	-2.38590
C	2.81750	0.12270	-2.22610
O	1.83880	1.13770	-2.43260
C	3.56520	0.35260	-0.90620
C	4.28570	1.70590	-0.87970
C	5.04920	1.90080	0.43180
C	5.76760	3.24950	0.46170
C	6.52710	3.44720	1.76380
O	-5.06570	0.01110	-0.37650
C	-3.12620	5.10310	0.75420
C	-2.09160	4.73030	3.00190
C	-3.92720	0.44610	-0.51140
C	-3.01610	0.14920	-1.70670
N	-1.61900	0.22930	-1.30020
C	-1.13350	1.28760	-0.55930
C	-2.25340	2.20460	0.00650
N	-3.42610	1.39760	0.35330
C	-3.17390	-1.25090	-2.28060
C	-1.77750	-1.60200	-2.79640
C	-0.79750	-0.78750	-1.93360
O	0.06670	1.53540	-0.45750
C	-1.73460	3.00970	1.20620
C	-2.71960	4.04760	1.78310
H	3.22130	-2.79810	-0.45250
H	-0.25480	-4.60100	3.79670
H	-2.18530	-2.45530	1.21400
H	1.88320	-4.25770	2.39160
H	3.00570	-1.98450	-2.61100
H	1.55400	-1.31100	-3.27750

H	3.53100	0.24100	-3.05090
H	1.33910	1.25350	-1.59260
H	2.86010	0.32890	-0.06670
H	4.29710	-0.44930	-0.75460
H	4.98400	1.76610	-1.72320
H	3.55950	2.51840	-1.00100
H	4.35160	1.83740	1.27590
H	5.78010	1.09230	0.55530
H	6.46940	3.31640	-0.37780
H	5.04070	4.06140	0.34260
H	7.03310	4.41770	1.76380
H	5.84840	3.41800	2.62220
H	7.28500	2.66890	1.89800
H	-3.75590	5.87210	1.21470
H	-2.24870	5.59820	0.32490
H	-3.70410	4.66100	-0.06290
H	-2.79120	5.44220	3.45280
H	-1.82770	3.99330	3.76780
H	-1.18240	5.27630	2.72800
H	-3.18490	0.91470	-2.47380
H	-2.54580	2.86960	-0.81420
H	-4.05090	1.66310	1.11640
H	-3.92350	-1.28270	-3.07800
H	-3.49190	-1.97130	-1.51970
H	-1.69720	-1.27000	-3.83970
H	-1.58960	-2.68040	-2.78540
H	-0.14500	-0.23890	-2.61280
H	-0.81010	3.52810	0.91840
H	-1.45220	2.30920	2.00430
H	-3.62660	3.53690	2.12670

Standard orientations of **2bb**

C	-1.21300	2.87630	-0.59000
C	-0.49530	3.30720	0.52010
C	0.74230	2.73940	0.82080
C	1.24690	1.72690	-0.02400
C	0.52540	1.25560	-1.15170
C	-0.73060	1.85440	-1.43380
C	1.54290	3.16080	1.99520
O	0.97750	4.13920	2.73010
O	2.62370	2.68790	2.30450
O	2.47410	1.15560	0.22270
O	-1.08960	4.29830	1.25380
C	-1.61860	1.48110	-2.61300
C	-2.63690	0.33900	-2.39630
O	-2.00770	-0.93020	-2.55220

C	-3.41120	0.40110	-1.07300
C	-4.49760	-0.67750	-0.98810
C	-5.27190	-0.58000	0.32800
C	-6.35430	-1.65530	0.41700
C	-7.12520	-1.56250	1.72410
O	4.88980	-1.84770	-0.33030
C	0.16490	-3.48990	3.81460
C	0.34310	-1.31980	2.59070
C	3.67110	-1.90460	-0.45630
C	2.89990	-1.40720	-1.68340
N	1.54230	-1.02550	-1.31710
C	0.73970	-1.83950	-0.54520
C	1.52160	-2.98270	0.15350
N	2.89920	-2.59460	0.45470
C	3.49130	-0.16380	-2.33070
C	2.27960	0.56720	-2.91130
C	1.08180	0.14690	-2.04120
O	-0.48390	-1.72840	-0.50600
C	0.79230	-3.54850	1.38250
C	0.89620	-2.73960	2.69590
H	-2.17370	3.34770	-0.79520
H	1.62860	4.29130	3.45260
H	2.86900	1.52670	1.04130
H	-0.49720	4.51440	2.00440
H	-2.18400	2.37990	-2.89800
H	-0.99140	1.26790	-3.48670
H	-3.35790	0.40340	-3.22060
H	-1.57520	-1.16010	-1.69850
H	-2.72510	0.25590	-0.22980
H	-3.87380	1.38900	-0.96460
H	-5.19040	-0.56730	-1.83100
H	-4.04380	-1.67240	-1.06880
H	-4.57820	-0.68650	1.17090
H	-5.73180	0.41220	0.41130
H	-7.05320	-1.55070	-0.42100
H	-5.89920	-2.64950	0.33800
H	-7.89400	-2.34040	1.76690
H	-6.45920	-1.69550	2.58270
H	-7.61970	-0.59050	1.81980
H	0.27020	-2.96460	4.76980
H	-0.90430	-3.58500	3.59620
H	0.57650	-4.49670	3.94270
H	0.36290	-0.82230	3.56660
H	0.94500	-0.71010	1.91270
H	-0.69290	-1.31920	2.23670
H	2.82860	-2.23150	-2.40380

H	1.58960	-3.78190	-0.59750
H	3.41040	-3.01750	1.23190
H	4.22470	-0.41580	-3.10370
H	4.00680	0.47010	-1.60170
H	2.11770	0.21380	-3.93790
H	2.43350	1.64980	-2.96320
H	0.30560	-0.22100	-2.71230
H	1.21280	-4.54470	1.57950
H	-0.26530	-3.71200	1.13860
H	1.95080	-2.67060	2.98920

Standard orientations of **2bc**

C	2.12960	-2.65330	-0.27260
C	1.52510	-3.20710	0.84960
C	0.16720	-2.99640	1.09770
C	-0.57370	-2.21530	0.18700
C	0.02260	-1.62670	-0.95970
C	1.40560	-1.85970	-1.18540
C	-0.41820	-3.61600	2.31140
O	-1.72920	-3.35990	2.48740
O	0.19990	-4.30350	3.10730
O	-1.91620	-1.99750	0.37740
O	2.34810	-3.95160	1.65490
C	2.18820	-1.31740	-2.37360
C	2.83260	0.07820	-2.21550
O	1.86890	1.10550	-2.43190
C	3.57580	0.30300	-0.89220
C	4.31430	1.64650	-0.86650
C	5.07280	1.83610	0.44870
C	5.80910	3.17510	0.47770
C	6.56370	3.36750	1.78350
O	-5.04290	0.04650	-0.35770
C	-3.07010	5.14360	0.72200
C	-2.03070	4.78000	2.96900
C	-3.90590	0.48120	-0.50630
C	-3.00460	0.17810	-1.70780
N	-1.60560	0.24710	-1.30550
C	-1.10880	1.30380	-0.56960
C	-2.21980	2.23380	-0.00720
N	-3.39630	1.43640	0.34990
C	-3.17610	-1.21970	-2.28380
C	-1.78270	-1.58460	-2.79850
C	-0.79500	-0.78160	-1.93370
O	0.09360	1.53970	-0.46850
C	-1.69130	3.04410	1.18470
C	-2.66730	4.09280	1.75720

H	3.19010	-2.84490	-0.43280
H	-1.95040	-3.83510	3.32070
H	-2.19570	-2.42840	1.21210
H	1.83280	-4.29600	2.41640
H	2.99380	-2.03240	-2.59380
H	1.55380	-1.34140	-3.26760
H	3.55250	0.18350	-3.03660
H	1.36840	1.23390	-1.59430
H	2.86560	0.29190	-0.05660
H	4.29590	-0.50820	-0.73330
H	5.01820	1.69400	-1.70620
H	3.59990	2.46830	-0.99510
H	4.36950	1.78530	1.28890
H	5.79200	1.01830	0.57950
H	6.51660	3.22930	-0.35790
H	5.09400	3.99630	0.35140
H	7.08280	4.33110	1.78280
H	5.87970	3.35070	2.63800
H	7.31030	2.57950	1.92500
H	-3.69310	5.92020	1.17900
H	-2.19070	5.62960	0.28630
H	-3.65370	4.69960	-0.08990
H	-2.72390	5.50000	3.41690
H	-1.76930	4.04680	3.73940
H	-1.11860	5.31780	2.68820
H	-3.17080	0.94630	-2.47280
H	-2.51060	2.89470	-0.83190
H	-4.01560	1.71100	1.11410
H	-3.92470	-1.24210	-3.08250
H	-3.50320	-1.93810	-1.52510
H	-1.69810	-1.25160	-3.84120
H	-1.60600	-2.66490	-2.78910
H	-0.13220	-0.24360	-2.61120
H	-0.76430	3.55400	0.88980
H	-1.41080	2.34770	1.98700
H	-3.57660	3.59090	2.10740

Standard orientations of **2bd**

C	-1.19180	2.85790	-0.65350
C	-0.45620	3.29840	0.44050
C	0.77550	2.71560	0.74500
C	1.25580	1.67900	-0.08130
C	0.51600	1.19850	-1.19470
C	-0.73340	1.81090	-1.47880
C	1.51000	3.22790	1.92710
O	2.67710	2.59980	2.16970

O	1.12330	4.13440	2.64590
O	2.47350	1.09280	0.16310
O	-1.02460	4.31690	1.16110
C	-1.63830	1.42480	-2.64090
C	-2.67130	0.30320	-2.38850
O	-2.06300	-0.97890	-2.52110
C	-3.43210	0.40700	-1.06010
C	-4.53430	-0.65220	-0.94120
C	-5.29480	-0.51320	0.37920
C	-6.39280	-1.56920	0.50220
C	-7.14990	-1.43520	1.81380
O	4.85670	-1.89000	-0.33070
C	1.57190	-1.54990	3.09330
C	-0.37980	-2.97970	3.71600
C	3.63660	-1.94670	-0.44370
C	2.85630	-1.49500	-1.68200
N	1.50430	-1.09420	-1.31710
C	0.70330	-1.87300	-0.51020
C	1.48410	-2.97780	0.24610
N	2.87130	-2.59070	0.50450
C	3.44740	-0.27770	-2.37850
C	2.23370	0.44490	-2.96520
C	1.04520	0.06190	-2.06670
O	-0.52060	-1.76190	-0.47700
C	0.76500	-3.43150	1.52670
C	0.38000	-2.33650	2.55010
H	-2.14620	3.34130	-0.85960
H	3.02350	3.05250	2.97240
H	2.86060	1.48410	0.97410
H	-0.43330	4.55660	1.90730
H	-2.19300	2.32530	-2.94080
H	-1.02280	1.18220	-3.51530
H	-3.39910	0.35970	-3.20740
H	-1.62530	-1.19690	-1.66710
H	-2.74040	0.26980	-0.22020
H	-3.87830	1.40430	-0.96970
H	-5.23320	-0.54990	-1.78010
H	-4.09690	-1.65570	-1.00370
H	-4.59500	-0.61170	1.21810
H	-5.73850	0.48770	0.44440
H	-7.09780	-1.47230	-0.33160
H	-5.95390	-2.57190	0.44130
H	-7.93010	-2.19980	1.88080
H	-6.47800	-1.55950	2.66900
H	-7.62840	-0.45380	1.89210
H	1.25940	-0.87890	3.90110

H	2.34270	-2.21790	3.49140
H	2.01990	-0.92120	2.31900
H	-0.71410	-2.21960	4.43020
H	-1.26830	-3.51000	3.35690
H	0.25030	-3.69640	4.25370
H	2.77550	-2.34560	-2.36990
H	1.54070	-3.83370	-0.43960
H	3.38830	-2.98370	1.29330
H	4.16740	-0.56100	-3.15330
H	3.97840	0.37590	-1.67850
H	2.05510	0.06100	-3.97800
H	2.39510	1.52410	-3.05280
H	0.25420	-0.31450	-2.71500
H	1.38210	-4.18890	2.02870
H	-0.15900	-3.94340	1.22460
H	-0.30400	-1.62290	2.07660

Standard orientations of **2be**

C	-1.21320	2.83530	-0.69030
C	-0.48530	3.29620	0.40110
C	0.74930	2.72830	0.71370
C	1.24130	1.68530	-0.10080
C	0.50980	1.18450	-1.20960
C	-0.74400	1.78240	-1.50270
C	1.55960	3.18040	1.86990
O	1.00750	4.18850	2.57420
O	2.63710	2.70710	2.19010
O	2.46640	1.11420	0.15640
O	-1.06750	4.31550	1.10550
C	-1.64360	1.37380	-2.66120
C	-2.66730	0.24670	-2.39580
O	-2.04720	-1.03170	-2.50640
C	-3.43410	0.36390	-1.07190
C	-4.52770	-0.70290	-0.94140
C	-5.29470	-0.55070	0.37380
C	-6.38410	-1.61410	0.50830
C	-7.14770	-1.46690	1.81470
O	4.88050	-1.89250	-0.33180
C	1.61450	-1.49790	3.11340
C	-0.33510	-2.91790	3.76470
C	3.65900	-1.94320	-0.43150
C	2.87020	-1.50100	-1.66750
N	1.51850	-1.10080	-1.30130
C	0.72380	-1.87280	-0.48180
C	1.51140	-2.96470	0.28600
N	2.89960	-2.57320	0.53040

C	3.45520	-0.28620	-2.37340
C	2.23790	0.42470	-2.96680
C	1.05120	0.04210	-2.06570
O	-0.50070	-1.76690	-0.44640
C	0.80030	-3.40090	1.57710
C	0.41980	-2.29140	2.58650
H	-2.17170	3.30690	-0.90490
H	1.66360	4.35840	3.28810
H	2.86860	1.50790	0.96070
H	-0.46850	4.55190	1.84470
H	-2.20530	2.26570	-2.97370
H	-1.02450	1.12500	-3.53130
H	-3.39240	0.28440	-3.21810
H	-1.60760	-1.23100	-1.64870
H	-2.74460	0.24520	-0.22740
H	-3.88920	1.35850	-0.99810
H	-5.22400	-0.61930	-1.78450
H	-4.08140	-1.70340	-0.98700
H	-4.59750	-0.63040	1.21690
H	-5.74720	0.44730	0.42210
H	-7.08650	-1.53590	-0.32960
H	-5.93640	-2.61380	0.46430
H	-7.92160	-2.23710	1.89020
H	-6.47830	-1.57250	2.67440
H	-7.63490	-0.48860	1.87630
H	1.30570	-0.81540	3.91300
H	2.38660	-2.16060	3.51770
H	2.05950	-0.88050	2.32840
H	-0.66580	-2.14770	4.46970
H	-1.22530	-3.45270	3.41700
H	0.29710	-3.62730	4.30960
H	2.78870	-2.35580	-2.35010
H	1.56500	-3.83040	-0.38750
H	3.42220	-2.95430	1.32150
H	4.17790	-0.57140	-3.14500
H	3.98110	0.37590	-1.67750
H	2.06200	0.03190	-3.97660
H	2.39340	1.50410	-3.06270
H	0.26500	-0.34940	-2.71110
H	1.42100	-4.15030	2.08650
H	-0.12490	-3.91800	1.28760
H	-0.26600	-1.58430	2.10600

Standard orientations of **2bf**

C	3.08590	-1.44510	-0.38620
C	2.81240	-2.13710	0.78760

C	1.50160	-2.49700	1.10690
C	0.46730	-2.14680	0.21450
C	0.71780	-1.42830	-0.98470
C	2.06150	-1.07650	-1.28200
C	1.27630	-3.23510	2.37340
O	-0.01370	-3.53840	2.61650
O	2.15550	-3.55750	3.15520
O	-0.83480	-2.49640	0.47570
O	3.90160	-2.42520	1.56890
C	2.49940	-0.32040	-2.52920
C	2.49950	1.22330	-2.45310
O	1.18810	1.73720	-2.67190
C	3.10210	1.80950	-1.16860
C	3.19050	3.33990	-1.22050
C	3.89810	3.94640	-0.00480
C	3.13370	3.74660	1.30480
C	3.81230	4.46930	2.45840
O	-4.55150	-1.97680	-0.13440
C	-4.84550	3.53170	0.64770
C	-3.65290	3.76130	2.83560
C	-3.70740	-1.11840	-0.36800
C	-2.81290	-1.08820	-1.61180
N	-1.55540	-0.42130	-1.29920
C	-1.51630	0.78760	-0.63450
C	-2.88930	1.20190	-0.03560
N	-3.60830	0.00950	0.42120
C	-2.40890	-2.46130	-2.12770
C	-1.01220	-2.24440	-2.71220
C	-0.41540	-1.05600	-1.93780
O	-0.52070	1.50870	-0.61910
C	-2.69680	2.22470	1.09290
C	-3.99600	2.80400	1.69020
H	4.12210	-1.18560	-0.60060
H	0.01640	-4.01530	3.47720
H	-0.87590	-2.95760	1.33950
H	3.60750	-2.91000	2.37040
H	3.52360	-0.64080	-2.76790
H	1.90390	-0.65710	-3.38600
H	3.09180	1.57780	-3.30570
H	0.70250	1.69950	-1.81670
H	2.47850	1.52840	-0.31320
H	4.10380	1.39390	-1.00990
H	3.74330	3.63540	-2.12070
H	2.18690	3.77270	-1.30890
H	4.90510	3.52120	0.08510
H	4.02470	5.02120	-0.18510

H	2.10870	4.12050	1.19960
H	3.06930	2.68120	1.55020
H	3.25320	4.31080	3.38600
H	4.83130	4.09850	2.60810
H	3.86190	5.54680	2.27210
H	-5.71560	4.00400	1.11670
H	-4.26940	4.31350	0.14130
H	-5.22460	2.84000	-0.11040
H	-4.56340	4.15250	3.30190
H	-3.07590	3.24800	3.61210
H	-3.06210	4.61230	2.47980
H	-3.31670	-0.50340	-2.39110
H	-3.46470	1.63420	-0.86220
H	-4.25340	0.04530	1.21220
H	-3.11130	-2.83700	-2.87890
H	-2.37630	-3.20690	-1.32650
H	-1.11600	-1.96690	-3.76920
H	-0.40070	-3.15170	-2.67510
H	-0.06440	-0.33220	-2.67320
H	-2.08140	3.05610	0.72330
H	-2.11660	1.75450	1.89880
H	-4.59620	1.99050	2.11380

Standard orientations of **2bg**

C	-0.44900	-3.25000	0.58210
C	0.31300	-3.57220	-0.53480
C	1.39600	-2.77290	-0.90600
C	1.69970	-1.63920	-0.12440
C	0.92970	-1.28050	1.01380
C	-0.16610	-2.11310	1.36620
C	2.16950	-3.17110	-2.10720
O	3.19130	-2.34580	-2.40750
O	1.93270	-4.15250	-2.79200
O	2.76680	-0.83410	-0.43830
O	-0.07700	-4.70100	-1.20820
C	-1.07890	-1.87770	2.56210
C	-2.31420	-0.97610	2.33940
O	-1.95040	0.40030	2.40660
C	-3.10550	-1.26450	1.05680
C	-4.38890	-0.42990	0.97020
C	-5.17300	-0.75100	-0.30370
C	-6.45050	0.08300	-0.39510
C	-7.23190	-0.23190	-1.66060
O	4.53330	2.51100	-0.15410
C	-2.05870	4.16180	-2.61050
C	-1.08370	5.43420	-0.69160

C	3.33420	2.37020	0.06000
C	2.73650	1.81670	1.35790
N	1.46280	1.16840	1.07370
C	0.49280	1.76980	0.29680
C	0.99210	3.05450	-0.42170
N	2.39410	2.86740	-0.82040
C	3.58020	0.74370	2.03000
C	2.55450	-0.18170	2.68660
C	1.27230	-0.04720	1.84630
O	-0.67190	1.37820	0.27090
C	0.13560	3.40530	-1.64640
C	-1.23010	4.05010	-1.32710
H	-1.28350	-3.90110	0.84030
H	3.58760	-2.74830	-3.21380
H	3.19250	-1.16960	-1.25490
H	0.51720	-4.84230	-1.97690
H	-1.44090	-2.85890	2.90070
H	-0.48460	-1.50340	3.40410
H	-2.97810	-1.13870	3.19750
H	-1.59850	0.66190	1.52540
H	-2.49230	-1.03210	0.17770
H	-3.36280	-2.32940	1.01580
H	-5.01590	-0.62940	1.84760
H	-4.14180	0.63830	0.98400
H	-4.54400	-0.55640	-1.18100
H	-5.42830	-1.81760	-0.31930
H	-7.08530	-0.11110	0.47730
H	-6.20020	1.15030	-0.38340
H	-8.14130	0.37540	-1.70560
H	-6.63570	-0.01760	-2.55330
H	-7.52570	-1.28610	-1.68820
H	-3.04420	4.59150	-2.40170
H	-1.56160	4.79740	-3.35120
H	-2.21530	3.17520	-3.05930
H	-2.06480	5.90000	-0.54740
H	-0.60800	5.37620	0.29150
H	-0.48480	6.09990	-1.32220
H	2.54130	2.65380	2.03920
H	0.99230	3.86440	0.31480
H	2.77070	3.31940	-1.65600
H	4.27240	1.17020	2.76340
H	4.18890	0.18890	1.30850
H	2.35630	0.18310	3.70280
H	2.92060	-1.20930	2.77810
H	0.45680	0.17990	2.53270
H	-0.02460	2.49350	-2.23780

H	0.69470	4.09140	-2.29740
H	-1.78760	3.41420	-0.63120

Standard orientations of **2bh**

C	3.08730	-1.42610	-0.40110
C	2.82620	-2.11750	0.77650
C	1.52080	-2.48470	1.10180
C	0.47850	-2.14320	0.21250
C	0.71640	-1.42570	-0.98880
C	2.05600	-1.06550	-1.29280
C	1.19210	-3.22130	2.34580
O	2.25780	-3.50400	3.12150
O	0.06800	-3.56050	2.67560
O	-0.82100	-2.50380	0.48440
O	3.92210	-2.39360	1.54910
C	2.48240	-0.30740	-2.54270
C	2.47300	1.23630	-2.46720
O	1.15660	1.74180	-2.67530
C	3.08210	1.82670	-1.18770
C	3.16040	3.35760	-1.24080
C	3.87410	3.96900	-0.03120
C	3.12180	3.76480	1.28470
C	3.80530	4.49210	2.43260
O	-4.57390	-1.98820	-0.14680
C	-4.85690	3.50830	0.67760
C	-3.66350	3.72390	2.86650
C	-3.72220	-1.13430	-0.36850
C	-2.82090	-1.10250	-1.60690
N	-1.56500	-0.43390	-1.29160
C	-1.52860	0.77310	-0.62360
C	-2.90140	1.18250	-0.02120
N	-3.62140	-0.01180	0.42780
C	-2.41250	-2.47580	-2.11870
C	-1.01730	-2.25560	-2.70550
C	-0.42450	-1.06230	-1.93540
O	-0.53500	1.49750	-0.60900
C	-2.70840	2.19800	1.11380
C	-4.00720	2.77390	1.71520
H	4.12040	-1.15940	-0.62220
H	1.86570	-3.98280	3.88700
H	-0.87700	-2.96810	1.34750
H	3.61540	-2.87470	2.34630
H	3.50720	-0.62160	-2.78690
H	1.88420	-0.64820	-3.39610
H	3.05610	1.59430	-3.32460
H	0.67700	1.69780	-1.81700

H	2.46700	1.54210	-0.32730
H	4.08760	1.41740	-1.03680
H	3.70400	3.65630	-2.14570
H	2.15340	3.78410	-1.32110
H	4.88460	3.55030	0.05060
H	3.99250	5.04450	-0.21280
H	2.09370	4.13220	1.18790
H	3.06620	2.69910	1.53090
H	3.25490	4.33050	3.36470
H	4.82790	4.12790	2.57390
H	3.84660	5.56990	2.24550
H	-5.72670	3.97790	1.14980
H	-4.28080	4.29310	0.17590
H	-5.23650	2.82150	-0.08470
H	-4.57380	4.11240	3.33560
H	-3.08640	3.20560	3.63960
H	-3.07260	4.57700	2.51580
H	-3.32200	-0.51960	-2.38920
H	-3.47680	1.62040	-0.84490
H	-4.26760	0.01920	1.21820
H	-3.11450	-2.85710	-2.86750
H	-2.37550	-3.21780	-1.31430
H	-1.12310	-1.98200	-3.76340
H	-0.40250	-3.16040	-2.66590
H	-0.08250	-0.33710	-2.67370
H	-2.09290	3.03160	0.74960
H	-2.12820	1.72240	1.91660
H	-4.60750	1.95790	2.13400

Standard orientations of **2bi**

C	-0.47910	-3.23840	0.59460
C	0.27390	-3.57340	-0.52510
C	1.36310	-2.78780	-0.90100
C	1.68310	-1.65450	-0.12190
C	0.92270	-1.28320	1.01750
C	-0.18090	-2.10240	1.37520
C	2.19510	-3.10320	-2.08700
O	1.81020	-4.20770	-2.75720
O	3.15310	-2.44590	-2.45800
O	2.76130	-0.86340	-0.44510
O	-0.13450	-4.69770	-1.19090
C	-1.08760	-1.85290	2.57290
C	-2.31350	-0.93850	2.35020
O	-1.93410	0.43400	2.40800
C	-3.11310	-1.22500	1.07230
C	-4.38750	-0.37670	0.98630

C	-5.18030	-0.69590	-0.28270
C	-6.44880	0.15180	-0.37350
C	-7.23880	-0.16140	-1.63410
O	4.56510	2.50380	-0.14150
C	-2.00870	4.15660	-2.64170
C	-1.03600	5.43420	-0.72510
C	3.36470	2.35760	0.06180
C	2.75970	1.80100	1.35460
N	1.48310	1.15970	1.06720
C	0.51860	1.76590	0.28740
C	1.02700	3.04540	-0.43380
N	2.43020	2.85330	-0.82470
C	3.59680	0.72040	2.02260
C	2.56560	-0.19760	2.68090
C	1.28230	-0.05140	1.84420
O	-0.64900	1.38270	0.26220
C	0.17760	3.39450	-1.66410
C	-1.18630	4.04750	-1.35410
H	-1.32040	-3.87850	0.85840
H	2.45650	-4.26550	-3.49740
H	3.19710	-1.19080	-1.26150
H	0.46360	-4.82860	-1.95650
H	-1.45930	-2.82890	2.91610
H	-0.48690	-1.48200	3.41180
H	-2.97570	-1.08890	3.21170
H	-1.57970	0.68580	1.52500
H	-2.50100	-1.00420	0.18960
H	-3.38240	-2.28720	1.03820
H	-5.01310	-0.56440	1.86730
H	-4.12850	0.68870	0.99330
H	-4.55270	-0.51310	-1.16350
H	-5.44750	-1.75970	-0.29150
H	-7.08210	-0.03050	0.50250
H	-6.18670	1.21620	-0.36870
H	-8.14160	0.45580	-1.67880
H	-6.64390	0.04150	-2.53040
H	-7.54440	-1.21230	-1.65480
H	-2.99300	4.59210	-2.43950
H	-1.50530	4.78590	-3.38350
H	-2.16810	3.16840	-3.08610
H	-2.01540	5.90540	-0.58760
H	-0.56490	5.37900	0.26030
H	-0.43120	6.09370	-1.35660
H	2.56760	2.63570	2.03950
H	1.02620	3.85810	0.29970
H	2.81290	3.30220	-1.65930

H	4.29510	1.14000	2.75420
H	4.19790	0.16160	1.29780
H	2.37320	0.16700	3.69830
H	2.92380	-1.22830	2.76960
H	0.47190	0.18720	2.53280
H	0.01570	2.48070	-2.25180
H	0.74290	4.07480	-2.31590
H	-1.75000	3.41790	-0.65740
